# Beyond Accuracy: Multi-Level Ordinal Assessment of Lumbar Spine Degeneration from Multiplanar MRI Using the RSNA 2024 (LumbarDISC) Dataset and Condition-Specific Models

**DOI:** 10.3390/s26216810

**Published:** 2026-10-28

**Authors:** Emanuel-Crăciun Trînc, Cosmin Ancuți, Emil-Radu Iacob, Codruța-Orniana Ancuți

**Affiliations:** 1 Department of Communications, Polytechnic University of Timișoara, 30006 Timișoara, Romania; emanuel.trinc@yahoo.com (E.-C.T.); cosmin.ancuti@upt.ro (C.A.); 2 Department of Pediatric Surgery, ‘Victor Babeș’ University of Medicine and Pharmacy, 300041 Timișoara, Romania; 3 Center of Multidisciplinary Research and Management in Pediatric Surgical Conditions (CCMMA-CHIRPED), Iosif Nemoianu 2, 300011 Timișoara, Romania

**Keywords:** lumbar spine MRI, degenerative disease assessment, ordinal classification, condition-specific modeling, spinal-level analysis, patient-level evaluation, 2D and 2.5D-to-3D learning, RSNA 2024 LumbarDISC, deep learning, medical image analysis

## Abstract

Automated assessment of lumbar spine degeneration from magnetic resonance imaging (MRI) is commonly evaluated using aggregate classification metrics, potentially overlooking clinically relevant differences in severity misclassification, anatomical variability, and patient-level performance. This study proposes a Beyond-Accuracy Multi-Dimensional Evaluation Framework using the RSNA 2024 LumbarDISC dataset. The framework integrates six complementary dimensions: exact classification, ordinal severity, condition-specific analysis, spinal-level analysis, patient-level assessment, and reliability and robustness, alongside comparisons of imaging representations. To demonstrate its applicability, condition-specific models were developed for five lumbar degenerative conditions using ViT, MaxViT, ConvNeXt, and EfficientNet architectures with 2D and cross-level 2.5D-to-3D inputs. Experimental findings reveal substantial performance variation across conditions, spinal levels, and architectures, demonstrating that similar aggregate accuracy can conceal differences in ordinal error severity and patient-level error patterns. The evaluated 2D models generally outperformed their 2.5D-to-3D counterparts, suggesting that additional cross-level context does not necessarily improve classification. Overall, the proposed framework provides a structured approach to evaluating lumbar MRI models beyond aggregate accuracy, supporting more informative model comparison and clinically oriented assessments.

## 1. Introduction

Low back pain is among a leading cause of disability worldwide and represents a substantial clinical and socioeconomic burden. Estimates from the Global Burden of Disease Study 2021 indicate that approximately 619 million people experienced low back pain in 2020, with the affected population projected to exceed 840 million by 2050 [1]. Lumbar degenerative abnormalities involve several anatomical structures and can include spinal canal stenosis, neural foraminal narrowing, and subarticular stenosis. These findings may coexist at multiple spinal levels and can be associated with radiculopathy, neurogenic claudication, and functional impairment [2,3].

Magnetic resonance imaging (MRI) is central to the assessment of lumbar degeneration because it visualizes the spinal canal, neural elements, intervertebral discs, foramina, and adjacent soft tissues [4,5,6]. Nevertheless, the interpretation of lumbar MRI requires attention to both the anatomical location and the specific degenerative condition. Normal canal dimensions vary across spinal levels and demographic groups [7], whereas established grading systems describe progressive anatomical compromise in the central canal and neural foramina [8,9]. Although standardized grading can support clinical interpretation, disagreement between readers remains a challenge [10,11,12]. Efforts to standardize quantitative vertebral measurements further illustrate the importance of reproducible anatomical assessments [13].

Deep learning has increasingly been investigated for automated lumbar MRI analysis, including localization, stenosis detection, severity classification, and image interpretation. Hallinan et al. developed a system for automated assessment of the central canal, lateral recess, and neural foraminal stenosis [14], while Bharadwaj et al. explored interpretable classification of lumbar stenosis and facet arthropathy from axial MRI [15]. More recent attention-based and multistage approaches have expanded the range of automated lumbar assessment strategies [16,17]. Architectural developments have also included combinations of convolutional and Transformer representations for lumbar MRI analysis [18] and vision–language approaches to automated lumbar spine reporting [19]. These studies motivate systematic comparison of model families, but predictive performance must be interpreted in relation to the anatomical and ordinal structure of the target task.

The Radiological Society of North America (RSNA) 2024 Lumbar Spine Degenerative Classification dataset, subsequently described as the Lumbar Degenerative Imaging Spine Classification (LumbarDISC) dataset, provides a multicenter resource for evaluating these questions [20]. It comprises examinations from 2697 patients and 8593 MRI series from eight institutions across six countries and five continents, with sagittal T1-weighted, sagittal T2-weighted or T2-like/STIR, and axial T2-weighted acquisitions [20]. Expert annotations cover five degenerative conditions: spinal canal stenosis (SCS), left and right neural foraminal narrowing (LFN and RFN), and left and right subarticular stenosis (LSS and RSS). Each condition is assessed at five lumbar levels, from L1/L2 through L5/S1, using three ordered severity categories: Normal/Mild, Moderate, and Severe. Thus, a complete examination may contain up to 25 condition–level severity assessments.

In this study, *multiplanar MRI* refers to the sagittal and axial acquisitions available in LumbarDISC and processed through condition-specific pipelines. The term describes the imaging data used across the study rather than explicit fusion of different MRI planes within an individual model.

This structure poses two related challenges for evaluation. First, the severity categories are ordered rather than nominal: confusing Moderate with Severe is not equivalent, in ordinal distance, to confusing Normal/Mild with Severe. Conventional accuracy and Macro-$F_{1}$ characterize exact classification and class balance, respectively, but they do not explicitly quantify the magnitude of a severity disagreement. Second, the distribution of labels is imbalanced, with Normal/Mild findings more common than Severe findings [20]. A high pooled accuracy can therefore conceal poor performance for less frequent grades or particular condition–level combinations. These limitations motivate a multi-dimensional assessment that retains established classification measures while characterizing ordinal errors and their anatomical distribution.

In this work, we use the term *Beyond Accuracy* to denote evaluation at five complementary levels. *Exact classification* is measured using accuracy, Macro-Precision, Macro-Recall, and Macro-$F_{1}$. *Ordinal severity* is characterized using Ordinal Mean Absolute Error (O-MAE), Quadratic Weighted Kappa (QWK), and Severe Error Rate (SER), together with exact-match Ordinal Accuracy (OA). For the same set of three-class predictions, OA and conventional accuracy represent the same exact-match proportion; OA is retained for consistency with the ordinal analysis rather than claimed as an independent source of information. *Condition-specific evaluation* compares performance across SCS, LFN, RFN, LSS, and RSS. *Spinal-level evaluation* examines differences across the five anatomical levels. Finally, *patient-level evaluation* aggregates condition–level predictions within examinations to characterize exactness, accumulated ordinal error, and the occurrence of maximum-distance disagreements. These levels distinguish pooled predictive performance from the distribution and severity of mistakes across the lumbar anatomy.

To examine the effect of architecture and input representation, the study considers four model families—Vision Transformer (ViT), MaxViT, ConvNeXt, and EfficientNet—in two experimental settings. The first uses localized two-dimensional regions of interest for condition-specific severity classification. The second uses a cross-level 2.5D-to-3D representation that stacks five localized ROIs corresponding to the anatomically ordered lumbar levels (L1/L2–L5/S1) along an artificial depth dimension for joint processing by a 3D encoder. Unlike conventional 2.5D approaches that stack neighboring MRI slices, this representation captures cross-level anatomical context without reconstructing a spatially contiguous MRI volume or performing explicit sagittal–axial feature fusion. Although the source dataset contains multiple imaging planes and sequences, *explicit multi-plane fusion is not an experimental contribution of the present study*. Geometry-aware volumetric reconstruction and true multi-plane fusion are reserved for future investigation.

The cross-level representation was included as an exploratory architectural comparison rather than as a proposed substitute for geometry-aware volumetric MRI modeling. Its inclusion enables the Beyond-Accuracy framework to be examined across both localized 2D inputs and structured multi-level inputs, allowing the benefits and limitations of additional anatomical context to be assessed empirically.

An important methodological distinction is the separation between model development and independent performance assessment. The data-partitioning protocol defines disjoint training, validation, and held-out test partitions at the patient/study grouping level, subject to the grouping identifier used in the implementation. Training data support parameter optimization, and validation performance guides architecture and hyperparameter selection. The held-out test partition is intended solely for final assessment of selected models and must not influence model selection. Accordingly, validation-based architecture comparisons and any independent test-set findings are identified separately throughout the manuscript. Claims about generalization are restricted to evaluations actually performed on held-out data.

The principal contributions of the present study are summarized as follows:

1. **Condition-specific severity modeling.** We investigate lumbar degeneration severity classification for five conditions using four representative convolutional and Transformer-based architecture families.
2. **Comparison of spatial input representations.** We compare localized 2D modeling with a cross-level 2.5D-to-3D representation and distinguish the latter from true volumetric reconstruction or multi-plane fusion.
3. **Ordinal-aware evaluation.** We organize conventional classification metrics alongside O-MAE, QWK, and SER to assess not only whether a severity prediction is incorrect, but also the magnitude of its ordinal disagreement.
4. **Anatomically structured performance analysis.** We examine condition-specific and spinal-level performance to reveal weaknesses that may be obscured by pooled results.
5. **Examination-level error characterization.** We aggregate condition–level predictions by examination to study exact predictions, adjacent-grade disagreements, and the burden of maximum-distance severity errors.
6. **Architecture-search and repeated-run analysis.** We document the architecture and configuration search, repeated training experiments, and GPU-parallel execution strategy, while distinguishing validation-driven selection from subsequent evaluation.

The objective is not simply to identify the architecture with the highest pooled accuracy, but to establish a structured view of prediction quality: whether performance is balanced across grades, how severe the errors are, which conditions and spinal levels remain difficult, and how errors accumulate within individual examinations. The study therefore focuses on the empirical assessment of imaging-based degeneration severity rather than direct prediction of pain, symptoms, or clinical outcomes. Broader volumetric modeling, explicit multi-plane fusion, and clinical decision-support integration remain directions for future work.

## 2. Related Work

### 2.1. Automated Analysis of Lumbar Spine MRI

Deep learning has increasingly been applied to lumbar MRI for anatomical localization, detection of degenerative abnormalities, severity grading, and quantitative image analysis. Hallinan et al. developed a deep-learning framework for automated detection and classification of central canal, lateral recess, and neural foraminal stenosis [14]. Bharadwaj et al. subsequently proposed an interpretable approach for automated classification of lumbar spinal stenosis and facet arthropathy from axial MRI [15]. Attention mechanisms have also been investigated for lumbar stenosis classification [16], while multistage systems have combined anatomical localization, level identification, and severity classification [17].

Automated lumbar MRI analysis extends beyond stenosis classification. Stojšić et al. proposed a hybrid CNN–Transformer network for vertebral bone-quality determination from T1-weighted lumbar MRI [18], illustrating the use of complementary local convolutional features and Transformer-based representations in lumbar imaging. Masrur et al. investigated semi-automated standardized vertebral measurement with an emphasis on reproducibility [13]. These studies reflect a broader transition from qualitative visual assessment toward reproducible computational characterization of lumbar anatomy.

Anatomical variability is an important consideration for such systems. Baştuğ investigated lumbar canal width in asymptomatic subjects using MRI and reported variation associated with anatomical and demographic factors [7]. This reinforces the need to evaluate automated systems across anatomical levels rather than assuming homogeneous task difficulty throughout the lumbar column.

Several investigations have also considered multiplanar information. Different degenerative findings are preferentially evaluated in sagittal and axial MRI, motivating systems that integrate complementary views. Batra et al. proposed M-SCAN, a multistage cross-view model combining sagittal and axial MRI and reported an AUROC of 0.971 for spinal canal stenosis grading [21]. Such work demonstrates the potential value of additional spatial context; however, whether additional image context consistently translates into improved ordinal severity estimation remains an open question.

In the present work, this issue is examined through a comparison between single-image 2D representations and cross-level 2.5D-to-3D inputs. The latter stack five localized ROIs corresponding to the anatomically ordered lumbar levels (L1/L2–L5/S1) and process the resulting tensor using 3D operations. Unlike conventional 2.5D approaches based on neighboring MRI slices, this representation captures cross-level anatomical context without reconstructing a spatially contiguous MRI volume or performing explicit multiplanar feature fusion.

### 2.2. Ordinal and Structured Evaluation of Lumbar Degeneration

Most automated classification studies report conventional measures such as Accuracy, sensitivity, specificity, precision, recall, $F_{1}$-score, or AUROC. These metrics remain essential but do not fully represent the structure of an ordered three-grade degeneration task. In particular, nominal classification metrics generally assign the same incorrect status to an adjacent Normal/Mild–Moderate disagreement and a maximum-distance Normal/Mild–Severe disagreement.

Lumbar stenosis grading systems are inherently ordinal [8,9]. Weighted agreement statistics such as quadratic weighted Cohen’s κ therefore provide a natural complement to exact classification metrics because disagreements can be weighted according to their magnitude. Nevertheless, a single ordinal agreement value does not identify which pathological conditions or anatomical levels produce the observed errors.

This consideration is especially important for LumbarDISC, where five degenerative conditions are assessed independently across five lumbar levels using three ordered severity categories [20]. The resulting structure can be regarded as a condition-by-level severity matrix for each patient. Evaluation can therefore occur at several nested scales: individual classification, severity-distance error, degenerative condition, anatomical level, and complete patient profile.

The present study operationalizes this hierarchy using exact classification metrics together with OA, O-MAE, QWK, and SER. Condition-specific analysis then identifies the weakest pathology and between-condition variability, while spinal-level analysis measures anatomical performance heterogeneity. Finally, patient-level aggregation characterizes whether an examination is predicted completely correctly, contains only adjacent-grade disagreements, or contains at least one maximum-distance severity disagreement.

This formulation differs from treating all condition–level targets as independent observations because it preserves the hierarchical organization of the original clinical annotation structure. It also makes possible the identification of cases in which apparently acceptable aggregate accuracy coexists with weak class-balanced or ordinal performance.

### 2.3. From Structured Prediction to Radiologist-Oriented AI

The potential clinical value of lumbar MRI AI depends not only on predictive performance but also on how predictions and uncertainty are communicated to the radiologist. Lim et al. demonstrated that deep-learning assistance can reduce lumbar MRI interpretation time while maintaining or improving agreement for stenosis assessment [22]. Such findings support the development of systems that function as structured decision-support tools rather than isolated image classifiers.

Automated reporting represents another emerging direction. Yeasin et al. introduced Auto-Rad, an end-to-end vision–language model for lumbar spine MRI report generation [19]. More broadly, recent work on automated radiology report generation has demonstrated the potential of vision–language architectures while simultaneously raising concerns regarding clinical fidelity, hallucination, and appropriate validation [23].

Quantitative patient-level representation is also gaining attention. SpineReport, for example, introduced automated three-dimensional morphometric analysis and subject-specific reporting for lumbar degeneration, extracting anatomical and signal-derived measurements associated with radiologist-assigned stenosis severity [24]. This direction supports the broader idea that lumbar degeneration can be represented as a structured patient profile rather than a collection of unrelated classification outputs.

The Beyond-Accuracy framework investigated in the present study provides an evaluation foundation for such future decision-support systems. Exact, ordinal, condition-specific, spinal-level, and patient-level analyses can be used during model development and retrospective validation to identify performance bottlenecks and potentially high-risk failure modes. In a future clinical deployment, these population-level validation measures could be combined with case-specific calibrated confidence or uncertainty estimates within a radiologist-oriented dashboard. Such deployment remains a future direction rather than a prospective clinical validation performed in the present study.

Overall, previous work has established the feasibility of automated lumbar MRI localization, degeneration grading, multiplanar modeling, quantitative measurement, and report generation. Comparatively less attention has been given to evaluating these systems simultaneously in terms of exact classification, ordinal severity distance, pathology-specific reliability, anatomical-level heterogeneity, and complete patient-level error structure. The present work addresses this evaluation gap through a common multi-level framework applied systematically across multiple architecture families and input representations.

## 3. Materials and Methods

### 3.1. Beyond-Accuracy Multi-Dimensional Evaluation Framework

Conventional evaluation of deep learning models for medical image classification commonly relies on aggregate performance measures, such as accuracy, precision, recall, and $F_{1}$-score. Although these metrics provide a useful indication of predictive performance, they do not fully characterize the clinical relevance of classification errors, particularly when the target categories represent ordered disease severity levels. In lumbar spine degeneration assessment, for example, misclassifying a severe abnormality as normal or mild is fundamentally different from confusing two adjacent severity categories. Furthermore, aggregate metrics may conceal substantial performance variation across pathological conditions, anatomical levels, and individual patients.

**Figure 1:**
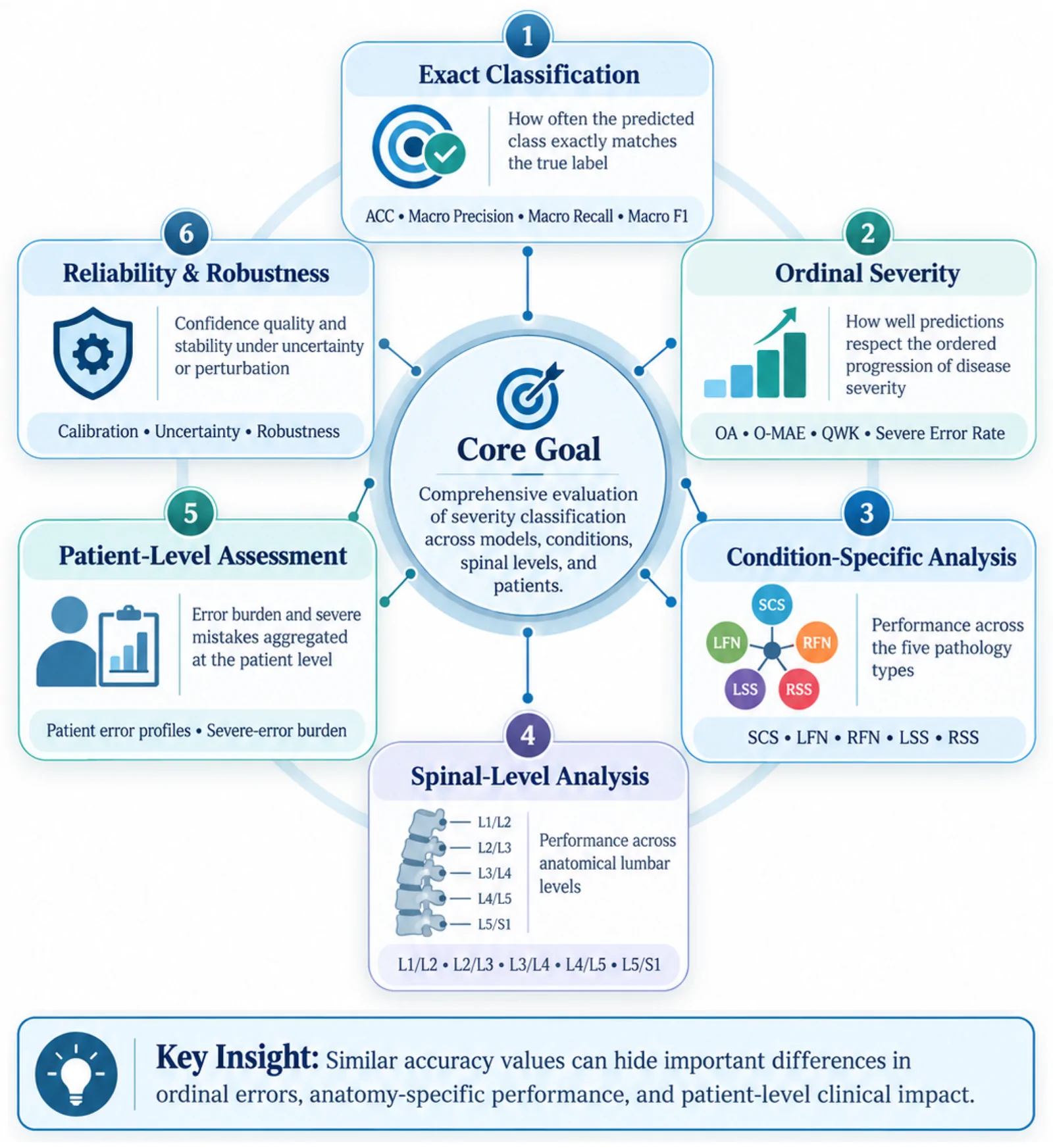
Overview of the proposed Beyond-Accuracy Multi-Dimensional Evaluation Framework for lumbar spine degeneration classification. Six complementary evaluation dimensions characterize exact predictive performance, ordinal severity consistency, condition-specific behavior, anatomical-level variability, patient-level error burden, and reliability and robustness. Two additional comparative dimensions examine imaging-plane information and spatial input dimensionality. The framework emphasizes that similar aggregate accuracy values may conceal clinically meaningful differences in error severity, anatomical performance, and patient-level outcomes.

To address these limitations, this study adopts a *Beyond-Accuracy Multi-Dimensional Evaluation Framework*, illustrated in Figure 1. The framework organizes model evaluation into six complementary dimensions: (i) exact classification, (ii) ordinal severity, (iii) condition-specific analysis, (iv) spinal-level analysis, (v) patient-level assessment, and (vi) reliability and robustness. In addition, two comparative dimensions examine the influence of imaging-plane information and spatial input dimensionality. Together, these perspectives support a more comprehensive interpretation of model performance than any individual aggregate metric.

(1) **Exact Classification:** The first dimension establishes the conventional predictive performance of each model using accuracy (ACC), macro-averaged precision, macro-averaged recall, and macro-averaged $F_{1}$-score. These measures quantify agreement between predicted and reference severity categories and provide a common baseline for comparing the investigated neural network architectures. Macro-averaged metrics are particularly relevant when the three severity classes exhibit unequal representation, as they assign equal importance to each class regardless of its prevalence.
(2) **Ordinal Severity Assessment:** The second dimension explicitly accounts for the ordered nature of lumbar degeneration severity, represented by normal/mild, moderate, and severe categories. In addition to ordinal accuracy (OA), the framework considers ordinal mean absolute error (O-MAE), quadratic weighted kappa (QWK), and severe ordinal error rate (SER). Unlike conventional accuracy, these measures distinguish between adjacent-class confusion and larger deviations across the severity scale. Consequently, they provide additional insight into the magnitude and clinical implications of classification errors, including the frequency of extreme misclassifications.
(3) **Condition-Specific Analysis:** The third dimension evaluates predictive performance independently for the five pathological conditions: spinal canal stenosis (SCS), left neural foraminal narrowing (LFN), right neural foraminal narrowing (RFN), left subarticular stenosis (LSS), and right subarticular stenosis (RSS). This analysis identifies condition-dependent differences in model effectiveness that may remain hidden within aggregated results. Such differences are important because the investigated conditions exhibit distinct anatomical characteristics, imaging appearances, and severity distributions.
(4) **Spinal-Level Analysis:** The fourth dimension examines classification behavior across the five lumbar intervertebral levels: L1/L2, L2/L3, L3/L4, L4/L5, and L5/S1. Stratifying model performance by anatomical level enables the identification of level-dependent error patterns and potential limitations in generalization across different regions of the lumbar spine. This perspective is particularly relevant because degeneration prevalence, anatomical morphology, and imaging characteristics may vary substantially between upper and lower lumbar levels.
(5) **Patient-Level Assessment:** The fifth dimension shifts the evaluation perspective from individual predictions to patient-level outcomes. Rather than treating all classification decisions as independent observations, this analysis aggregates prediction errors across the conditions and spinal levels associated with each patient. Patient-level error profiles and severe-error burden can reveal whether mistakes are distributed relatively uniformly or concentrated in a smaller subset of challenging cases. This perspective provides a more clinically meaningful characterization of prediction reliability at the examination level.
(6) **Reliability and Robustness:** The sixth dimension addresses the trustworthiness and stability of model predictions beyond their correctness. It encompasses confidence calibration, uncertainty characterization, and robustness to input perturbations or acquisition variability. Calibration assesses the agreement between predicted confidence and observed correctness, whereas uncertainty analysis investigates the ability of a model to distinguish reliable predictions from ambiguous cases. Robustness analysis further examines whether predictions remain stable under changes in input conditions. These aspects provide complementary criteria for evaluating the suitability of classification models for potential clinical decision-support applications.

#### Comparative Architectural Evaluation

Beyond the five primary evaluation levels, the framework compares conventional 2D representations with cross-level 2.5D-to-3D configurations constructed from five anatomically ordered lumbar-level ROIs (L1/L2–L5/S1). This comparison investigates whether cross-level anatomical context improves classification performance and ordinal severity assessment. Although the study processes sagittal and axial MRI acquisitions through condition-specific pipelines, explicit multiplanar fusion and geometry-aware volumetric reconstruction remain future research directions.

Importantly, the framework distinguishes between evaluation dimensions directly quantified in the present experiments and broader reliability or comparative analyses that may require additional experimental configurations. Thus, it serves both as an organizing structure for the reported results and as a foundation for subsequent investigations.

Overall, the proposed framework moves beyond the selection of a best-performing model based solely on aggregate accuracy. By jointly examining exact classification, ordinal error severity, condition-specific behavior, anatomical-level variability, patient-level error distribution, and prediction reliability, it facilitates a more informative and clinically contextualized comparison of deep learning architectures for lumbar spine degeneration assessment.

### 3.2. Dataset and ROI Preparation Overview

The experiments were conducted using the RSNA 2024 Lumbar Spine Degenerative Classification (LumbarDISC) dataset, which contains lumbar MRI examinations acquired using multiple MRI sequences and anatomical imaging planes. The classification task considers five degenerative conditions: spinal canal stenosis (SCS), left neural foraminal narrowing (LFN), right neural foraminal narrowing (RFN), left subarticular stenosis (LSS), and right subarticular stenosis (RSS). Each condition is assessed at five lumbar levels, L1/L2, L2/L3, L3/L4, L4/L5, and L5/S1, using the three ordinal severity categories Normal/Mild, Moderate, and Severe.

For supervised model development, the coordinate-linked subset of the dataset was used because the provided annotations associate selected MRI instances with a degenerative condition, lumbar level, and expert-provided spatial coordinate. These coordinates were used to construct anatomically localized regions of interest (ROIs), thereby defining an expert-localized classification setting rather than a fully automatic localization pipeline.

For an expert-provided coordinate (x,y), a square ROI spanning 12.5% of the native image width and height was centered on the annotated location. Crop boundaries were clipped to the valid image extent, and the resulting ROI was subsequently standardized to a 64×64 pixel model input. This proportional strategy maintains a comparable local anatomical field of view across heterogeneous native DICOM matrix sizes while separating anatomical localization from computational input standardization.

Detailed characterization of the LumbarDISC hierarchy, annotation availability, severity distributions, annotation multiplicity, cross-condition DICOM reuse, native image-resolution distributions, coordinate organization, and qualitative validation of the ROI extraction procedure is provided in Appendix A.

### 3.3. Study-Level Dataset Partitioning

To ensure a reproducible evaluation protocol and prevent information leakage between related MRI instances, the usable annotated data were partitioned at the *study level* rather than by individual DICOM image. All images, coordinate annotations, and derived condition–level samples originating from the same MRI study were therefore assigned to a single experimental subset.

For each condition-specific dataset c∈{SCS,LFN,RFN,LSS,RSS}, the target partitioning ratio was defined as

(1) $$D^{\left(c\right)}=D_{\mathrm{train}}^{\left(c\right)}\cup D_{\mathrm{val}}^{\left(c\right)}\cup D_{\mathrm{test}}^{\left(c\right)},$$

with

$$\left|D_{\mathrm{train}}^{\left(c\right)}\right|:\left|D_{\mathrm{val}}^{\left(c\right)}\right|:\left|D_{\mathrm{test}}^{\left(c\right)}\right|\approx 70\%:15\%:15\%.$$

The corresponding study sets were mutually disjoint,

$$S_{\mathrm{train}}\cap S_{\mathrm{val}}=S_{\mathrm{train}}\cap S_{\mathrm{test}}=S_{\mathrm{val}}\cap S_{\mathrm{test}}=⌀,$$

ensuring that no MRI examination contributed samples to more than one partition.

**Figure 2:**
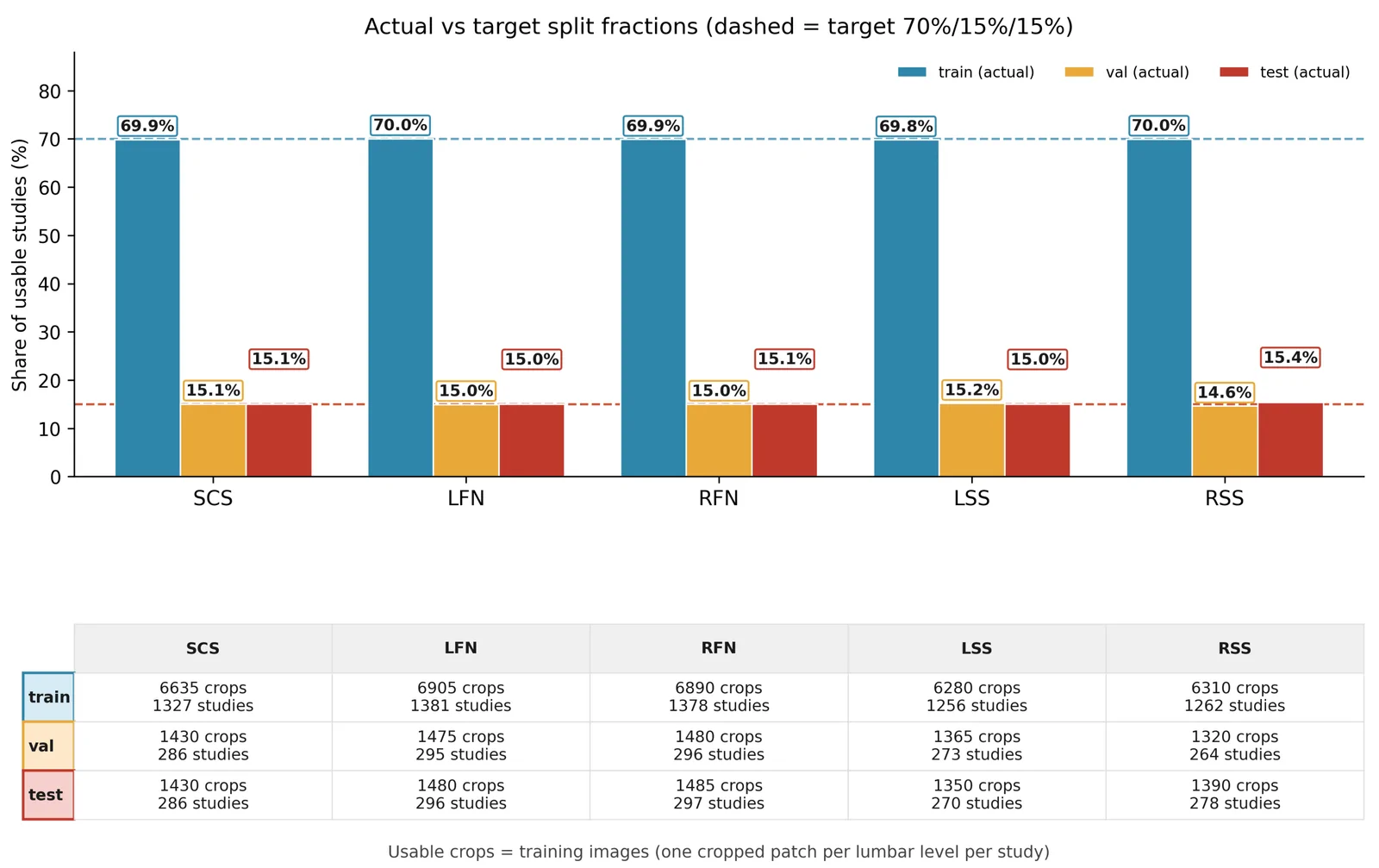
Realized study-level train, validation, and test partitions for the five condition-specific datasets. Dashed reference lines indicate the target 70%/15%/15% split, while the bars show the actual proportions obtained after enforcing study-level separation. The lower table reports the corresponding numbers of usable condition-specific samples and unique studies for spinal canal stenosis (SCS), left and right neural foraminal narrowing (LFN and RFN), and left and right subarticular stenosis (LSS and RSS). Small deviations from the target fractions result from preserving complete studies within a single partition.

Figure 2 compares the target fractions with the realized proportions for the five condition-specific pipelines. The achieved training fractions remain close to 70%, while validation and test fractions remain close to 15%. Small deviations from the nominal 70/15/15 ratio arise because complete studies, rather than individual image crops, are assigned to each subset.

Preserving study integrity was prioritized over enforcing exact image-level split proportions, as each MRI examination may contribute multiple condition–level samples. Study-level partitioning was performed before ROI extraction to prevent correlated images and derived samples from appearing across different subsets. The resulting partitions were used for the five condition-specific modeling pipelines. Further preprocessing details are provided in Appendix A.

The training partition (70%) was used for model optimization, while the validation partition (15%) supported early stopping, architecture selection, and hyperparameter optimization using validation accuracy (ACC). The held-out test partition (15%) was reserved for independent evaluation and excluded from model development. Thus, validation results represent development-stage performance, whereas independent test results are required to assess model generalization. For the five repeated confirmatory runs, the study-level training, validation, and test partitions were regenerated using distinct random split seeds, maintaining approximately the same 70/15/15 proportions and ensuring mutually disjoint studies within each run.

### 3.4. Vision Transformer Pipeline

**Figure 3:**
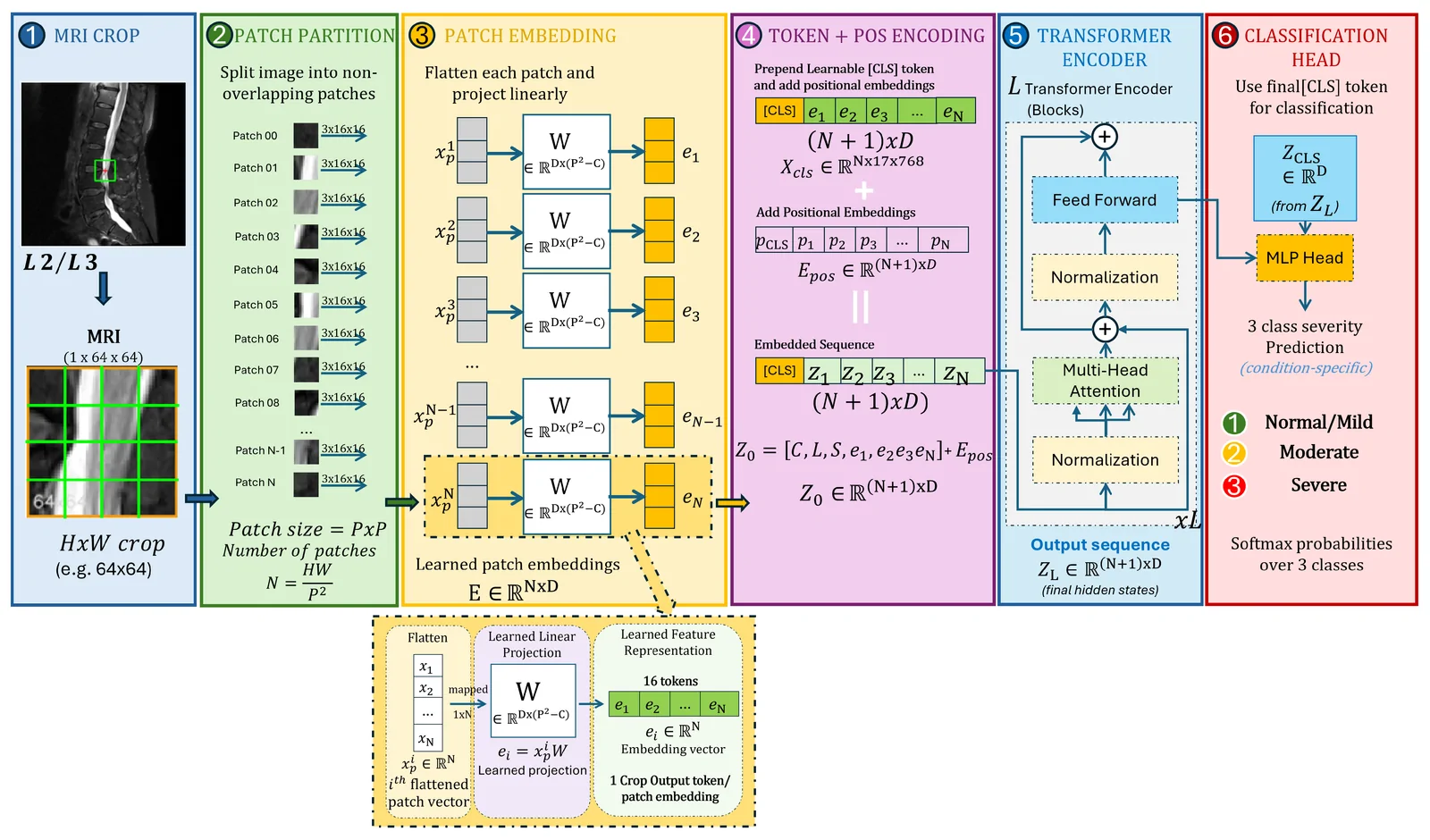
Condition-specific Vision Transformer pipeline for lumbar MRI severity classification. A localized grayscale MRI crop is transformed into patch embeddings, combined with a classification token and positional embeddings, and processed by Transformer encoder blocks. The final classification-token representation is mapped to three severity categories: Normal/Mild, Moderate, and Severe.

Figure 3 illustrates the Vision Transformer (ViT) pipeline employed for condition-specific lumbar MRI severity classification. The architecture processes localized MRI crops through six principal stages: (i) crop preprocessing, (ii) patch partitioning, (iii) patch embedding, (iv) classification-token and positional encoding, (v) Transformer encoding, and (vi) severity classification.

Each input consists of a 64×64 grayscale MRI crop centered on an annotated lumbar spinal level. The grayscale channel is replicated three times to ensure compatibility with ImageNet-pretrained ViT architectures. The resulting image is partitioned into non-overlapping patches, which are flattened and projected into a learned embedding space. For the /16 and /32 configurations, this produces sixteen and four image patches, respectively.

A learnable classification token ([CLS]) is prepended to the patch embeddings, and positional embeddings are incorporated to preserve spatial information. The resulting sequence is processed through stacked Transformer encoder blocks comprising multi-head self-attention and feed-forward networks. Self-attention enables the model to capture contextual relationships among anatomical regions within the localized MRI crop.

The final [CLS] representation is passed to a classification head that predicts one of three ordered severity categories: Normal/Mild, Moderate, or Severe. The same processing pipeline is applied independently to the five investigated lumbar conditions (SCS, LFN, RFN, LSS, and RSS). The ViT variants differ in patch size, embedding dimensionality, and encoder capacity, while retaining the same overall architecture.

The detailed mathematical formulation, intermediate tensor dimensions, and classification operations are provided in Appendix B.

### 3.5. Cross-Level 2.5D-to-3D Pipeline

In addition to the specialized 2D models, this study investigates a cross-level 2.5D-to-3D representation to determine whether jointly modeling the five anatomically ordered lumbar levels provides complementary information for severity classification. Unlike conventional 2.5D approaches based on adjacent MRI slices, the proposed representation stacks localized regions of interest (ROIs) from five distinct lumbar levels in cranial-to-caudal order: L1/L2, L2/L3, L3/L4, L4/L5, and L5/S1.

This representation was selected as a computationally tractable exploratory comparison that uses the available coordinate-linked ROIs without requiring volumetric reconstruction or additional anatomical annotations. Rather than proposing an optimal 3D imaging strategy, the objective was to investigate whether structured cross-level information could benefit severity classification and to evaluate its behavior under the same Beyond-Accuracy framework applied to the specialized 2D models.

The pipeline uses the same preprocessed 64×64 grayscale ROI crops as the 2D models, extracted around annotated spinal-level coordinates using a centered crop covering 12.5% of the source image dimension. Each crop is replicated across three channels, and the five ROIs are arranged into a single input tensor:

(2) $$X_{2.5D}\in R^{B\times 3\times 5\times 64\times 64},$$

where *B* denotes the batch size and the remaining dimensions correspond to channels, lumbar levels, height, and width, respectively. Only study–condition samples containing all five annotated levels are included; incomplete stacks are excluded without padding.

The resulting representation is processed by a shared 3D encoder, followed by five level-specific classification heads. Each head predicts one of three ordered severity categories: Normal/Mild, Moderate, or Severe. Four architecture families are evaluated using this formulation: ViT-3D, MaxViT-3D, ConvNeXt-3D, and EfficientNet-3D.

The 2.5D-to-3D models follow the same general training protocol as the 2D experiments, with a reduced batch size of 8 to accommodate increased memory requirements. Training uses a maximum of 50 epochs, early stopping with a patience of five epochs, cosine learning-rate scheduling, and Automatic Mixed Precision (AMP).

This formulation enables cross-level feature interaction while preserving independent severity predictions for each anatomical level. The comparison with specialized 2D models therefore examines whether additional cranial–caudal anatomical context improves classification performance. Importantly, the stacked depth dimension represents distinct lumbar levels rather than physically contiguous DICOM slices. Consequently, this representation does not constitute a geometry-preserving volumetric MRI reconstruction or explicit sagittal–axial feature fusion, although both imaging planes are used across the condition-specific pipelines.

### 3.6. GPU Parallelization Strategy

**Figure 4:**
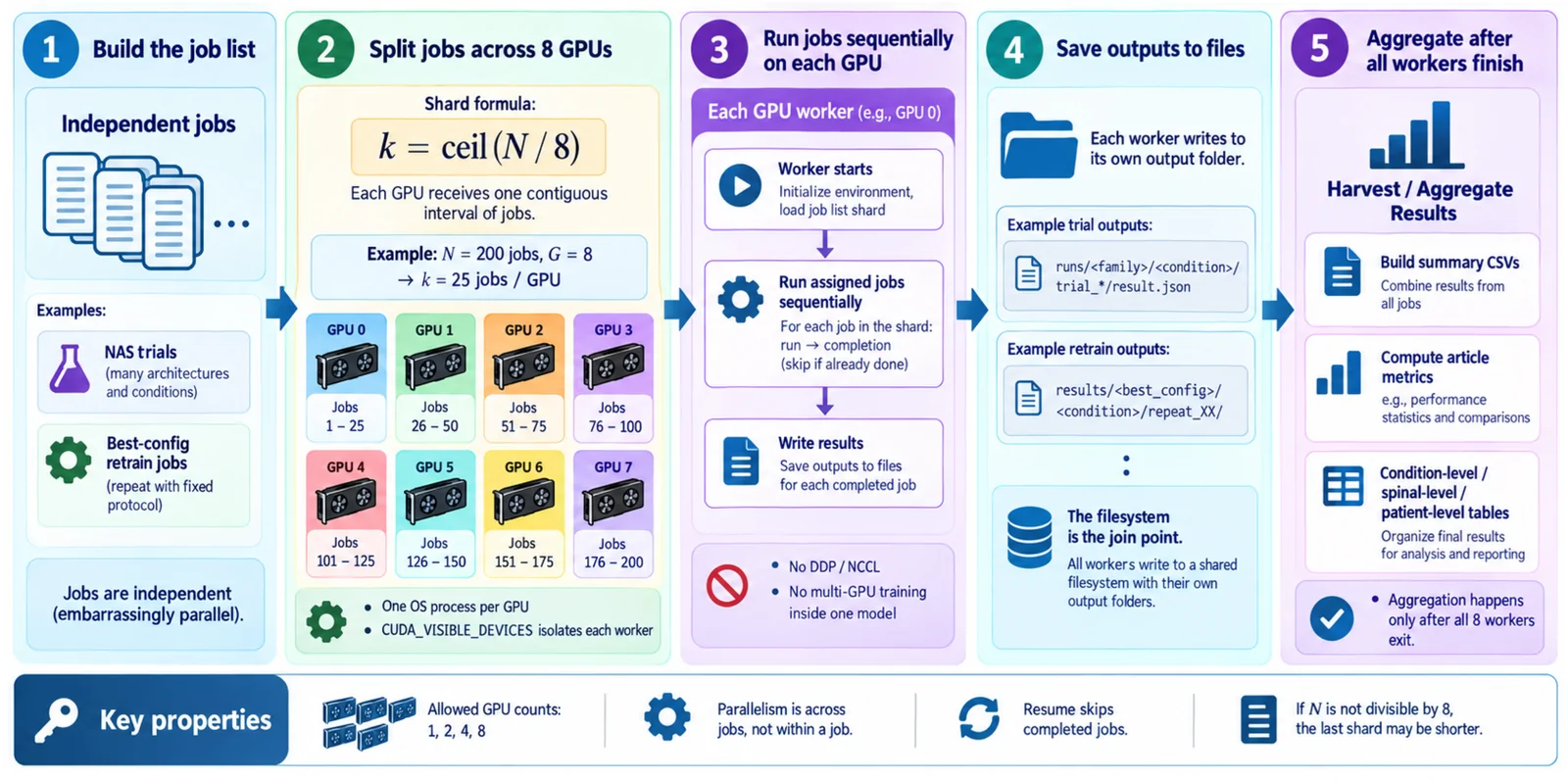
GPU parallelization strategy for NAS and best-configuration retraining, illustrated using an eight-GPU configuration. Independent training jobs are divided into contiguous shards and distributed across GPU workers, with each worker executing its assigned jobs sequentially. Intermediate results are saved independently to the filesystem, enabling interrupted experiments to resume without repeating completed jobs. Final model metrics and condition-specific, spinal-level, and patient-level results are aggregated only after all GPU workers finish. The illustrated example distributes 200 retraining jobs evenly across eight GPUs, assigning 25 jobs to each device.

Neural architecture search (NAS) and confirmatory best-configuration retraining were parallelized across independent training jobs rather than through intra-model multi-GPU data parallelism. As illustrated in Figure 4, the proposed execution strategy follows five stages: (i) independent job generation, (ii) workload partitioning across GPUs, (iii) sequential execution within each GPU, (iv) independent result storage, and (v) final aggregation and post-processing.

Each GPU worker operated as an independent process, isolated through CUDA_VISIBLE _DEVICES, thereby preserving job-specific batch sizes, Automatic Mixed Precision (AMP) settings, CUDA contexts, and random seed configurations. The implementation supports G∈{1,2,4,8} GPUs. For a workload containing *N* independent jobs, the number of jobs assigned to each GPU shard was determined as where *k* denotes the maximum shard size and *G* represents the number of active GPUs. As illustrated in Figure 4, a workload of 200 retraining jobs distributed across eight GPUs produces 25 jobs per device.

(3) $$k=\left\lceil \frac{N}{G}\right\rceil ,$$

For NAS, parallelism was applied within one architecture family and one lumbar condition at a time. Each condition occupied all available GPUs until its assigned trials completed, after which the next condition was processed. The same sharding mechanism was reused for best-configuration retraining, where completed runs were excluded before workload partitioning to support resumable execution.

Each worker independently stored its training artifacts and evaluation results in dedicated filesystem directories. After all workers completed, the outputs were consolidated into summary files for article-level metric computation. Condition-specific, spinal-level, and patient-level analyses were subsequently performed as post hoc CPU-based operations, without additional GPU parallelization.

### 3.7. Neural Architecture Search Strategy

To identify competitive model configurations for lumbar degeneration severity classification, we performed an isolated-family neural architecture search (NAS) across four architecture families: Vision Transformer (ViT), MaxViT, ConvNeXt, and EfficientNet. Each family was evaluated independently under both the 2D and cross-level 2.5D-to-3D input representations described previously.

A single NAS trial corresponds to one discrete hyperparameter configuration trained for one degenerative condition. The five target conditions, SCS, LFN, RFN, LSS, and RSS, were searched independently using the same family-specific configuration space. Thus, model selection was performed separately for each condition rather than by optimizing one global configuration across all pathologies.

All searches used full Cartesian grid enumeration rather than stochastic or Bayesian optimization. Trials were trained for at most 50 epochs with early stopping using a patience of five epochs, cosine learning-rate scheduling, and Automatic Mixed Precision (AMP). The backbone was not frozen during search. The reported 2D runs used a batch size of 128, while the 2.5D-to-3D runs used a batch size of 8 because of the higher memory requirements of the 3D encoders. The 2D models were initialized from ImageNet-pretrained weights. Among the 2.5D-to-3D models, ImageNet initialization was retained only for MaxViT-3D, whereas ViT-3D, ConvNeXt-3D, and EfficientNet-3D were trained from scratch.

Following NAS-based configuration selection, the selected configurations were retrained in five confirmatory runs using distinct study-level split seeds and training random seeds. Each run independently generated its training, validation, and test partitions, maintaining approximately the 70/15/15 proportions described in Section 3.3. Performance was summarized as the mean ± standard deviation across the completed runs.

#### 3.7.1. 2D Architecture Families and Search Spaces

The 2D neural architecture search evaluated four model families using the same centered 64×64 ROI representation: ViT, MaxViT, ConvNeXt, and EfficientNet. Model selection was performed independently for each of the five degenerative conditions, so every NAS trial corresponded to one discrete architecture–hyperparameter configuration trained for one condition.

The Transformer-based families, ViT and MaxViT, used two optimization blocks: a larger AdamW grid varying learning rate, weight decay, classification-head depth, and hidden width, and a smaller SGD grid with a fixed head geometry. This resulted in 2304 trials per condition for ViT and 1152 trials per condition for MaxViT. ConvNeXt and EfficientNet used pruned AdamW grids that varied learning rate, weight decay, head depth, hidden width, dropout, and activation function, resulting in 144 trials per condition for each family.

Across the four 2D architecture families, the complete search space therefore contained

2304+1152+144+144=3744

trials per degenerative condition. All 2D searches used ImageNet initialization, a batch size of 128, cosine learning-rate scheduling, Automatic Mixed Precision (AMP), a maximum of 50 epochs, and early stopping with patience 5.

To keep the main methodology concise, the complete hyperparameter grids, including all learning-rate, weight-decay, optimizer, classification-head, dropout, and activation choices, are reported in Appendix C, Table A2.

#### 3.7.2. 2.5D-to-3D Architecture Families and Search Spaces

The cross-level 2.5D-to-3D neural architecture search evaluated four model families—ViT-3D, MaxViT-3D, ConvNeXt-3D, and EfficientNet-3D—using the same five localized 64×64 ROI crops as the 2D pipeline, reorganized along a depth dimension corresponding to the ordered lumbar levels L1/L2–L5/S1. Thus, the NAS operated on the 3d_level_stack representation rather than on a reconstructed patient-space MRI volume.

The Transformer-based 3D families retained the two-block search strategy used for the 2D Transformer models, combining a larger AdamW grid with a smaller SGD grid. The learning-rate ranges were shifted downward to accommodate the smaller batch size and the less stable optimization regime of the 3D models. This resulted in 2304 trials per condition for ViT-3D and 1152 trials per condition for MaxViT-3D.

ConvNeXt-3D and EfficientNet-3D each used two model variants and a combined AdamW/SGD grid. The AdamW block contributed 144 configurations per variant and the SGD block an additional 48, resulting in 192 configurations per variant and 384 trials per family and condition.

Across the four 2.5D-to-3D architecture families, the total search space therefore contained

2304+1152+384+384=4224

trials per degenerative condition. All searches used the centered 64×64 ROI representation, a batch size of 8, cosine learning-rate scheduling, Automatic Mixed Precision (AMP), a maximum of 50 epochs, and early stopping with patience 5. ImageNet initialization was used only for MaxViT-3D, while ViT-3D, ConvNeXt-3D, and EfficientNet-3D were trained from scratch. The complete 2.5D-to-3D hyperparameter grids are provided in Appendix C, Table A3.

### 3.8. Performance Metrics

#### 3.8.1. Exact Classification Metrics

Exact classification performance is evaluated using Accuracy (ACC), Precision, Recall, and $F_{1}$-score. These conventional metrics provide the baseline assessment of strict agreement between the predicted and ground-truth severity classes and are used as the reference point for the proposed beyond-accuracy analysis.

For a given class, let TP, TN, FP, and FN denote the numbers of true positives, true negatives, false positives, and false negatives, respectively. Accuracy is defined as

(4) $$ACC=\frac{TP+TN}{TP+TN+FP+FN}.$$

Precision measures the proportion of predicted positive cases that are correctly classified:

(5) $$Precision=\frac{TP}{TP+FP}.$$

Recall, also referred to as sensitivity, measures the proportion of actual positive cases that are correctly identified:

(6) $$Recall=\frac{TP}{TP+FN}.$$

The $F_{1}$-score is the harmonic mean of Precision and Recall:

(7) $$F1=2\cdot \frac{Precision\cdot Recall}{Precision+Recall}.$$

Because the present task consists of three severity classes (*Normal/Mild*, *Moderate*, and *Severe*), Precision, Recall, and $F_{1}$-score are computed independently for each class and then macro-averaged:

(8) $$\text{Macro-M}=\frac{1}{C}\sum_{c=1}^{C}M_{c},$$

where $M_{c}$ represents the class-specific metric and C=3 is the number of severity classes. Macro averaging gives equal importance to each class and is therefore less influenced by class imbalance than micro-averaged measures.

These metrics quantify exact class agreement only. Consequently, a prediction of *Normal/Mild* instead of *Moderate* is penalized in the same manner as a prediction of *Normal/Mild* instead of *Severe*. This limitation motivates the ordinal and multi-level evaluation dimensions introduced in the following subsections.

#### 3.8.2. Ordinal Severity Metrics

The lumbar degeneration labels constitute an inherently ordinal classification problem, following the clinically meaningful progression

(9) Normal/Mild=0<Moderate=1<Severe=2.

Consequently, not all classification errors have the same severity. A Normal/Mild prediction classified as Moderate represents an adjacent one-grade error, whereas a Normal/Mild prediction classified as Severe spans the complete severity scale. Conventional accuracy treats both predictions as equally incorrect and therefore does not capture the magnitude of the disagreement.

**Figure 5:**
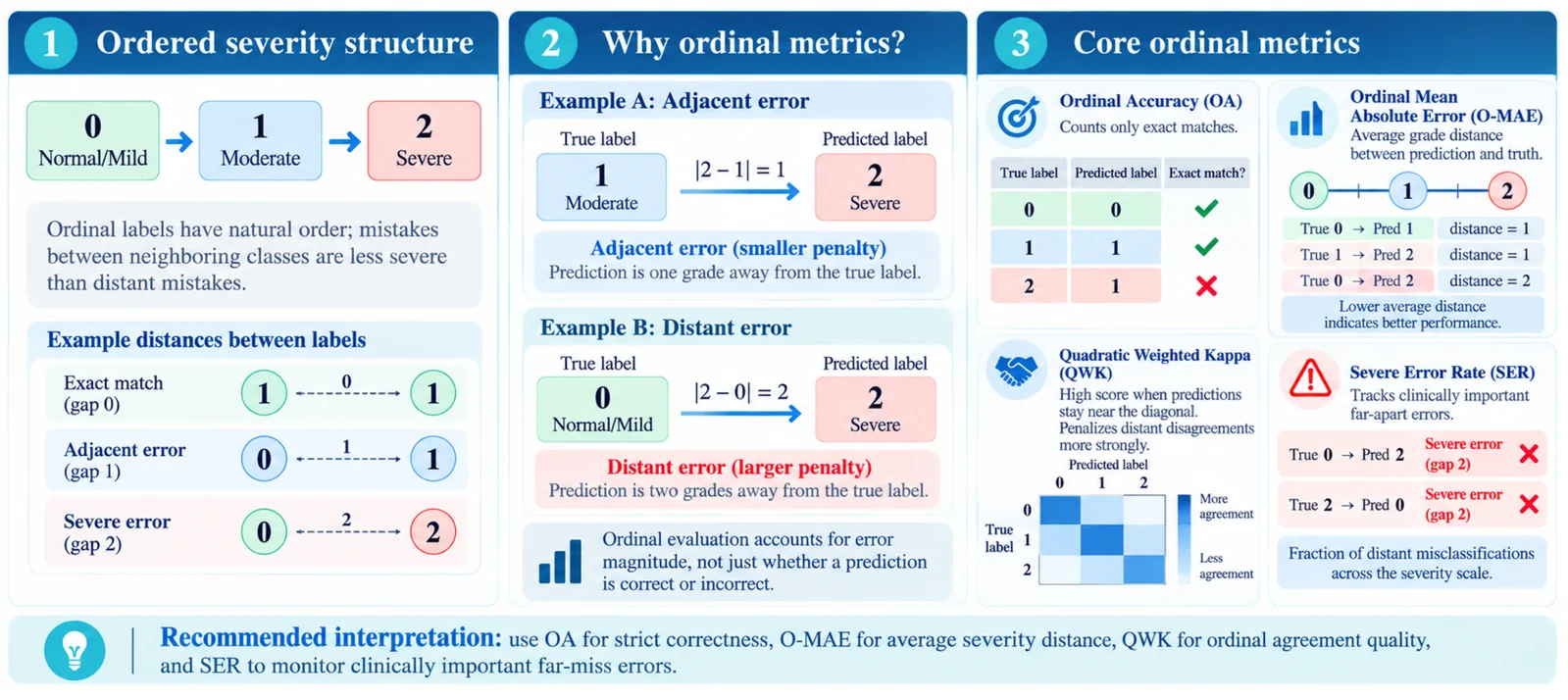
Ordinal severity evaluation for the three lumbar degeneration grades. Adjacent errors span one severity level, whereas direct Normal/Mild–Severe disagreements span two levels and represent the maximum ordinal error. OA and O-MAE quantify severity distance, QWK evaluates weighted ordinal agreement, and SER explicitly measures maximum-distance errors.

Figure 5 summarizes this principle and the four ordinal metrics adopted in the proposed Beyond-Accuracy framework: Ordinal Accuracy (OA), Ordinal Mean Absolute Error (O-MAE), Quadratic Weighted Kappa (QWK), and Severe Ordinal Error Rate (SER).

For a reference severity label $y_{i}$ and predicted label ${\hat{y}}_{i}$, the ordinal distance is defined as

(10) $$d_{i}=\left|y_{i}-{\hat{y}}_{i}\right|,$$

where $d_{i}\in \left\{0,1,2\right\}$. Thus, $d_{i}=0$ represents an exact prediction, $d_{i}=1$ an adjacent-grade disagreement, and $d_{i}=2$ a maximum-distance severity error.

**Ordinal Accuracy (OA).** OA measures prediction quality while assigning partial credit according to ordinal proximity:

(11) $$OA=1-\frac{1}{N(C-1)}\sum_{i=1}^{N}\left|y_{i}-{\hat{y}}_{i}\right|,$$

where *N* is the number of evaluated predictions and C=3 is the number of severity classes. In the present setting, an exact prediction contributes 1.0, an adjacent error contributes 0.5, and a maximum-distance error contributes 0. Higher OA therefore indicates stronger ordinal agreement.

**Ordinal Mean Absolute Error (O-MAE).** The average severity distance between predictions and reference labels is measured as

(12) $$\text{O-MAE}=\frac{1}{N}\sum_{i=1}^{N}\left|y_{i}-{\hat{y}}_{i}\right|.$$

Lower O-MAE values indicate better performance, with O-MAE=0 corresponding to perfect prediction. For the present three-class formulation, OA and O-MAE are directly related through

(13) $$OA=1-\frac{\text{O-MAE}}{2}.$$

**Quadratic Weighted Kappa (QWK).** QWK evaluates agreement between reference and predicted severity grades while penalizing larger ordinal disagreements more strongly. The quadratic disagreement weight between classes *i* and *j* is

(14) $$w_{ij}=\frac{{\left(i-j\right)}^{2}}{{\left(C-1\right)}^{2}},$$

and the resulting weighted agreement is

(15) $$QWK=1-\frac{\sum_{i=0}^{C-1}\sum_{j=0}^{C-1}w_{ij}O_{ij}}{\sum_{i=0}^{C-1}\sum_{j=0}^{C-1}w_{ij}E_{ij}},$$

where $O_{ij}$ and $E_{ij}$ denote the observed and expected class agreement matrices, respectively. Values approaching 1 indicate stronger ordinal agreement.

##### Severe Ordinal Error Rate (SER)

SER specifically quantifies the most severe classification failures:

(16) $$SER=\frac{1}{N}\sum_{i=1}^{N}I\left(|y_{i}-{\hat{y}}_{i}|\;=2\right),$$

where I(·) is the indicator function. For the three-class severity scale, SER therefore represents the proportion of direct Normal/Mild ↔ Severe errors. Lower SER is preferred, with SER=0 indicating the absence of maximum-distance severity misclassifications.

These four measures provide complementary interpretations of ordinal model behavior. OA summarizes overall proximity to the correct severity grade, O-MAE expresses the average magnitude of prediction errors, QWK measures chance-corrected ordinal agreement, and SER isolates the most severe Normal/Mild–Severe confusions. They are computed independently for each degenerative condition and subsequently macro-averaged across SCS, LFN, RFN, LSS, and RSS for pipeline-level comparison. The same ordinal formulation is also reused in the subsequent condition-, spinal-, and patient-level evaluation stages.

#### 3.8.3. Condition-Specific Evaluation

Because the five lumbar degenerative conditions differ in anatomical location, imaging appearance, and severity-class distribution, aggregate pipeline performance may obscure substantial condition-dependent variation. The third dimension of the Beyond-Accuracy framework therefore evaluates each degenerative condition independently: Spinal Canal Stenosis (SCS), Left Neural Foraminal Narrowing (LFN), Right Neural Foraminal Narrowing (RFN), Left Subarticular Stenosis (LSS), and Right Subarticular Stenosis (RSS).

**Figure 6:**
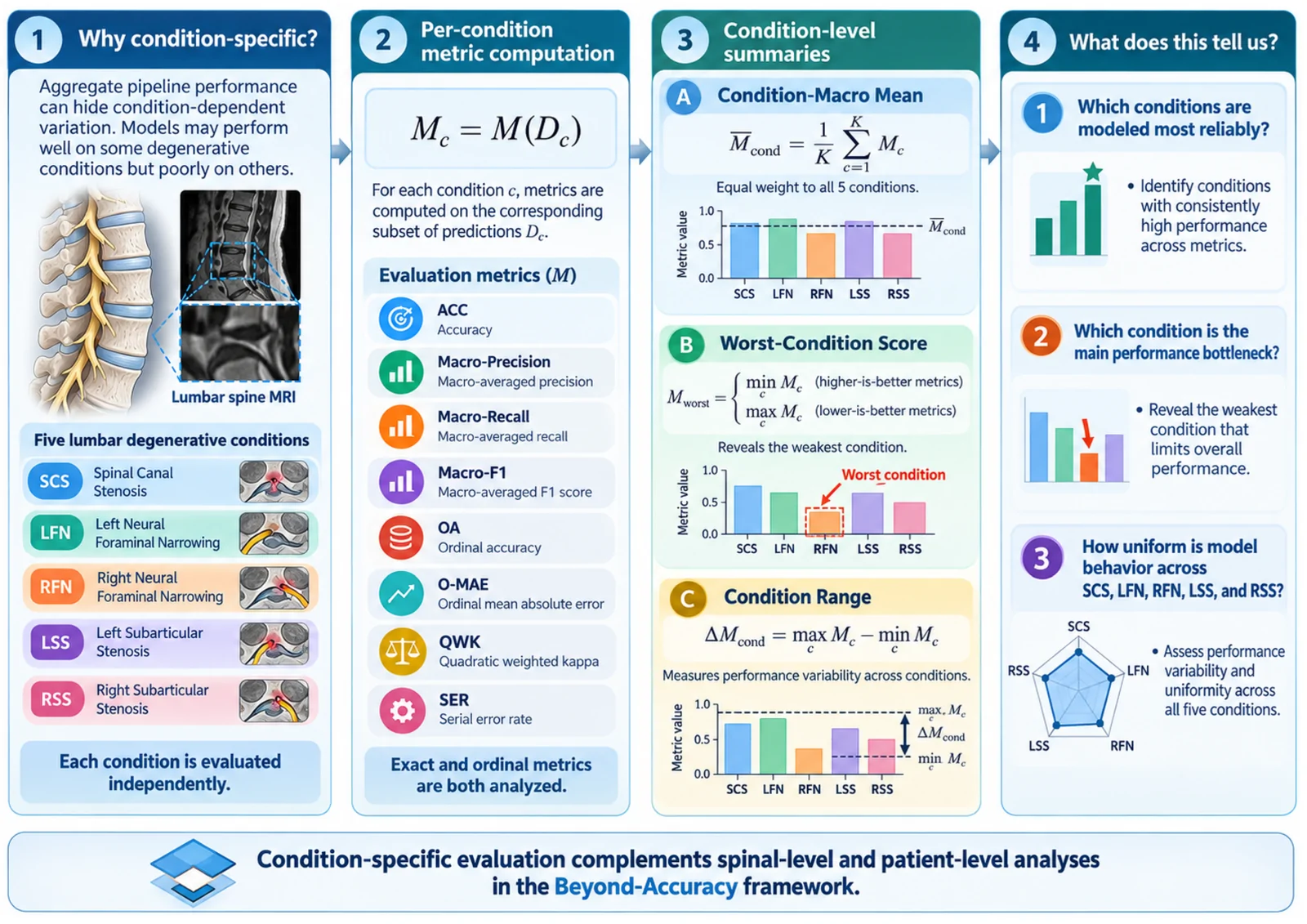
Condition-specific evaluation within the Beyond-Accuracy framework. Metrics are evaluated independently for SCS, LFN, RFN, LSS, and RSS. The resulting condition profiles are summarized using the macro-condition mean, worst-condition score, and condition range. Star-profile visualization provides an intuitive representation of performance uniformity across the five degenerative conditions.

Figure 6 summarizes the proposed condition-specific evaluation strategy. Rather than introducing an additional prediction task, this stage decomposes the performance of the already selected condition-specific classifiers and determines whether a pipeline behaves consistently across the five degenerative findings or whether its aggregate performance is driven primarily by a subset of easier conditions.

For each condition *c*, the corresponding subset of predictions is denoted by $D_{c}$. A metric *M* is evaluated independently on each condition-specific subset as

(17) $$M_{c}=M\left(D_{c}\right),$$

where *M* may represent a conventional classification metric (ACC, Macro-Precision, Macro-Recall, or Macro-F1) or an ordinal severity metric (OA, O-MAE, QWK, or SER). The resulting five values form a condition-performance profile for a given architecture and input representation.

For an architecture-level condition summary, equal weight is assigned to the five degenerative conditions through the macro-condition mean

(18) $${\bar{M}}_{\mathrm{cond}}=\frac{1}{K}\sum_{c=1}^{K}M_{c},\;K=5.$$

This formulation prevents conditions containing a larger number of valid observations from disproportionately determining the summary result and therefore provides an equal-weight comparison across SCS, LFN, RFN, LSS, and RSS.

In addition to the condition-macro mean, the weakest condition is explicitly identified. For higher-is-better metrics such as ACC, Macro-F1, OA, and QWK,

(19) $$M_{\mathrm{worst}}=\min_{c}M_{c},$$

whereas for lower-is-better metrics such as O-MAE and SER,

(20) $$M_{\mathrm{worst}}=\max_{c}M_{c}.$$

The corresponding condition identifies the principal pathology-specific performance bottleneck of the evaluated pipeline.

To quantify the degree of performance variation across conditions, the condition range is defined as

(21) $$\Delta M_{\mathrm{cond}}=\max_{c}M_{c}-\min_{c}M_{c}.$$

A smaller $\Delta M_{\mathrm{cond}}$ indicates more uniform performance across the five degenerative findings, whereas a larger range indicates stronger condition dependence. The standard deviation of the five condition-specific values is additionally reported as a complementary measure of variability.

The resulting five-dimensional condition profile can also be visualized using a star or radar representation, as illustrated in Figure 6. Each axis corresponds to one degenerative condition, allowing both absolute performance and cross-condition uniformity to be inspected simultaneously. A relatively symmetric profile indicates similar behavior across SCS, LFN, RFN, LSS, and RSS, whereas an irregular profile highlights condition-specific weaknesses that may be hidden by a single aggregate score.

The condition-specific evaluation therefore addresses three complementary questions: which degenerative conditions are modeled most reliably, which condition constitutes the principal performance bottleneck, and how uniformly the model behaves across the five findings. This analysis is subsequently complemented by spinal-level and patient-level evaluations, which further localize model behavior within the anatomical and patient-specific structure of the lumbar MRI assessment task.

#### 3.8.4. Spinal-Level Evaluation

Lumbar degeneration is assessed across five ordered intervertebral levels: L1/L2, L2/L3, L3/L4, L4/L5, and L5/S1. Because model performance may vary substantially across anatomical levels, the fourth dimension of the Beyond-Accuracy framework evaluates predictions independently at each spinal level rather than relying only on condition- or patient-level aggregates. This spinal-level view is important because a model with good global performance may still exhibit localized weaknesses at specific lumbar levels.

**Figure 7:**
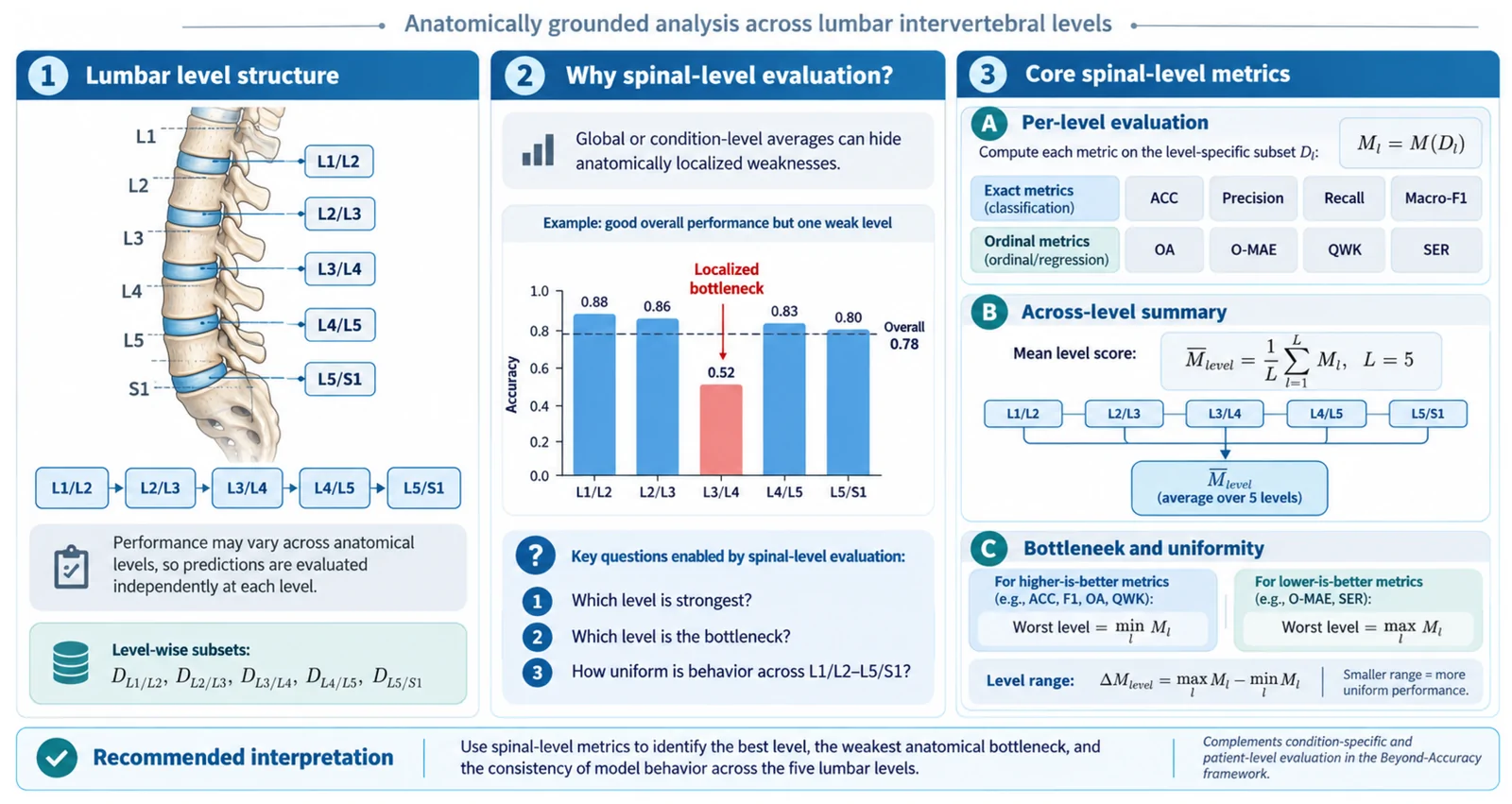
Overview of the spinal-level evaluation dimension in the proposed Beyond-Accuracy framework. Predictions are analyzed independently across the five lumbar intervertebral levels (L1/L2 to L5/S1) in order to identify level-specific performance differences. For each level, both exact and ordinal metrics can be computed, and the resulting level-wise profile allows the identification of the strongest and weakest anatomical regions of model performance. This analysis complements condition-specific evaluation by adding explicit anatomical localization along the lumbar spine.

Figure 7 provides a visual overview of the spinal-level evaluation concept used in this study. The figure emphasizes three complementary ideas: (1) each lumbar level is evaluated separately, (2) level-wise exact and ordinal metrics can be computed independently, and (3) the resulting profile can reveal the anatomical location of the strongest and weakest model behavior. In this way, spinal-level analysis extends the condition-specific perspective by explicitly localizing model performance along the cranio-caudal structure of the lumbar spine.

For each spinal level *l*, let $D_{l}$ denote the subset of predictions associated with that level. Any evaluation metric *M* can then be computed independently as

(22) $$M_{l}=M\left(D_{l}\right),$$

where *M* may represent ACC, Macro-F1, OA, O-MAE, QWK, or SER. This decomposition enables direct comparison of model behavior between upper and lower lumbar levels and helps identify anatomically localized performance degradation.

For ordinal evaluation, spinal-level ordinal accuracy is computed as

(23) $$OA_{l}=1-\frac{1}{N_{l}\left(C-1\right)}\sum_{i=1}^{N_{l}}\left|y_{i,l}-{\hat{y}}_{i,l}\right|,$$

where $N_{l}$ is the number of evaluated samples at spinal level *l*, $y_{i,l}$ is the reference severity grade, and ${\hat{y}}_{i,l}$ is the corresponding model prediction. As in the global ordinal formulation, C=3, such that exact predictions receive full credit, adjacent-grade errors receive partial credit, and maximum-distance errors receive no credit.

To summarize performance across the lumbar spine, the mean spinal-level metric is defined as

(24) $${\bar{M}}_{\mathrm{level}}=\frac{1}{L}\sum_{l=1}^{L}M_{l},$$

where L=5 corresponds to the five evaluated intervertebral levels.

In addition to the mean, the worst-performing spinal level is explicitly reported as

(25) $$M_{\mathrm{worst}}=\min_{l\in \{1,\ldots ,L\}}M_{l},$$

for metrics where higher values indicate better performance, such as ACC, Macro-F1, OA, and QWK. For error-based measures such as O-MAE and SER, the corresponding worst level is identified using the maximum value.

This analysis is important because similar global performance may conceal localized weaknesses at specific anatomical levels. For example, a model may perform consistently at L1/L2–L3/L4 while showing reduced reliability at L4/L5 or L5/S1. Reporting level-wise exact and ordinal metrics therefore provides a more anatomically grounded characterization of model behavior and serves as a natural bridge between condition-specific and patient-level evaluation.

#### 3.8.5. Patient-Level Evaluation

The fifth dimension of the Beyond-Accuracy framework evaluates model behavior at the patient level. Each patient may contribute multiple predictions across the five degenerative conditions and five lumbar intervertebral levels. Consequently, sample-level, condition-specific, and spinal-level metrics do not fully characterize whether prediction errors are dispersed across many patients or become concentrated within individual examinations. Patient-level evaluation therefore aggregates all valid condition–level predictions belonging to the same subject.

**Figure 8:**
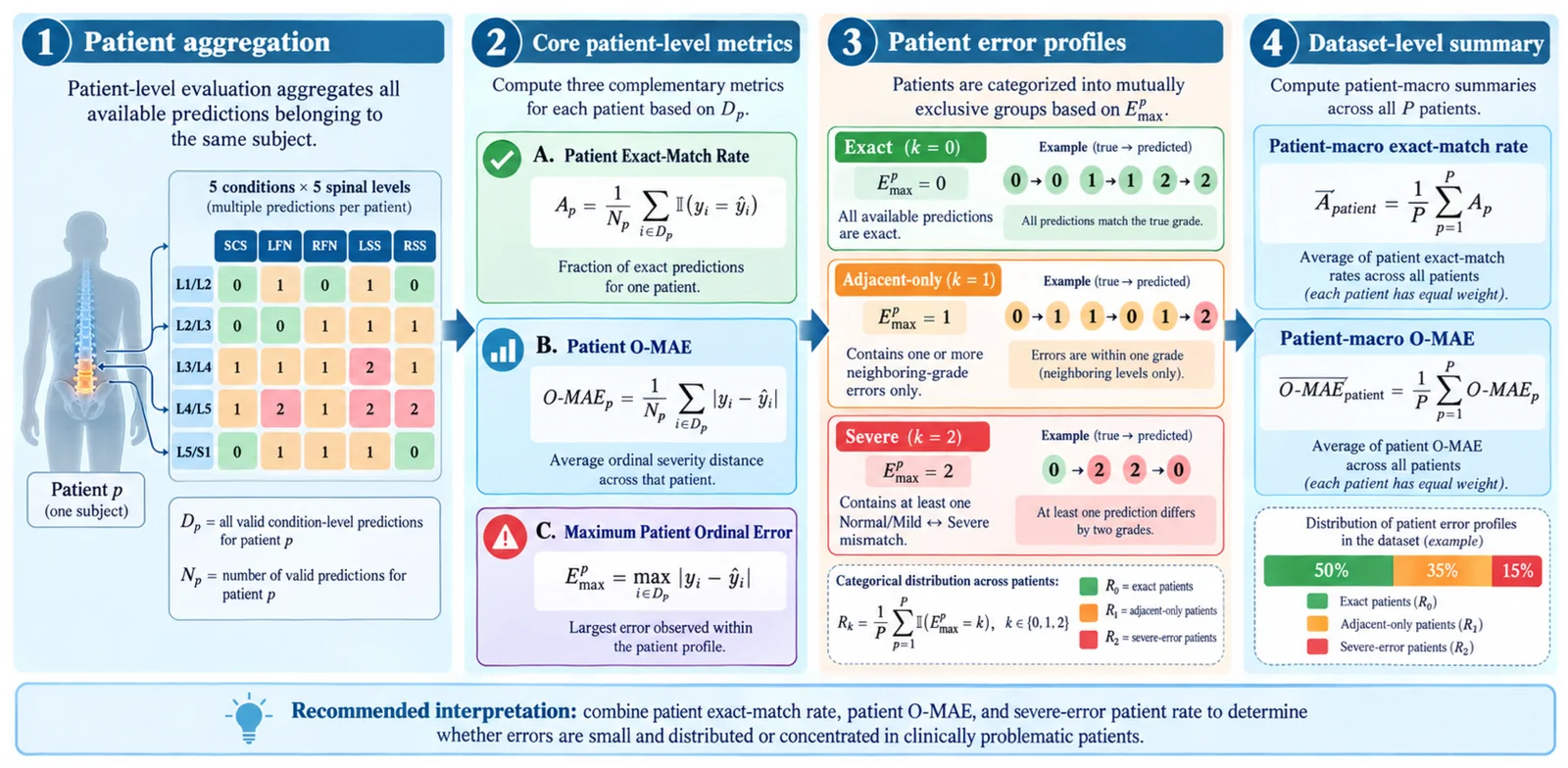
Overview of the patient-level evaluation dimension of the proposed Beyond-Accuracy framework. All valid condition–level predictions belonging to the same patient are aggregated into a structured patient profile. Patient-level Exact-Match Rate quantifies the proportion of exactly correct predictions, patient O-MAE measures the average ordinal severity displacement, and the Maximum Patient Ordinal Error identifies the largest disagreement within the patient assessment. Patients are subsequently categorized as *exact*, *adjacent-only*, or *severe-error* according to the maximum observed ordinal error. Dataset-level summaries are obtained by macro-averaging patient-level metrics, assigning equal weight to each patient.

Figure 8 summarizes the proposed patient-level evaluation strategy. The framework first groups all valid predictions belonging to the same patient, then derives complementary measures of exact agreement, average ordinal error, and maximum ordinal error. Finally, patients are partitioned into mutually exclusive error-profile categories according to the largest severity disagreement observed within their complete structured assessment.

For patient *p*, let

(26) $$D_{p}={\left\{(y_{p,j},{\hat{y}}_{p,j})\right\}}_{j=1}^{N_{p}},$$

denote the set of $N_{p}$ valid predictions available for that patient, where $y_{p,j}$ and ${\hat{y}}_{p,j}$ are the reference and predicted severity grades, respectively. The prediction index *j* may correspond to different condition–spinal-level combinations. Patients are evaluated using all available valid predictions rather than requiring an identical number of outputs for every subject.

**Patient-Level Exact-Match Rate.** The proportion of exactly correct severity predictions for patient *p* is defined as

(27) $$A_{p}=\frac{1}{N_{p}}\sum_{j=1}^{N_{p}}I\left(y_{p,j}={\hat{y}}_{p,j}\right),$$

where I(·) denotes the indicator function. A value of $A_{p}=1$ therefore indicates that every available prediction for the patient matches the reference grade, whereas smaller values indicate an increasing proportion of incorrect findings within the same examination.

The patient-macro exact-match rate is then computed as

(28) $${\bar{A}}_{\mathrm{patient}}=\frac{1}{P}\sum_{p=1}^{P}A_{p},$$

where *P* is the number of evaluated patients. Equal weighting of patients prevents subjects with a larger number of valid condition–level predictions from dominating the aggregate metric.

**Patient-Level Ordinal Error.** Exact agreement alone does not distinguish between adjacent- and maximum-distance severity errors. Therefore, the average ordinal displacement within each patient is quantified using patient-level ordinal mean absolute error,

(29) $${\text{O-MAE}}_{p}=\frac{1}{N_{p}}\sum_{j=1}^{N_{p}}\left|y_{p,j}-{\hat{y}}_{p,j}\right|.$$

Lower values indicate that the predicted severity profile remains closer to the reference assessment across the complete set of available findings. The patient-macro ordinal error is subsequently calculated as

(30) $${\bar{\text{O-MAE}}}_{\mathrm{patient}}=\frac{1}{P}\sum_{p=1}^{P}{\text{O-MAE}}_{p}.$$

**Maximum Patient Ordinal Error.** A mean patient-level error can still conceal an isolated but clinically important disagreement. To capture the largest severity deviation within each patient, the maximum ordinal error is defined as

(31) $$E_{p}^{\max}=\max_{j\in \{1,\ldots ,N_{p}\}}\left|y_{p,j}-{\hat{y}}_{p,j}\right|.$$

For the present three-class severity scale, $E_{p}^{\max}\in \left\{0,1,2\right\}$. A value of 0 indicates that all available predictions for the patient are exact. A value of 1 indicates that the patient contains at least one adjacent-grade error but no maximum-distance error. A value of 2 indicates the presence of at least one direct Normal/Mild–Severe disagreement.

Accordingly, the patient population is partitioned into three mutually exclusive error profiles:

(32) $$R_{k}=\frac{1}{P}\sum_{p=1}^{P}I\left(E_{p}^{\max}=k\right),\;k\in \left\{0,1,2\right\},$$

where $R_{0}$ represents patients for whom all available predictions are exact, $R_{1}$ represents patients affected only by adjacent-grade errors, and $R_{2}$ represents patients containing at least one severe two-grade error.

This patient-level perspective addresses a different question from the preceding evaluation dimensions. Global metrics summarize overall predictive performance, condition-specific analysis identifies pathology-dependent weaknesses, and spinal-level analysis localizes anatomical variation. The patient-level analysis instead determines whether those errors accumulate within individual examinations and whether a subset of patients is exposed to particularly severe prediction failures.

Two models with similar global ACC or Macro-F1 may therefore exhibit very different patient-level behavior. One model may distribute small adjacent errors across many patients, whereas another may produce fewer total errors but concentrate maximum-distance disagreements within a smaller patient subset. For this reason, patient exact-match rate, patient O-MAE, and the maximum-error distribution are interpreted jointly rather than in isolation.

Together, these measures provide the final layer of the proposed Beyond-Accuracy framework by characterizing not only whether individual predictions are correct, but also whether the complete structured assessment of each patient remains internally reliable and free from clinically consequential ordinal errors.

### 3.9. Code Availability and Reproducibility

The implementation of the LumbarDISC preprocessing, model-training, parallelization, and evaluation pipelines is publicly available in the project repository [25]. The repository includes the RSNA 2024 lumbar MRI processing workflow, condition-specific training code, neural architecture search utilities, repeated best-configuration retraining, and post hoc condition-, spinal-, and patient-level evaluation scripts.

## 4. Results

### 4.1. Exact Classification Performance

Before examining the ordinal structure of the prediction errors, the selected models were evaluated using the conventional exact classification metrics: Accuracy (ACC), Macro-Precision, Macro-Recall, and Macro-F1. These measures provide the standard categorical performance baseline and quantify exact severity-grade discrimination without considering the ordinal distance between incorrect predictions.

Table 1 summarizes the validation-set performance across all four architecture families, the two input representations, and the five degenerative conditions. Values are reported as mean ± standard deviation across five repeated runs. For each architecture, the final row represents the mean across SCS, LFN, RFN, LSS, and RSS.

Among the conventional 2D configurations, ConvNeXt achieved the strongest aggregate exact classification profile. Its mean ACC reached 0.8592, together with a Macro-Precision of 0.7385, Macro-Recall of 0.6726, and Macro-F1 of 0.6941. MaxViT achieved the second-highest mean ACC at 0.8446 and a Macro-F1 of 0.6625, while EfficientNet obtained a similar mean ACC of 0.8434 and a slightly higher Macro-F1 of 0.6691. The 2D ViT achieved a mean ACC of 0.8350 and Macro-F1 of 0.5932.

The condition-specific results further demonstrate that ACC alone does not fully describe balanced three-class discrimination. For example, the 2D ViT achieved an SCS ACC of 0.9200 with a Macro-F1 of 0.6968, whereas its LFN ACC remained relatively high at 0.7726 despite a much lower Macro-F1 of 0.2906. The corresponding Macro-Recall of 0.3333 indicates that the model was effectively unable to provide balanced sensitivity across the three severity classes for this condition.

Within the 2.5D-to-3D group, MaxViT clearly achieved the strongest conventional classification performance. Its mean ACC reached 0.8217, Macro-Precision 0.6581, Macro-Recall 0.5977, and Macro-F1 0.6156. This performance was substantially stronger than the corresponding ViT, ConvNeXt, and EfficientNet stacked-input configurations.

EfficientNet achieved a mean ACC of 0.7956 and Macro-F1 of 0.4004, while ViT reached 0.7819 and 0.3980, respectively. ConvNeXt showed the weakest balanced performance in the 2.5D-to-3D setting, with a mean ACC of 0.7702 and Macro-F1 of 0.2983. Its Macro-Recall values were close to 0.3333 for several conditions, suggesting that the predictions were dominated by a single severity class despite apparently moderate aggregate accuracy.

The architecture-dependent effect of incorporating cross-level anatomical context is therefore already visible under conventional classification metrics. MaxViT preserved the largest proportion of its 2D performance, with mean ACC decreasing from 0.8446 to 0.8217 and Macro-F1 decreasing from 0.6625 to 0.6156. In contrast, ConvNeXt exhibited a much larger deterioration, particularly in Macro-F1, which decreased from 0.6941 to 0.2983.

These results establish the conventional classification baseline for the subsequent Beyond-Accuracy analysis. Although ACC, Macro-Precision, Macro-Recall, and Macro-F1 describe exact and class-balanced classification performance, they do not distinguish between adjacent severity errors and maximum-distance Normal/Mild–Severe disagreements. The ordinal analysis therefore complements these metrics using OA, O-MAE, QWK, and SER.

### 4.2. Ordinal Severity Results

The conventional classification metrics presented previously quantify whether the predicted severity grade exactly matches the reference label, but they do not characterize the magnitude of incorrect predictions. This distinction is particularly important for the ordered three-class formulation adopted in this study, where the severity labels follow the progression Normal/Mild → Moderate → Severe. Consequently, the selected architectures were further evaluated using the ordinal severity metrics introduced in Section 3.8.2: Ordinal Accuracy (OA), Ordinal Mean Absolute Error (O-MAE), Quadratic Weighted Kappa (QWK), and Severe Ordinal Error Rate (SER).

In the implemented evaluation pipeline, OA represents the exact-match rate over the ordered severity labels and therefore provides the conventional correctness reference within the ordinal analysis. O-MAE complements OA by quantifying the average absolute distance between the predicted and reference severity grades. QWK evaluates agreement while assigning greater penalty to larger ordinal disagreements, whereas SER explicitly measures the frequency of maximum-distance Normal/Mild–Severe errors. A favorable ordinal profile is therefore characterized by higher OA and QWK together with lower O-MAE and SER.

Table 2 reports the validation-set ordinal severity performance across all four architecture families, both input representations, and the five degenerative conditions. Values are reported as mean ± standard deviation across five independent repeated runs. For each architecture, an additional mean row summarizes performance across SCS, LFN, RFN, LSS, and RSS.

Among the conventional 2D configurations, ConvNeXt achieved the strongest aggregate ordinal profile. Its mean OA reached 0.8592, while its mean O-MAE was limited to 0.1460. ConvNeXt additionally obtained the highest mean QWK of 0.7360 and the lowest mean SER of 0.0052. Thus, the same architecture that performed strongly under conventional classification metrics also produced the most favorable combination of exact severity agreement, small ordinal displacement, strong weighted agreement, and a very low frequency of maximum-distance severity errors.

MaxViT produced the second-strongest 2D profile for three of the four ordinal measures, achieving a mean OA of 0.8446, O-MAE of 0.1626, and SER of 0.0072. EfficientNet obtained a very similar mean OA of 0.8434 and O-MAE of 0.1647, while reaching a slightly higher QWK of 0.7182 compared with 0.7086 for MaxViT. The 2D ViT achieved a mean OA of 0.8350 and O-MAE of 0.1792, but its lower mean QWK of 0.5641 and higher SER of 0.0142 indicate greater variability in the ordinal structure of its errors.

The condition-specific results further illustrate why exact correctness alone does not fully characterize model behavior. For example, 2D ViT achieved an SCS OA of 0.9200 together with a low O-MAE of 0.0859, QWK of 0.7883, and SER of only 0.0059. In contrast, its LFN OA decreased to 0.7726, while O-MAE increased to 0.2678 and SER to 0.0405. The corresponding QWK was 0.0000, indicating that the apparent moderate exact-match performance was not accompanied by meaningful weighted ordinal agreement.

The 2.5D-to-3D results reveal a notably different architecture ranking. MaxViT achieved the strongest overall ordinal profile within this group, reaching a mean OA of 0.8217, O-MAE of 0.1939, QWK of 0.6201, and SER of 0.0156. This represents a substantially stronger ordinal result than the other 2.5D-to-3D configurations and indicates that MaxViT retained much more of its 2D ordinal behavior when stacked depth information was introduced.

EfficientNet ranked second in mean OA and O-MAE among the 2.5D-to-3D architectures, obtaining values of 0.7956 and 0.2437, respectively. Its mean QWK, however, decreased to 0.2196. ViT achieved a similar mean QWK of 0.2308 but lower OA and higher O-MAE, at 0.7819 and 0.2627, respectively. ConvNeXt exhibited the weakest ordinal behavior in the 2.5D-to-3D setting, with a mean OA of 0.7702, O-MAE of 0.2913, QWK of 0.0221, and SER of 0.0615.

The architecture-dependent effect of the representation is particularly clear when each 2D model is compared with its 2.5D-to-3D counterpart. MaxViT showed the smallest deterioration: mean OA decreased by only approximately 0.023, O-MAE increased by 0.031, QWK decreased by approximately 0.088, and SER increased by approximately 0.008. In comparison, the representation change produced substantially larger ordinal degradation for ViT, EfficientNet, and especially ConvNeXt. For ConvNeXt, mean QWK decreased from 0.7360 to 0.0221, while SER increased from 0.0052 to 0.0615.

These findings strengthen the central Beyond-Accuracy motivation of the study. OA indicates how frequently the model predicts the exact severity grade, but models with similar OA can exhibit markedly different O-MAE, QWK, and SER values. These complementary measures therefore distinguish models whose errors remain close to the reference severity from models that produce larger or more clinically consequential disagreements. The subsequent spinal-level and patient-level analyses further decompose these ordinal differences according to anatomical location and complete patient-level assessment.

#### 4.2.1. Ordinal Performance Trade-Off: QWK Versus O-MAE

**Figure 9:**
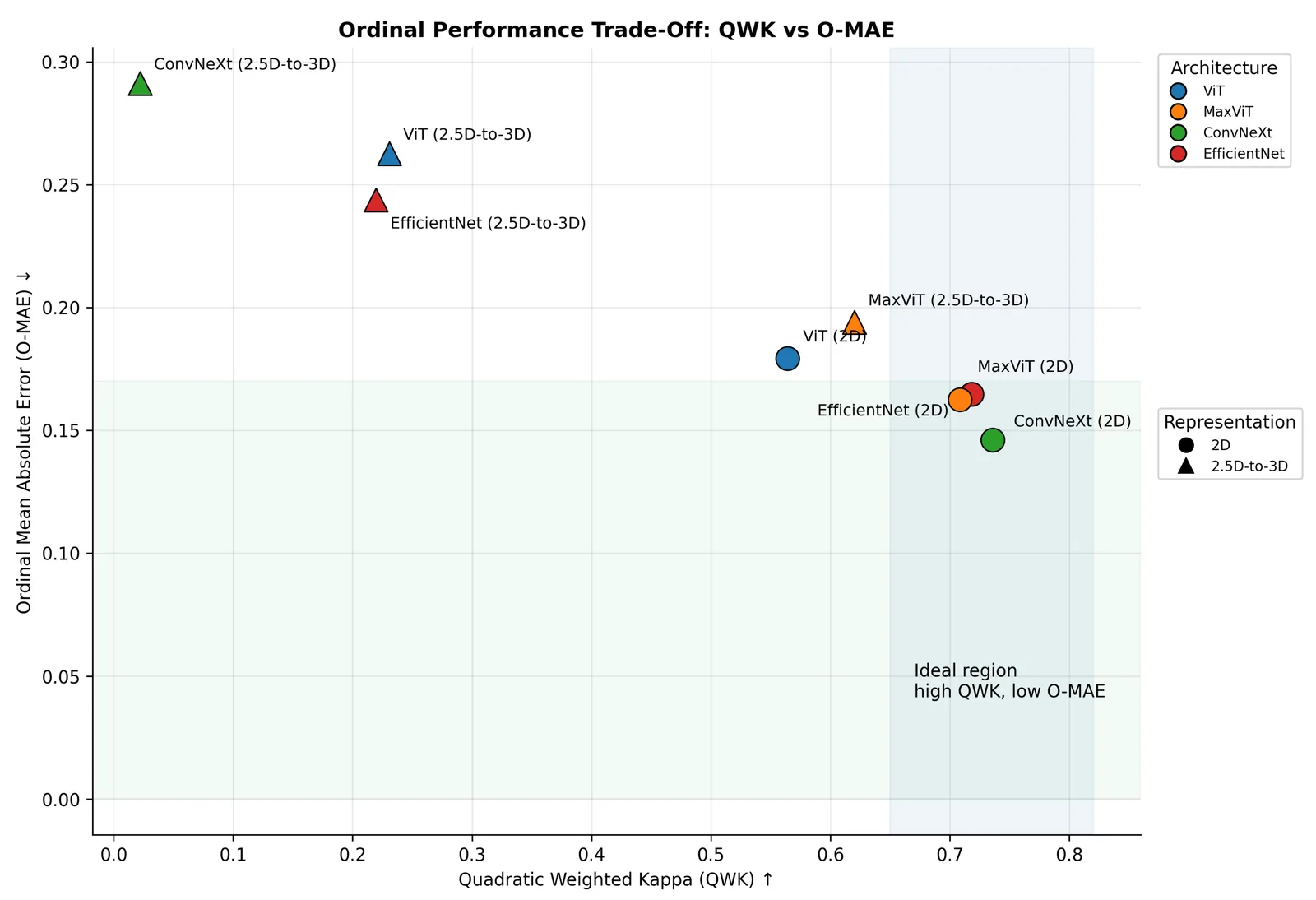
Validation-set ordinal performance trade-off between Quadratic Weighted Kappa (QWK) and Ordinal Mean Absolute Error (O-MAE). Each marker represents one architecture–representation configuration, averaged across the five lumbar degenerative conditions. Marker color identifies the architecture, while marker shape distinguishes 2D from 2.5D-to-3D models. Higher QWK (↑) and lower O-MAE (↓) indicate better ordinal performance. The light blue shaded region highlights higher QWK values, while the light green region highlights lower O-MAE values. Their overlap in the lower-right corner represents the preferred operating region. Shading is illustrative and does not indicate clinically validated performance thresholds.

To jointly assess ordinal agreement and the magnitude of severity misclassification, Figure 9 compares the validation-set Quadratic Weighted Kappa (QWK) and Ordinal Mean Absolute Error (O-MAE) for each architecture–representation configuration. Each point represents the mean performance across the five degenerative conditions (SCS, LFN, RFN, LSS, and RSS). Because higher QWK indicates stronger ordinal agreement and lower O-MAE indicates smaller average severity displacement, the most favorable configurations are located toward the lower-right region of the plot.

A clear separation is observed between the 2D and 2.5D-to-3D configurations. All four 2D models occupy the favorable high-QWK, low-O-MAE portion of the performance space. Among them, ConvNeXt achieved the strongest joint ordinal performance, with a mean QWK of 0.7360 and an O-MAE of 0.1460. EfficientNet and MaxViT followed closely, reaching QWK values of 0.7182 and 0.7086, together with O-MAE values of 0.1647 and 0.1626, respectively. The 2D ViT showed weaker ordinal agreement than the other 2D architectures, with QWK = 0.5641, although its O-MAE remained relatively low at 0.1792.

The 2.5D-to-3D models exhibited substantially greater dispersion. MaxViT was the strongest stacked-input configuration, reaching QWK =0.6201 and O-MAE =0.1939. Its position is considerably closer to the 2D cluster than the remaining 2.5D-to-3D models, indicating that MaxViT retained a larger fraction of its ordinal discrimination when cross-level anatomical context was introduced.

In contrast, the 2.5D-to-3D ViT and EfficientNet configurations occupied a less favorable region of the plot. ViT obtained QWK =0.2308 with O-MAE =0.2627, while EfficientNet reached QWK =0.2196 and O-MAE =0.2437. The largest degradation was observed for the 2.5D-to-3D ConvNeXt configuration, for which QWK decreased to 0.0221 and O-MAE increased to 0.2913. This combination indicates that, although the model may still produce a substantial number of exact predictions, the remaining predictions provide very limited ordinal agreement when considered across all conditions.

The trade-off plot also highlights why QWK and O-MAE provide complementary information. QWK measures agreement while accounting for the ordered nature of the severity categories, whereas O-MAE directly quantifies the average distance between predicted and reference grades. A configuration is therefore most desirable when it simultaneously achieves a high QWK and a low O-MAE, rather than optimizing either metric in isolation.

Overall, the visualization reinforces the broader findings of the ordinal analysis. The 2D ConvNeXt configuration provides the strongest aggregate ordinal behavior, whereas MaxViT is the most robust architecture within the 2.5D-to-3D setting. The separation between most 2D and cross-level 2.5D-to-3D models also suggests that incorporating anatomically ordered lumbar-level ROIs did not systematically improve ordinal severity assessment in the present pipeline. These differences should, however, be interpreted at the level of the complete architecture–representation configurations rather than as a causal effect of dimensionality alone, since the compared pipelines may also differ in pretraining, optimization behavior, and effective model capacity.

#### 4.2.2. Ordinal Metric Heatmaps

**Figure 10:**
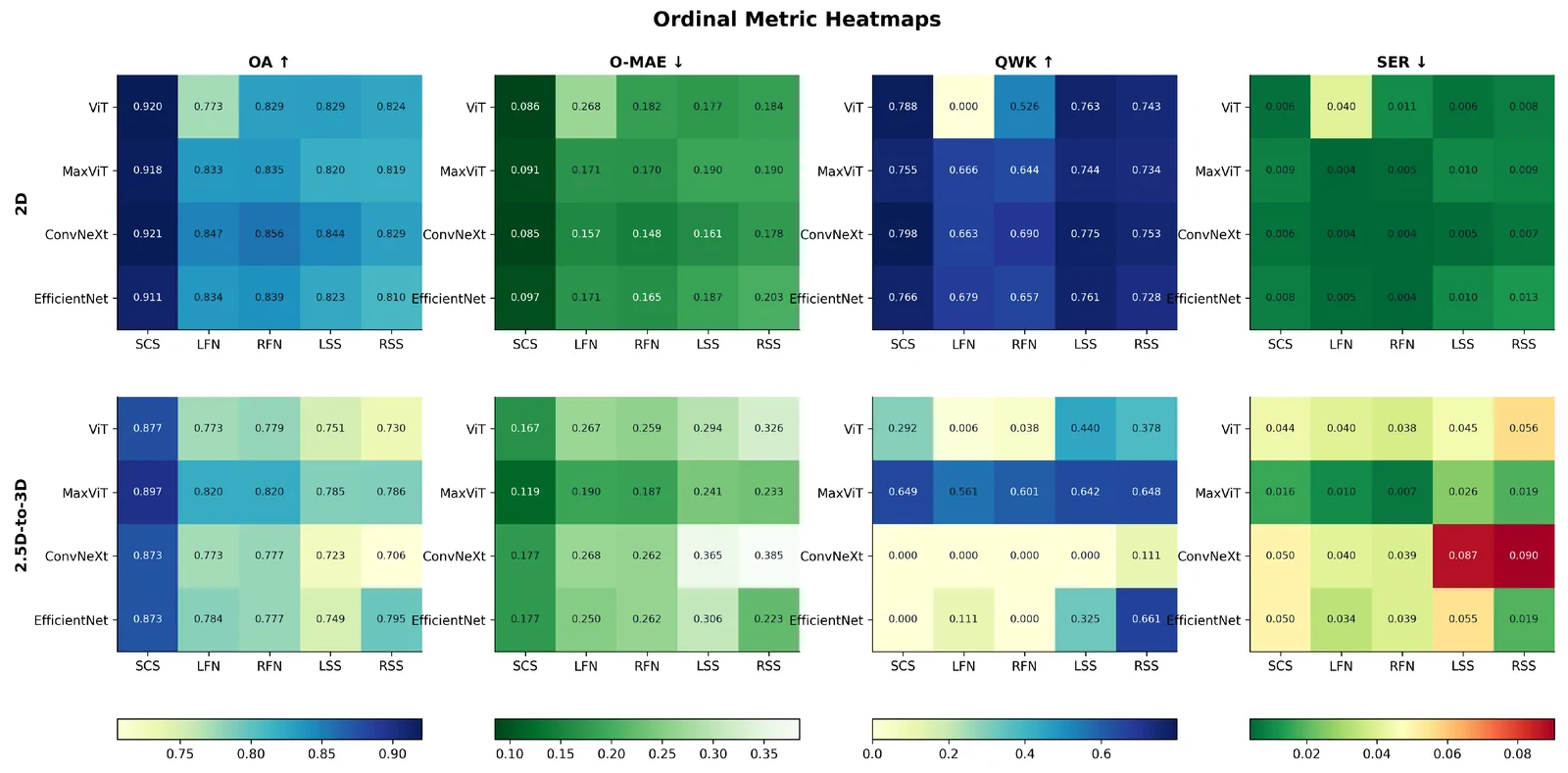
Heatmap-based summary of validation-set ordinal severity metrics across architectures and lumbar degenerative conditions. The upper row shows 2D models and the lower row shows 2.5D-to-3D models. From left to right, the panels report Ordinal Accuracy (OA), Ordinal Mean Absolute Error (O-MAE), Quadratic Weighted Kappa (QWK), and Severe Error Rate (SER). The upward arrow (↑) indicates that higher values are preferable (OA and QWK), whereas the downward arrow (↓) indicates that lower values are preferable (O-MAE and SER), reflecting smaller average severity displacement and fewer maximum-distance ordinal errors.

To provide a compact visual summary of the ordinal severity behavior across all architectures, conditions, and input representations, Figure 10 presents heatmaps for the four core ordinal metrics: Ordinal Accuracy (OA), Ordinal Mean Absolute Error (O-MAE), Quadratic Weighted Kappa (QWK), and Severe Error Rate (SER). The upper row corresponds to the 2D models, whereas the lower row summarizes the 2.5D-to-3D configurations. In each heatmap, rows denote architectures and columns denote lumbar degenerative conditions.

The heatmaps reveal several consistent patterns that are less immediately visible in the tabulated results alone. First, the 2D models are generally superior to their 2.5D-to-3D counterparts across all four ordinal metrics. This is evident from the darker high-value regions in the OA and QWK panels for the 2D models, together with the more favorable lower-value regions in the O-MAE and SER panels. In contrast, the 2.5D-to-3D configurations show more frequent degradation, especially in QWK and SER.

Second, among the 2D models, ConvNeXt provides the most consistently strong ordinal performance. It achieves high OA values across all five conditions, low O-MAE, strong QWK, and uniformly low SER. MaxViT and EfficientNet also perform well, although their performance is slightly less consistent across conditions. The 2D ViT model shows a more heterogeneous pattern, with strong performance for SCS, LSS, and RSS, but markedly weaker ordinal agreement for LFN, where QWK drops to 0.000 despite a nontrivial OA. This combination highlights one of the central motivations of the Beyond-Accuracy framework: exact or near-exact correctness rates alone can conceal poor ordinal structure.

Third, the lower heatmaps show that MaxViT is the strongest architecture in the 2.5D-to-3D setting. It retains relatively competitive OA, the lowest O-MAE values among the stacked-input models, and substantially better QWK than the other 2.5D-to-3D architectures. By contrast, the 2.5D-to-3D ConvNeXt configuration is the weakest overall, exhibiting near-zero QWK for most conditions and the highest SER values, especially for LSS and RSS. EfficientNet 2.5D-to-3D also shows unstable ordinal behavior, with acceptable performance for RSS but weak QWK for SCS and RFN.

The condition-wise structure is also informative. SCS emerges as the most favorable condition for ordinal severity assessment across most architectures, typically showing the highest OA and QWK together with the lowest O-MAE and SER. In contrast, LFN remains challenging for several models, particularly ViT and ConvNeXt in the 2.5D-to-3D setting. Lower lumbar conditions such as LSS and RSS also appear more difficult in the stacked-input configurations, especially when considering QWK and SER.

Overall, Figure 10 provides a dense but highly informative summary of the ordinal evaluation landscape. It confirms that the best-performing models are not only those with high overall correctness, but those that maintain strong ordinal agreement, small severity displacement, and low severe-error burden across all five degenerative conditions. In this respect, the heatmaps reinforce the conclusion that 2D ConvNeXt and 2D MaxViT offer the most favorable ordinal profiles, whereas among the 2.5D-to-3D variants, MaxViT remains the most competitive configuration.

#### 4.2.3. 2D Versus 2.5D-to-3D Ordinal Degradation/Gain

**Figure 11:**
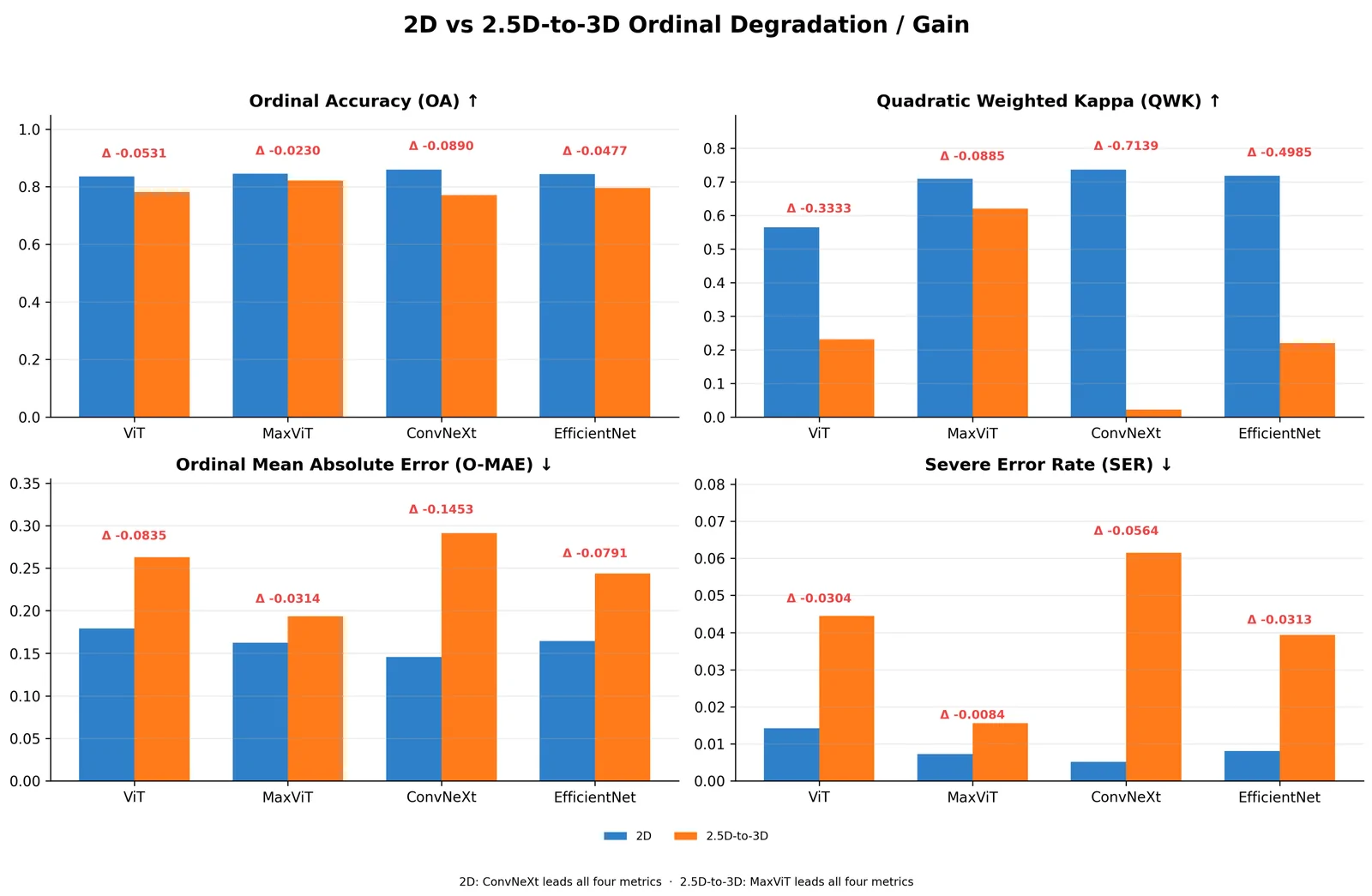
Comparison of mean validation-set ordinal performance between 2D and 2.5D-to-3D configurations across the four evaluated architectures. The upward arrows (↑) indicate higher-is-better metrics (OA and QWK), whereas the downward arrows (↓) indicate lower-is-better metrics (O-MAE and SER). Red annotations indicate the performance change from 2D to 2.5D-to-3D for each architecture. Negative differences in OA and QWK indicate reduced ordinal agreement, whereas positive differences in O-MAE and SER indicate increased severity displacement and a higher frequency of severe ordinal errors.

To summarize the effect of changing the input representation from 2D crops to 2.5D-to-3D stacked inputs, Figure 11 compares the mean validation ordinal metrics across the four model architectures. The four panels report Ordinal Accuracy (OA), Quadratic Weighted Kappa (QWK), Ordinal Mean Absolute Error (O-MAE), and Severe Error Rate (SER), thereby providing a compact Beyond-Accuracy view of the representation shift.

Figure 11 shows that the transition from 2D to 2.5D-to-3D does not improve ordinal performance for any of the four architectures. Instead, all models exhibit degradation in the agreement-based metrics and simultaneous worsening in the error-based metrics. This pattern is consistent across OA, QWK, O-MAE, and SER, indicating that the decline is not limited to strict correctness alone, but extends to the ordinal structure of the predictions.

For OA, the degradation is relatively modest in absolute terms, ranging from Δ=−0.0230 for MaxViT to Δ=−0.0890 for ConvNeXt. However, the QWK panel reveals a much stronger separation between the two representations. The most pronounced drop is observed for ConvNeXt, where QWK decreases by −0.7139, followed by EfficientNet (−0.4985) and ViT (−0.3333). MaxViT remains the most stable architecture under the representation change, with a smaller QWK reduction of −0.0885. This is an important result because QWK more directly reflects the quality of ordinal agreement, not just whether predictions remain close in a broad average sense.

The error-oriented panels reinforce the same conclusion. O-MAE increases for all architectures, meaning that predictions move farther from the correct severity grade on average when the 2.5D-to-3D formulation is used. Again, ConvNeXt shows the largest deterioration, with an increase of +0.1453, whereas MaxViT shows the smallest increase of +0.0314. Likewise, SER increases for every architecture, showing that 2.5D-to-3D models are more likely to produce clinically undesirable far-miss errors. The largest SER increase is again observed for ConvNeXt (+0.0564), while MaxViT remains the most resilient (+0.0084).

Two high-level conclusions emerge from this figure. First, the best ordinal performance in the current study is achieved by the 2D models, with ConvNeXt leading the 2D group across all four mean ordinal metrics. Second, among the 2.5D-to-3D models, MaxViT is the strongest configuration and the one that retains the largest fraction of the 2D ordinal behavior. Therefore, the present results do not support an ordinal advantage for the current 2.5D-to-3D setting; instead, they indicate that the stacked-input formulation, as implemented here, introduces a substantial degradation in severity-aware prediction quality.

From a Beyond-Accuracy perspective, this figure is particularly useful because it demonstrates that representation changes should not be evaluated only by overall exact classification metrics. Even when exact accuracy drops only moderately, the ordinal degradation can be much more severe, especially in QWK and SER. The plot therefore strengthens the central argument of the paper: a meaningful comparison between lumbar MRI classification pipelines requires not only exact classification performance, but also explicit evaluation of how well each model preserves the ordered structure of the severity labels.

#### 4.2.4. Severe Ordinal Error Burden

**Figure 12:**
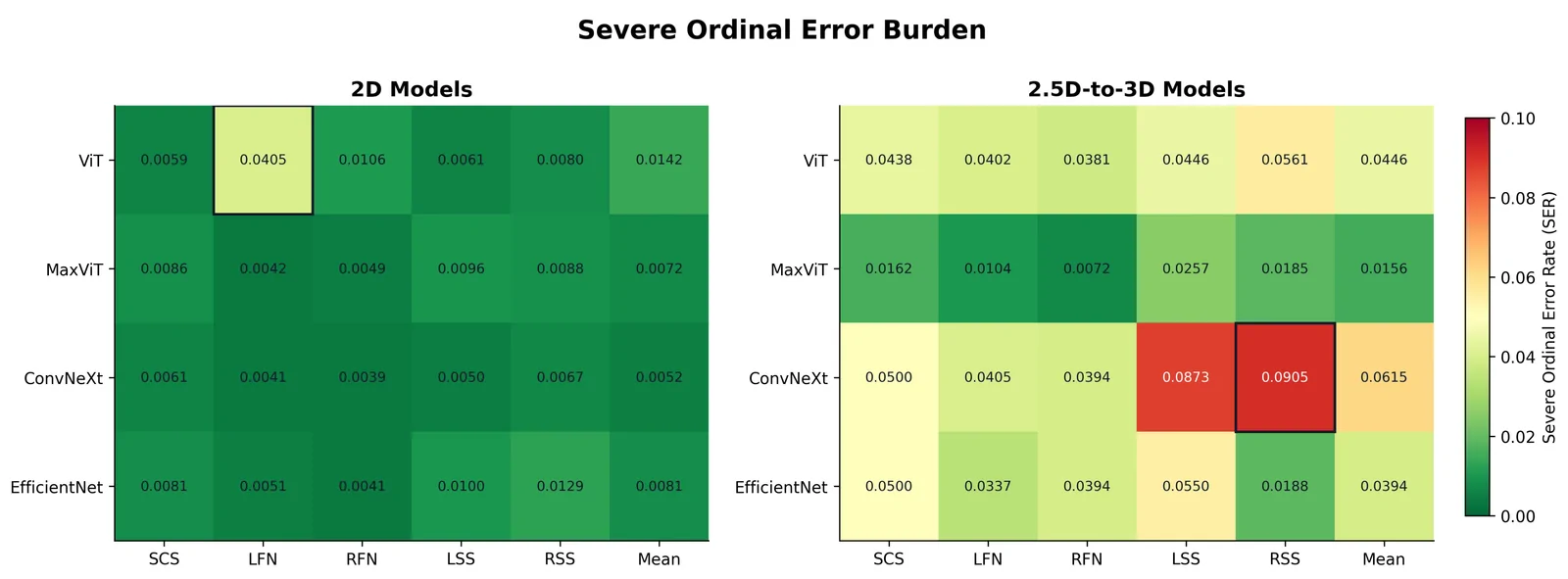
Validation-set Severe Error Rate (SER) across model architectures and lumbar degenerative conditions for 2D and 2.5D-to-3D configurations. Lower SER is preferred. Each cell reports the fraction of direct Normal/Mild ↔ Severe misclassifications. The final column summarizes the mean SER across the five conditions for each model. Black outlines highlight particularly important condition-specific burden areas.

While OA, O-MAE, and QWK summarize overall ordinal behavior, they do not directly isolate the most clinically undesirable failure mode, namely maximum-distance misclassification between the extreme severity categories. For this reason, Figure 12 visualizes the Severe Error Rate (SER) across all lumbar degenerative conditions and model configurations. In the present three-class setting, SER measures the fraction of predictions for which the ordinal error magnitude is at least two grades, that is, direct *Normal/Mild* ↔ *Severe* confusions.

Several important trends emerge from Figure 12. First, the 2D models exhibit uniformly low severe error rates. Their mean SER values range from 0.0052 for ConvNeXt to 0.0142 for ViT, with all condition-specific values remaining close to zero. This indicates that, even when the 2D models are incorrect, they only rarely produce maximum-distance ordinal mistakes. Among the 2D configurations, ConvNeXt achieves the most favorable severe-error profile overall, followed by MaxViT and EfficientNet, whereas ViT shows the highest mean SER mainly due to the LFN condition.

Second, the 2.5D-to-3D models show a markedly larger severe-error burden. Their mean SER values increase to 0.0446 for ViT, 0.0156 for MaxViT, 0.0615 for ConvNeXt, and 0.0394 for EfficientNet. This confirms that the degradation observed in the broader ordinal metrics is not limited to modest adjacent errors, but also includes a substantially larger number of direct extreme misclassifications. In this representation setting, MaxViT remains the strongest model, preserving relatively low SER across most conditions, whereas ConvNeXt becomes the weakest configuration by a considerable margin.

Condition-specific analysis further reveals where the severe-error burden is concentrated. For the 2D models, the main bottleneck appears in LFN for ViT (0.0405), whereas the other architectures keep LFN much better controlled. For the 2.5D-to-3D models, the burden becomes more diffuse and substantially larger. The most pronounced failure pattern is observed for ConvNeXt in LSS (0.0873) and RSS (0.0905), which are the highest SER values in the entire figure. ViT also shows elevated severe error rates across all five conditions, especially for RSS (0.0561). EfficientNet remains intermediate, with particularly large burden in SCS (0.0500) and LSS (0.0550).

From a Beyond-Accuracy perspective, this figure is particularly informative because it isolates the clinically most serious ordinal failure mode. A model may still retain moderately acceptable OA or O-MAE while producing an undesirably high fraction of direct Normal/Mild ↔ Severe errors. Figure 12 therefore complements the previous ordinal analyses by showing not only whether predictions are close on average, but also whether the model avoids the most consequential severity confusions. Overall, the results confirm that the current 2D configurations are substantially safer from an ordinal-error standpoint, whereas the 2.5D-to-3D formulations, especially ConvNeXt and ViT, carry a much larger severe ordinal error burden.

### 4.3. Condition-Specific Evaluation Results

The preceding exact-classification and ordinal analyses established the overall predictive behavior of the evaluated architectures. However, these aggregate results do not indicate whether performance is distributed uniformly across the five lumbar degenerative conditions or is driven predominantly by a subset of easier tasks. The condition-specific analysis therefore evaluates SCS, LFN, RFN, LSS, and RSS independently and subsequently summarizes their cross-condition behavior using the condition-macro mean, best-performing condition, worst-performing condition, condition range, and standard deviation across conditions.

To avoid duplicating the complete metric sets already reported in the exact classification and ordinal severity subsections, the present analysis focuses on four complementary measures: Macro-F1 as the principal class-balanced classification metric, QWK as the weighted ordinal-agreement metric, O-MAE as the average severity-distance measure, and SER as the rate of maximum-distance severity errors. For Macro-F1 and QWK, higher values indicate better performance, whereas lower O-MAE and SER values are preferred.

Table 3 summarizes the validation-set condition-specific results. The condition-macro mean assigns equal weight to SCS, LFN, RFN, LSS, and RSS. The best- and worst-condition columns identify the strongest and weakest degenerative findings for each metric, while the condition range and standard deviation quantify the degree of performance variation across the five conditions.

The 2D architectures exhibited substantially more uniform condition-specific behavior than several of their 2.5D-to-3D counterparts. Among the 2D models, EfficientNet achieved the highest condition-macro Macro-F1 at 0.6812 and, more importantly, the smallest Macro-F1 condition range ($\Delta_{\mathrm{cond}}=0.0340$). MaxViT was similarly stable, reaching a condition-macro Macro-F1 of 0.6797 with a range of only 0.0509. These results indicate that both architectures maintained relatively consistent balanced classification performance across SCS, LFN, RFN, LSS, and RSS.

The 2D MaxViT and EfficientNet models also exhibited comparatively uniform ordinal agreement. EfficientNet achieved a condition-macro QWK of 0.7192 with a condition range of 0.0848, whereas MaxViT reached a slightly higher mean QWK of 0.7242 with a range of 0.1238. In contrast, the 2D ViT showed marked condition dependence: its QWK ranged from 0.7891 for SCS to 0.0000 for LFN, producing a condition range of 0.7891. Its Macro-F1 exhibited a similarly large range of 0.4298, identifying LFN as the principal condition-specific bottleneck.

ConvNeXt achieved strong average 2D performance but showed greater condition–level variability than MaxViT and EfficientNet. Its Macro-F1 ranged from 0.7293 for LSS to 0.5286 for LFN, while QWK ranged from 0.7948 for SCS to 0.4179 for LFN. Thus, although its overall exact and ordinal results were competitive, its performance was less uniform across pathologies than the more stable MaxViT and EfficientNet profiles.

The lower-is-better severity measures reinforce these findings. For the 2D models, MaxViT produced the lowest condition-macro SER at 0.0064 and the smallest SER range of 0.0062, indicating both a low overall frequency of maximum-distance errors and limited variation across conditions. ConvNeXt achieved the lowest condition-macro O-MAE at 0.1503, whereas MaxViT and EfficientNet followed closely at 0.1577 and 0.1610, respectively. The 2D ViT again showed the greatest condition dependence, largely due to its weaker LFN performance.

Among the 2.5D-to-3D configurations, MaxViT displayed the strongest and most consistent condition-specific profile. It achieved a condition-macro Macro-F1 of 0.5956 with a range of 0.0800 and a QWK of 0.6021 with a range of 0.0979. Its SER remained low at 0.0178 and varied by only 0.0165 across conditions. These results indicate that MaxViT preserved relatively balanced behavior across the five degenerative findings despite the transition to the stacked-input representation.

The remaining 2.5D-to-3D models exhibited substantially stronger condition dependence. ViT showed moderate variability, with Macro-F1 ranging from 0.3201 for LFN to 0.4793 for RSS. EfficientNet exhibited the largest Macro-F1 condition range among the evaluated configurations ($\Delta_{\mathrm{cond}}=0.3453$), primarily because RSS remained relatively strong while RFN deteriorated markedly. Its QWK showed an even wider range, from 0.0000 for RFN to 0.6658 for RSS.

The 2.5D-to-3D ConvNeXt configuration showed a different failure pattern. Its Macro-F1 values were uniformly low across conditions, resulting in a small range of 0.0315; however, this apparent uniformity should not be interpreted as robust performance. QWK was effectively zero across the five conditions, while mean O-MAE and SER reached 0.2867 and 0.0627, respectively. Thus, a small condition range alone is insufficient to identify a strong model: uniformity must be interpreted jointly with the absolute performance level.

Taken together, the condition-specific analysis demonstrates that two pipelines with similar aggregate performance can differ substantially in their pathology-dependent behavior. The condition-macro mean describes the overall level of performance, whereas the worst-condition score, $\Delta_{\mathrm{cond}}$, and $\sigma_{\mathrm{cond}}$ reveal whether that performance is distributed consistently across SCS, LFN, RFN, LSS, and RSS. The results particularly identify 2D MaxViT and EfficientNet as the most uniform conventional configurations, while 2.5D-to-3D MaxViT provides the strongest condition-specific stability among the stacked-input models.

These numerical summaries will subsequently be complemented by graphical condition profiles, including radar representations of SCS, LFN, RFN, LSS, and RSS, to visualize both absolute performance and cross-condition uniformity.

#### 4.3.1. Condition-Specific Reliability Profiles

**Figure 13:**
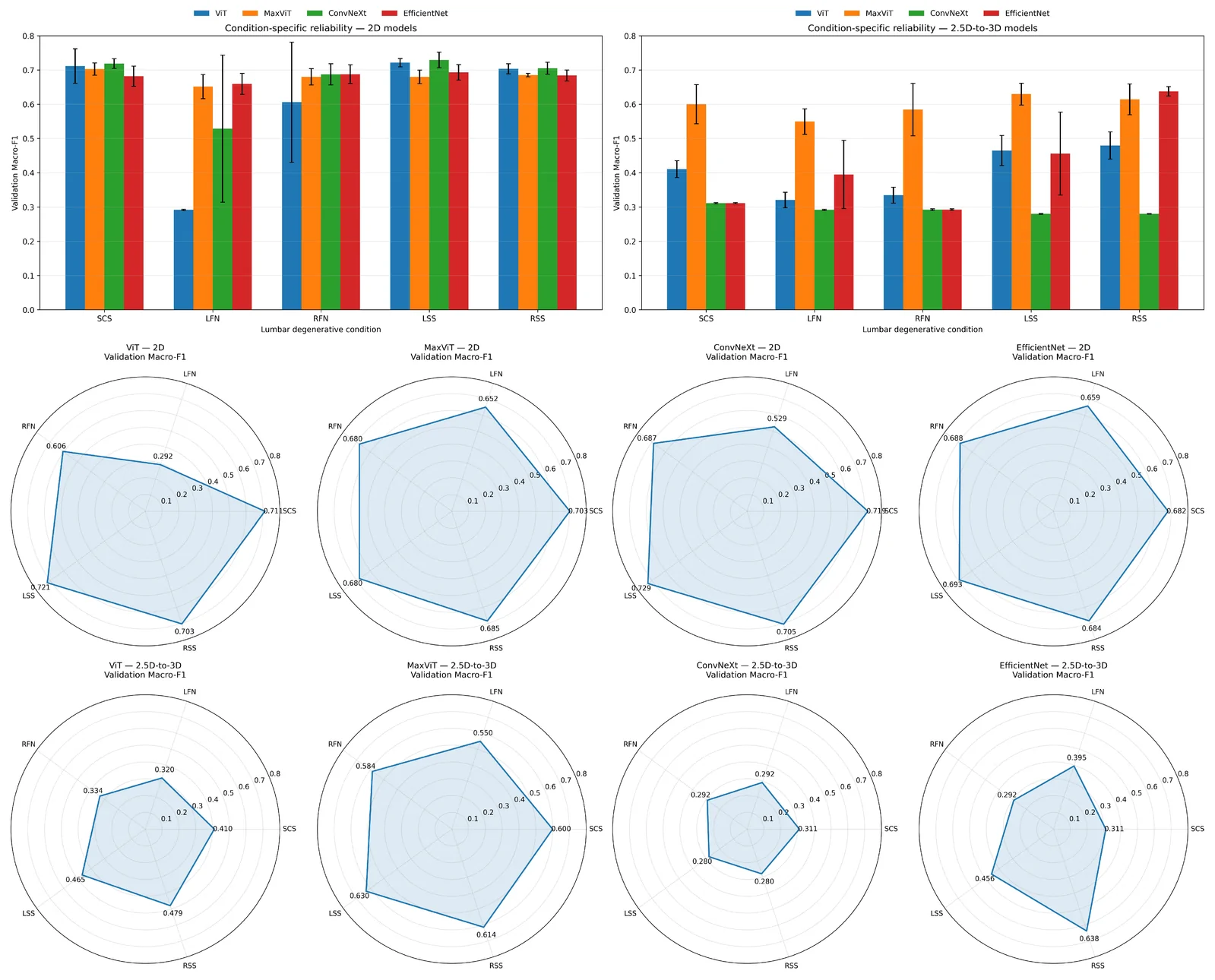
Condition-specific validation Macro-F1 profiles across the five lumbar degenerative conditions. The upper panels compare ViT, MaxViT, ConvNeXt, and EfficientNet using grouped bars for the 2D and 2.5D-to-3D representations, with error bars indicating variability across repeated runs. The lower radar plots show the corresponding architecture-specific condition profiles over SCS, LFN, RFN, LSS, and RSS. Larger and more symmetric radar profiles indicate stronger and more uniform condition-specific balanced classification performance.

To further characterize which degenerative conditions are modeled most reliably, Figure 13 visualizes the condition-specific validation Macro-F1 values across the four evaluated architecture families. The upper panels provide grouped bar-chart comparisons for the conventional 2D and 2.5D-to-3D configurations, with error bars representing variability across repeated runs. The lower panels complement these comparisons with radar profiles for each individual architecture, allowing both absolute performance and cross-condition uniformity to be inspected simultaneously.

For the 2D models, the condition profiles are generally broader and more uniform than those of the corresponding 2.5D-to-3D configurations. MaxViT and EfficientNet exhibit particularly balanced 2D profiles, with relatively small differences across the five conditions. ConvNeXt also performs strongly for most conditions, although its weaker LFN result produces a visibly less uniform profile. The 2D ViT shows the strongest condition dependence, with a pronounced reduction for LFN compared with SCS, RFN, LSS, and RSS.

The 2.5D-to-3D profiles reveal substantially greater architecture-dependent variation. MaxViT retains the strongest and most uniform Macro-F1 profile among the stacked-input models, maintaining comparatively high values across all five conditions. In contrast, the 2.5D-to-3D ViT exhibits substantially lower balanced classification performance, particularly for LFN and RFN. ConvNeXt shows consistently low Macro-F1 across all conditions, producing a compact radar profile that reflects uniformly weak rather than uniformly strong performance. EfficientNet exhibits a strongly asymmetric profile, with RSS remaining comparatively strong while SCS and RFN are substantially weaker.

The combined bar and radar representations therefore provide complementary information. The bar charts facilitate direct quantitative comparison between architectures for each degenerative condition, whereas the radar plots expose the shape and uniformity of each architecture’s condition-specific behavior. Importantly, profile symmetry alone should not be interpreted as model reliability: a compact but symmetric profile may indicate consistently poor performance. Condition uniformity must therefore be interpreted jointly with the absolute metric values, consistent with the condition-macro mean, worst-condition, and condition-range analyses reported above.

#### 4.3.2. Condition-Specific Bottleneck and Uniformity Analysis

**Figure 14:**
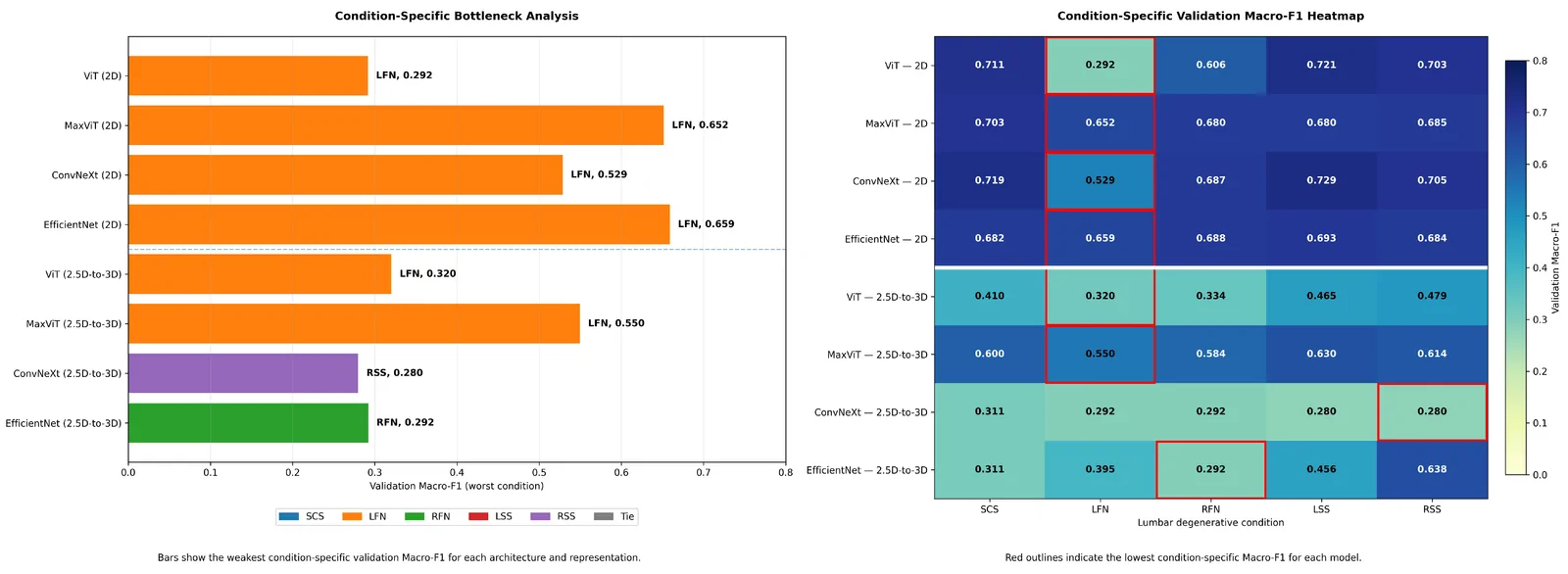
Condition-specific bottleneck and uniformity analysis based on validation Macro-F1. (**Left**): Bottleneck analysis showing, for each architecture and representation, the weakest condition-specific validation Macro-F1 together with the associated lumbar degenerative condition. (**Right**): Full heatmap of condition-specific validation Macro-F1 values across the five conditions (SCS, LFN, RFN, LSS, RSS). Red outlines denote the lowest condition-specific Macro-F1 within each model row. The dashed horizontal separator distinguishes 2D from 2.5D-to-3D models.

To further interpret the condition-specific results, Figure 14 summarizes two complementary views of model behavior across the five lumbar degenerative conditions. The left panel presents a bottleneck analysis, showing for each architecture and representation the weakest condition-specific validation Macro-F1. The right panel presents the full condition-by-model heatmap, where red outlines highlight the lowest validation Macro-F1 within each row. Together, these two visualizations address two practical questions of the Beyond-Accuracy framework: *which condition is the main performance bottleneck?* and *how uniform is model behavior across conditions?*

Several observations can be drawn from Figure 14. First, among the 2D models, *Left Neural Foraminal Narrowing* (LFN) is consistently the main bottleneck. It is the weakest condition for all four 2D architectures, with validation Macro-F1 values of 0.292 for ViT, 0.652 for MaxViT, 0.529 for ConvNeXt, and 0.659 for EfficientNet. This repeated pattern indicates that LFN is the most challenging condition in the 2D setting, particularly for the baseline ViT model, whose performance drops substantially on this task compared with the other four conditions.

Second, the 2D models are generally more uniform and more reliable than their 2.5D-to-3D counterparts. In the heatmap, the 2D rows remain concentrated in a relatively high range, while the 2.5D-to-3D rows show a wider spread and more pronounced weak spots. MaxViT is again the most stable architecture overall, with both its 2D and 2.5D-to-3D versions maintaining comparatively balanced performance across the five conditions. In contrast, the 2.5D-to-3D ViT, ConvNeXt, and EfficientNet models exhibit larger drops for specific conditions.

Third, the dominant bottleneck is less consistent in the 2.5D-to-3D setting. LFN remains the weakest condition for ViT and MaxViT, with validation Macro-F1 values of 0.320 and 0.550, respectively. However, ConvNeXt reaches its lowest value at RSS (tied with LSS at 0.280, with RSS highlighted in the bottleneck chart), while EfficientNet reaches its minimum at RFN (0.292). This shift suggests that the move from 2D to 2.5D-to-3D does not simply reduce performance uniformly, but changes the difficulty profile across conditions.

Finally, the combined figure shows why a purely aggregate comparison is insufficient. Two models may have similar average validation performance while behaving differently across the five conditions. The bottleneck plot provides a concise summary of the weakest point of each model, whereas the heatmap shows whether that bottleneck is isolated or part of a generally uneven condition profile. Together, these views complement the condition-specific tables and support the broader Beyond-Accuracy interpretation that model quality should be assessed not only by overall score but also by the distribution of performance across clinically distinct lumbar degenerative findings.

### 4.4. Spinal-Level Evaluation Results

The condition-specific analysis demonstrated that model performance varies across the five degenerative findings. The spinal-level evaluation further examines whether predictive behavior is also dependent on anatomical location along the lumbar spine. Predictions were therefore decomposed across the five intervertebral levels L1/L2, L2/L3, L3/L4, L4/L5, and L5/S1.

To maintain a compact presentation, the spinal-level results are reported separately for the conventional 2D models and the corresponding 2.5D-to-3D configurations. For each spinal level, results are macro-averaged across SCS, LFN, RFN, LSS, and RSS. Four complementary metrics are retained: Macro-F1 for balanced exact classification, QWK for ordinal agreement, O-MAE for average severity displacement, and SER for maximum-distance Normal/Mild–Severe errors. The final two columns summarize the equal-weight mean across the five spinal levels and the corresponding anatomical range $\Delta_{\mathrm{level}}$.

#### 4.4.1. Spinal-Level Performance of 2D Models

Table 4 summarizes the validation-set spinal-level performance of the four conventional 2D architecture families.

A consistent anatomical pattern is visible among the 2D architectures. Macro-F1 generally increases from the upper lumbar spine toward the middle and lower lumbar levels, with all four architectures reaching their highest balanced classification performance at L4/L5. The corresponding L4/L5 Macro-F1 values are 0.608 for ViT, 0.696 for MaxViT, 0.682 for ConvNeXt, and 0.676 for EfficientNet. Conversely, L1/L2 produces the lowest Macro-F1 for each architecture.

QWK exhibits a similar pattern. Weighted ordinal agreement peaks at L4/L5 for all four models, reaching 0.586, 0.742, 0.693, and 0.721 for ViT, MaxViT, ConvNeXt, and EfficientNet, respectively. Among the 2D pipelines, ConvNeXt provides the highest mean spinal-level Macro-F1 (0.593) and QWK (0.595), while also achieving the lowest mean O-MAE (0.152). MaxViT, however, produces the lowest mean SER (0.007).

The error-distance metrics reveal an important complementary trend. O-MAE is lowest at L1/L2 for all four architectures and increases toward the lower lumbar spine. Thus, despite stronger Macro-F1 and QWK at L4/L5, incorrect predictions at this level generally exhibit larger severity displacement. SER follows a similar cranio-caudal tendency and typically reaches its highest values at L5/S1, indicating a greater frequency of direct Normal/Mild–Severe disagreements in the lower lumbar region.

#### 4.4.2. Spinal-Level Performance of 2.5D-to-3D Models

Table 5 presents the corresponding results for the 2.5D-to-3D configurations.

The 2.5D-to-3D results demonstrate considerably stronger architecture dependence. MaxViT clearly provides the strongest stacked-input spinal-level profile, achieving the highest mean Macro-F1 (0.501) and QWK (0.432), as well as the lowest mean O-MAE (0.192) and SER (0.017). Its strongest balanced and ordinal performance again occurs at L4/L5, where Macro-F1 and QWK reach 0.608 and 0.603, respectively.

ViT and EfficientNet exhibit a broadly similar anatomical pattern, with Macro-F1 and QWK increasing toward L4/L5 but accompanied by progressively larger severity-distance errors. For ViT, O-MAE rises from 0.063 at L1/L2 to 0.462 at L4/L5, while SER increases from 0.011 to 0.084. EfficientNet shows a similar increase from 0.060 to 0.433 in O-MAE and from 0.010 to 0.083 in SER.

The 2.5D-to-3D ConvNeXt configuration exhibits the clearest spinal-level failure pattern. Its Macro-F1 decreases from 0.325 at L1/L2 to 0.242 at L4/L5, while QWK remains zero across all five levels. Simultaneously, O-MAE increases from 0.062 to 0.569 and SER from 0.011 to 0.148. Consequently, L4/L5 represents a particularly pronounced anatomical bottleneck for this configuration.

#### 4.4.3. Cross-Representation Spinal-Level Interpretation

Taken together, the two tables reveal that the notion of a “best” or “worst” spinal level depends strongly on the metric under consideration. For most architectures, L4/L5 provides the strongest Macro-F1 and QWK, indicating improved balanced classification and ordinal agreement at this level. However, L4/L5 and L5/S1 simultaneously exhibit larger O-MAE and SER values, showing that incorrect predictions in the lower lumbar region can carry greater ordinal severity.

Conversely, L1/L2 generally exhibits the smallest O-MAE and SER values but weaker Macro-F1 and, for several architectures, lower QWK. This apparent contrast likely reflects the interaction between anatomical level and severity-class distribution: an anatomically easier level may yield small average error distances while still providing limited discrimination among minority severity classes.

The spinal-level analysis therefore adds information that cannot be recovered from a single global metric. It demonstrates both *where* balanced severity classification is strongest and *where* the consequences of misclassification are greatest. These anatomically localized findings provide the final intermediate layer between the condition-specific analysis and the subsequent patient-level evaluation.

#### 4.4.4. Spinal-Level Macro-F1 Profiles

**Figure 15:**
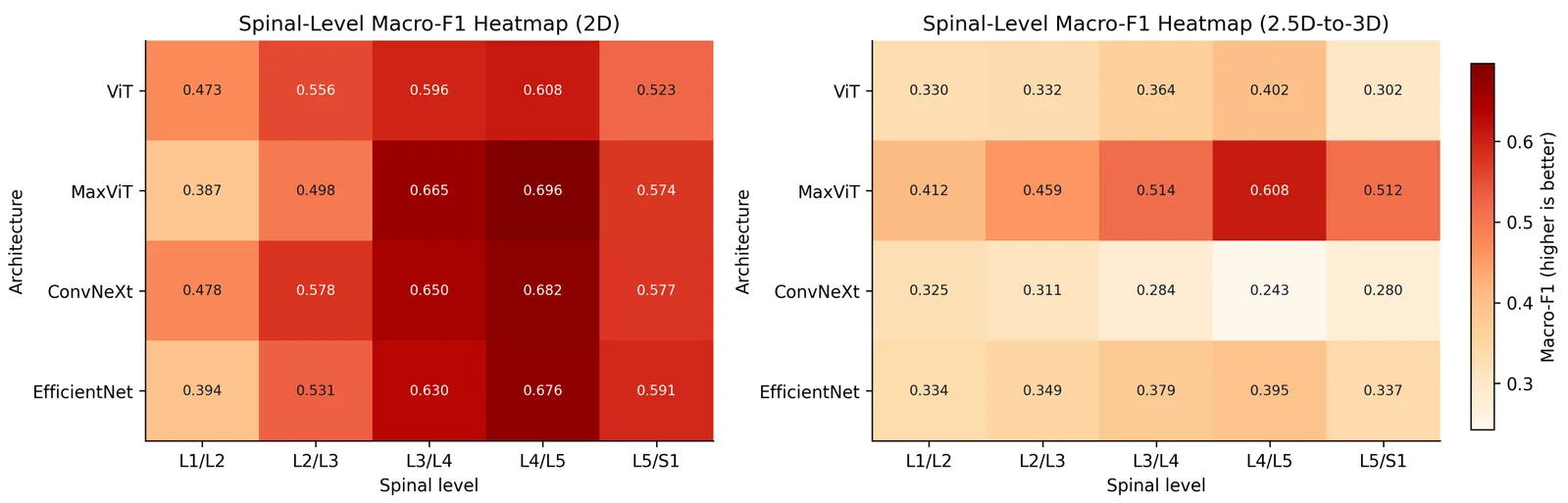
Spinal-level validation Macro-F1 across the five lumbar intervertebral levels for the 2D and 2.5D-to-3D model configurations. Rows correspond to the evaluated architecture families, while columns represent L1/L2, L2/L3, L3/L4, L4/L5, and L5/S1. Higher-intensity cells indicate stronger class-balanced severity classification. The anatomical illustration provides the corresponding cranio-caudal location of each intervertebral level.

To complement the tabulated results, Figure 15 visualizes the spinal-level validation Macro-F1 profiles of all eight model configurations. Macro-F1 is particularly informative in this analysis because it assigns equal importance to the three severity classes and therefore highlights level-specific weaknesses that may remain hidden by overall accuracy in the presence of class imbalance.

The heatmap reveals a clear anatomical pattern among the 2D architectures. For ViT, MaxViT, ConvNeXt, and EfficientNet, Macro-F1 generally increases from the upper lumbar levels toward L3/L4 and L4/L5. All four 2D models reach their highest Macro-F1 at L4/L5, with values of 0.608, 0.696, 0.682, and 0.676, respectively. This indicates that the lower-middle lumbar region provides the strongest balanced discrimination among the three severity classes for the conventional 2D pipelines.

The uppermost L1/L2 level presents the opposite pattern. Macro-F1 reaches 0.473 for ViT, 0.387 for MaxViT, 0.478 for ConvNeXt, and 0.394 for EfficientNet, representing the weakest spinal-level Macro-F1 for all four 2D architectures. Importantly, these comparatively modest values should not be interpreted in isolation as poor overall prediction accuracy. Macro-F1 gives equal weight to the Normal/Mild, Moderate, and Severe classes and is therefore sensitive to weak discrimination of underrepresented severity categories. Consequently, a spinal level may exhibit high overall accuracy while still obtaining a substantially lower Macro-F1 if predictions are dominated by the majority severity class.

The 2.5D-to-3D models show a more heterogeneous profile. MaxViT clearly provides the strongest stacked-input behavior across the lumbar spine, increasing from a Macro-F1 of 0.412 at L1/L2 to 0.608 at L4/L5 before decreasing to 0.512 at L5/S1. The 2.5D-to-3D ViT and EfficientNet models show a similar, although substantially weaker, tendency toward improved balanced classification at L4/L5.

In contrast, the 2.5D-to-3D ConvNeXt model exhibits the reverse trend. Its Macro-F1 decreases progressively from 0.324 at L1/L2 to 0.242 at L4/L5, followed by only a modest recovery to 0.281 at L5/S1. This pattern further confirms that the stacked-input representation does not provide a uniform benefit across architecture families and that anatomical performance must be interpreted jointly with model design.

A direct comparison between the two panels also shows that the 2D representations generally achieve higher spinal-level Macro-F1 than their 2.5D-to-3D counterparts. The difference is particularly evident for ConvNeXt, whereas MaxViT preserves the largest proportion of its 2D performance after transition to the stacked 2.5D-to-3D representation.

Overall, Figure 15 demonstrates that balanced severity classification is anatomically dependent and that the relative difficulty of individual spinal levels differs between model architectures and input representations. The concentration of higher Macro-F1 values around L3/L4–L4/L5 for most configurations contrasts with the lower balanced performance observed at L1/L2 and, in several cases, L5/S1. These findings reinforce the value of spinal-level decomposition, since a single global Macro-F1 would obscure where along the lumbar spine the model performs reliably and where class-balanced discrimination becomes more difficult.

#### 4.4.5. Anatomically Grounded Multi-Metric Performance Overlay

**Figure 16:**
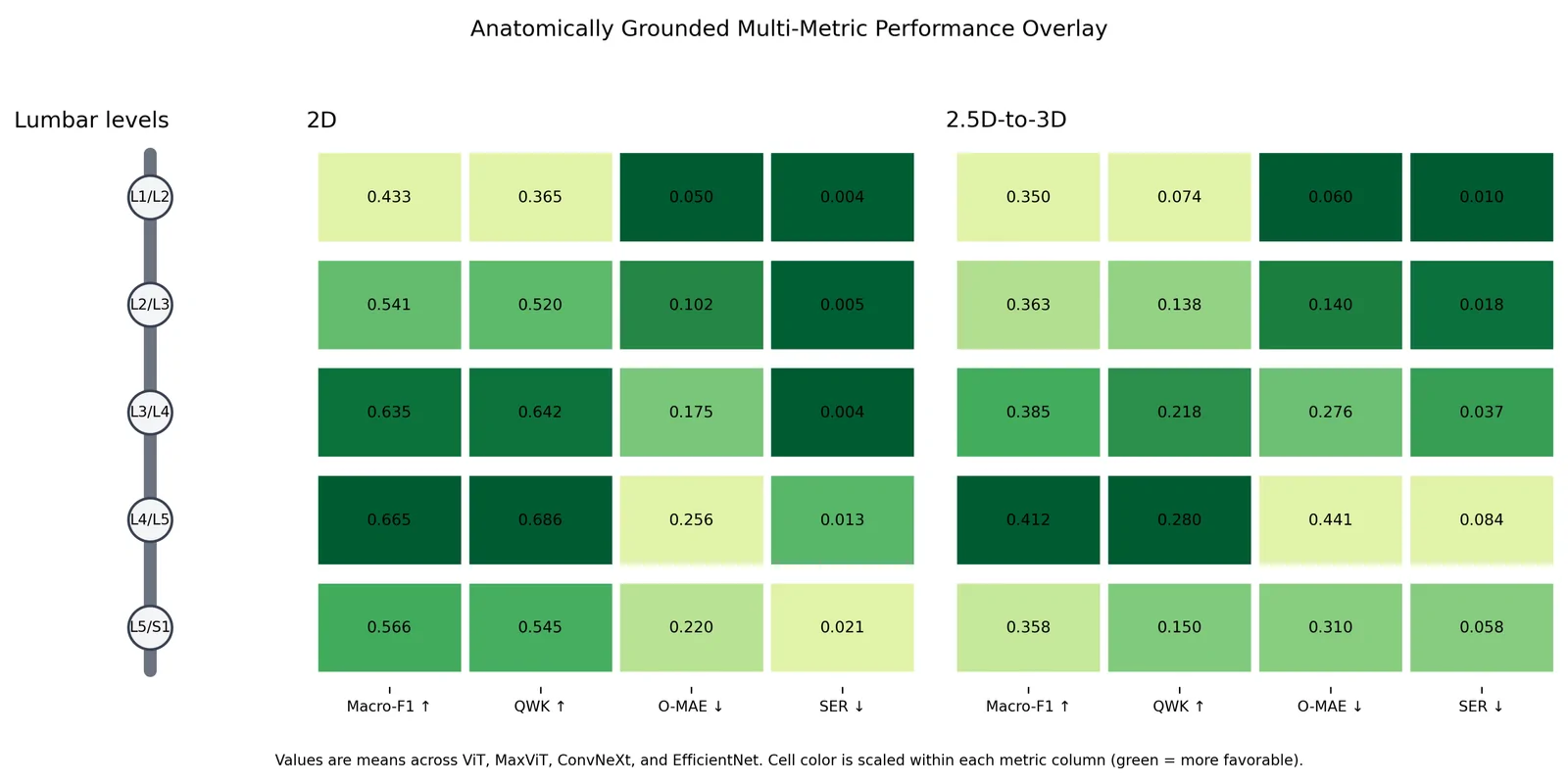
Anatomically grounded spinal-level performance comparison between 2D and 2.5D-to-3D representations. Values at each intervertebral level are averaged across ViT, MaxViT, ConvNeXt, and EfficientNet architectures. The color-coded elements distinguish the two input representations, as identified in the figure legend, enabling direct comparison of their performance at each lumbar level. The upward arrows (↑) indicate metrics for which higher values are preferable (Macro-F1 and QWK), whereas the downward arrows (↓) indicate metrics for which lower values are preferable (O-MAE and SER). The anatomical layout illustrates variations in classification performance, ordinal agreement, average severity displacement, and severe two-grade errors along the cranio-caudal lumbar axis.

While the preceding Macro-F1 heatmap emphasizes architecture-specific differences across spinal levels, Figure 16 provides a complementary anatomically grounded view of the results. Instead of displaying individual architecture rows, the figure aggregates the four backbone models within each representation and maps the resulting mean performance directly onto the five lumbar intervertebral levels. This allows exact class-balanced performance and ordinal error behavior to be interpreted simultaneously in their anatomical context.

The 2D representation exhibits a clear cranio-caudal evolution in balanced classification and ordinal agreement. Mean Macro-F1 increases from 0.433 at L1/L2 to 0.541 at L2/L3 and 0.635 at L3/L4, reaching its maximum of 0.665 at L4/L5 before decreasing to 0.566 at L5/S1. QWK follows an analogous pattern, increasing from 0.365 at L1/L2 to 0.686 at L4/L5 and subsequently decreasing to 0.545 at L5/S1. Therefore, when averaged across the four architectures, the strongest balanced and ordinal discrimination is observed around the middle-to-lower lumbar region, particularly at L4/L5.

However, the error-oriented metrics reveal a complementary pattern. For the 2D models, O-MAE increases from only 0.050 at L1/L2 to 0.256 at L4/L5, while SER increases from 0.004 at L1/L2 to 0.013 at L4/L5 and reaches its largest value of 0.021 at L5/S1. Thus, the levels associated with stronger Macro-F1 and QWK are not necessarily those associated with the smallest ordinal error magnitude. This distinction is important because improved balanced discrimination does not imply that the remaining errors are clinically less consequential.

The 2.5D-to-3D representation shows the same general anatomical tendency but at substantially weaker absolute performance. Mean Macro-F1 increases from 0.350 at L1/L2 to 0.408 at L4/L5, while QWK rises from 0.072 to 0.273 over the same interval. In parallel, O-MAE increases markedly from 0.060 to 0.444 and SER from 0.010 to 0.086. At L5/S1, Macro-F1 and QWK decrease to 0.356 and 0.146, respectively, while O-MAE and SER remain comparatively high at 0.309 and 0.058.

The anatomical overlay therefore highlights an important Beyond-Accuracy observation: the definition of the “best” spinal level depends on the metric being considered. L4/L5 is generally the strongest level for balanced classification and ordinal agreement, yet it is simultaneously associated with considerably larger severity displacement when predictions are incorrect. Conversely, L1/L2 exhibits weaker Macro-F1 and QWK but the lowest O-MAE and SER values in both representations.

This multi-metric anatomical view complements the architecture-specific Macro-F1 profiles by showing that spinal-level difficulty is multi-dimensional. Rather than asking only where classification performance is highest, the overlay simultaneously indicates where predictions are balanced, where ordinal agreement is strongest, and where the most consequential severity errors tend to occur. Consequently, the figure provides a clinically more intuitive summary of how predictive behavior changes along the lumbar spine.

#### 4.4.6. Spinal-Level Profile Plots

**Figure 17:**
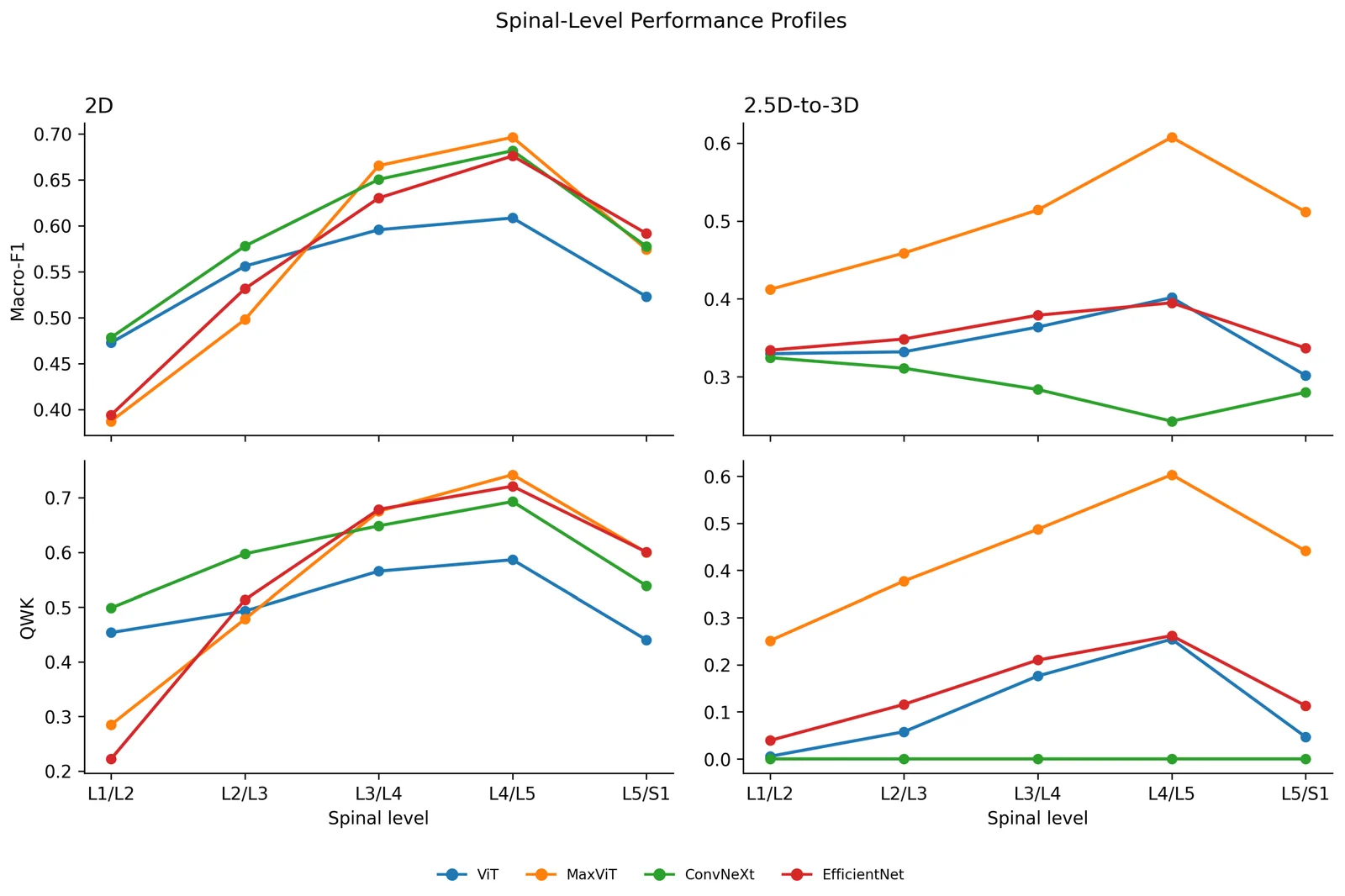
Spinal-level validation performance profiles across the five lumbar levels (L1/L2 to L5/S1). The upper panels summarize Macro-F1 profiles, while the lower panels summarize Quadratic Weighted Kappa (QWK) profiles. Separate panels are shown for 2D and 2.5D-to-3D models, enabling direct comparison of anatomical performance trends across ViT, MaxViT, ConvNeXt, and EfficientNet.

To complement the spinal-level tables with a more intuitive view of anatomical trends, Figure 17 summarizes the level-wise validation performance profiles across the five lumbar levels, from L1/L2 to L5/S1. The figure reports one ordered profile per architecture and representation, allowing direct visual comparison of how model behavior evolves along the lumbar spine. Because spinal level is a naturally ordered anatomical axis, line-based profiles provide a more interpretable representation than isolated bar charts.

The main advantage of this representation is that it reveals whether a model behaves consistently across spinal levels or whether its performance is concentrated at a subset of anatomical locations. In this sense, the figure addresses a key Beyond-Accuracy question: whether a model that appears competitive on average also remains stable from the upper lumbar spine to the lower lumbar spine.

The Macro-F1 profiles in the upper panels highlight the balanced classification behavior at each spinal level. This is particularly important in the present three-class setting, because a model may achieve acceptable overall accuracy while still performing unevenly across levels or favoring the majority severity category at specific locations. The QWK profiles in the lower panels provide a complementary ordinal perspective by emphasizing how well predictions preserve the ordered severity structure, even when exact class matches are not always achieved.

Taken together, these spinal-level profile plots provide a compact anatomical summary of model behavior. They help identify level-specific strengths and weaknesses, reveal whether performance degradation is localized or systematic, and support direct comparison between 2D and 2.5D-to-3D pipelines. In this way, the figure complements the tabulated spinal-level metrics by showing not only *how well* the models perform, but also *where* along the lumbar spine their performance is more or less reliable.

#### 4.4.7. Best–Worst Spinal-Level Bottleneck Analysis

**Figure 18:**
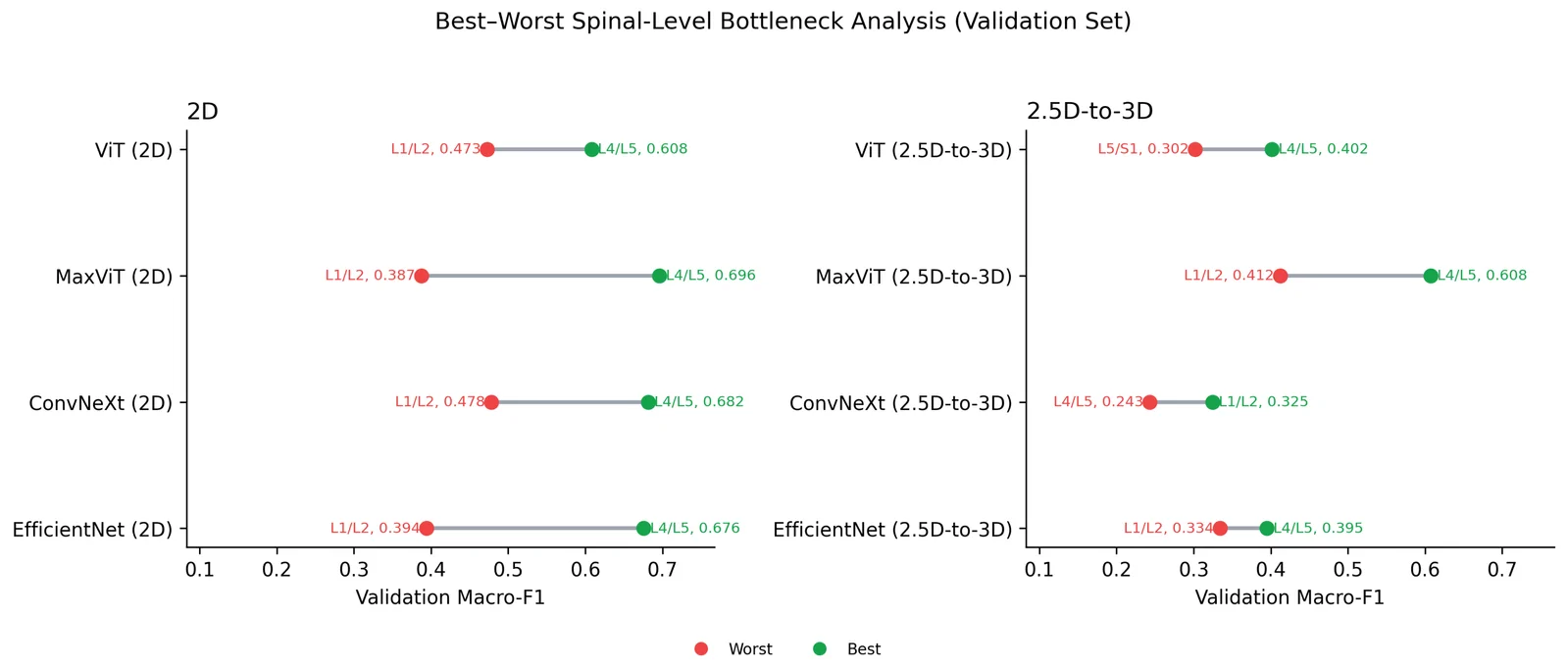
Best–worst spinal-level validation Macro-F1 comparison across the evaluated architectures and input representations. For each model, the lowest and highest spinal-level Macro-F1 values are shown together with their corresponding anatomical levels. The connecting segment represents the best–worst performance spread $\Delta_{\mathrm{level}}$, where a larger spread indicates greater anatomical variability.

To provide a compact view of anatomical performance variability, Figure 18 summarizes, for each architecture, the spinal level with the lowest and highest validation Macro-F1. The horizontal distance between the two points represents the within-model performance spread across L1/L2–L5/S1 and therefore provides a direct measure of how strongly balanced classification performance depends on anatomical location.

A highly consistent pattern is observed for the 2D models. All four architectures achieve their lowest Macro-F1 at L1/L2 and their highest value at L4/L5. ViT increases from 0.473 at L1/L2 to 0.608 at L4/L5, corresponding to a spread of $\Delta_{\mathrm{level}}=0.136$. ConvNeXt follows a similar pattern, increasing from 0.478 to 0.682 ($\Delta_{\mathrm{level}}=0.203$).

The largest anatomical variation among the 2D models is observed for MaxViT, whose Macro-F1 increases from 0.387 at L1/L2 to 0.696 at L4/L5, yielding $\Delta_{\mathrm{level}}=0.309$. EfficientNet shows a similarly pronounced difference, from 0.394 at L1/L2 to 0.676 at L4/L5 ($\Delta_{\mathrm{level}}=0.282$). These results indicate that, although MaxViT and EfficientNet achieve strong balanced classification at L4/L5, their performance is substantially less uniform across the complete lumbar spine.

The 2.5D-to-3D models exhibit a more heterogeneous bottleneck pattern. MaxViT retains the same anatomical profile as in the 2D setting, with the lowest Macro-F1 at L1/L2 (0.412) and the highest at L4/L5 (0.608), corresponding to a spread of 0.195. In contrast, ViT reaches its minimum at L5/S1 (0.302) and its maximum at L4/L5 (0.402), resulting in a narrower spread of 0.100.

EfficientNet shows the smallest best–worst difference among all evaluated pipelines. Its Macro-F1 ranges from 0.328 at L5/S1 to 0.380 at L4/L5, yielding $\Delta_{\mathrm{level}}=0.051$. However, this comparatively small spread should not be interpreted as superior robustness in isolation, because the model remains within a relatively low absolute Macro-F1 range across all five levels.

The 2.5D-to-3D ConvNeXt configuration exhibits a distinct and reversed anatomical pattern. Its highest Macro-F1 occurs at L1/L2 (0.324), whereas its lowest value is observed at L4/L5 (0.242), producing a spread of 0.082. This contrasts with the remaining architectures, for which L4/L5 generally constitutes the strongest spinal level. The finding further illustrates the architecture-dependent behavior of the stacked-input representation.

Overall, Figure 18 demonstrates that anatomical variability cannot be inferred from an overall or mean Macro-F1 alone. A model may achieve a high peak value while simultaneously exhibiting a large performance drop at another spinal level. Conversely, a narrow best–worst spread may simply indicate uniformly modest performance. Therefore, both the absolute Macro-F1 values and $\Delta_{\mathrm{level}}$ should be considered jointly when evaluating spinal-level consistency.

The analysis also reveals an important distinction between the two input representations. The 2D pipelines show a remarkably consistent anatomical pattern, with L1/L2 acting as the principal balanced-classification bottleneck and L4/L5 as the strongest level. The 2.5D-to-3D models display greater architecture-specific variation, with bottlenecks occurring at L1/L2, L4/L5, or L5/S1 depending on the backbone. This result further supports the need for anatomically resolved evaluation within the proposed Beyond-Accuracy framework.

### 4.5. Patient-Level Evaluation Results

The final dimension of the Beyond-Accuracy framework evaluates whether prediction errors remain isolated at the level of individual findings or accumulate within the complete assessment of the same patient. For each patient, all valid condition–level predictions were aggregated and summarized using the patient-level exact-match rate, patient O-MAE, and the maximum ordinal error. Patients were subsequently partitioned into three mutually exclusive error profiles: fully exact ($R_{0}$), adjacent-only ($R_{1}$), and severe-error ($R_{2}$), where $R_{2}$ denotes the presence of at least one direct Normal/Mild–Severe disagreement.

Table 6 summarizes the validation-set patient-level results across all eight architecture–representation configurations. Values are reported as mean ± standard deviation across five repeated runs. The patient-macro exact-match rate represents the mean fraction of correct condition–level predictions per patient, whereas $R_{0}$ is more stringent and represents the proportion of patients for whom *all* available predictions were correct.

The patient-level results reveal a clear advantage for the 2D pipelines. Among these models, ConvNeXt achieves the highest patient-macro exact-match rate (0.858) and the lowest patient O-MAE (0.149), indicating both a larger proportion of correct predictions within each patient assessment and a smaller average ordinal displacement when errors occur. ConvNeXt also achieves the largest proportion of completely correct patient profiles, $R_{0}=0.485$, meaning that approximately 48.5% of evaluated patients have no prediction errors across their available condition–level assessments.

MaxViT provides a closely competitive patient-level profile. Its patient-macro exact-match rate reaches 0.853, with a patient O-MAE of 0.154. Importantly, MaxViT produces the lowest severe-error patient rate among the 2D models, with only $R_{2}=0.043$. Thus, although ConvNeXt provides slightly stronger overall patient-level exactness, MaxViT minimizes the proportion of patients experiencing at least one maximum-distance severity error.

The distinction between Patient Exact and $R_{0}$ is particularly informative. For example, the 2D ConvNeXt model correctly predicts approximately 85.8% of the available findings for the average patient, yet only 48.5% of patients have a completely error-free assessment. Similarly, MaxViT achieves a patient-macro exact-match rate of 85.3%, while 45.8% of patients are fully exact. This demonstrates that a high average fraction of correct predictions does not imply that the majority of patients receive a completely error-free structured assessment.

The error-profile decomposition further clarifies the nature of the remaining errors. Across the 2D architectures, approximately 44–49% of patients are fully exact, while approximately 46–51% experience only adjacent-grade errors. Severe patient-level errors remain comparatively uncommon, ranging from 4.3% for MaxViT to 8.4% for ViT. Therefore, most imperfect 2D patient profiles are characterized by limited one-grade disagreements rather than direct Normal/Mild–Severe failures.

The 2.5D-to-3D pipelines show substantially weaker patient-level behavior. MaxViT remains the strongest configuration in this group, achieving the highest patient-macro exact-match rate (0.826), the lowest patient O-MAE (0.191), and the lowest severe-error patient rate ($R_{2}=0.097$). It is also the only 2.5D-to-3D architecture for which the severe-error patient rate remains below 10%.

In contrast, the 2.5D-to-3D ViT, ConvNeXt, and EfficientNet models exhibit considerably larger severe-error burdens. ViT produces severe errors in approximately 22.1% of patients, EfficientNet in 18.9%, and ConvNeXt in 25.9%. The corresponding patient O-MAE values of 0.258, 0.239, and 0.284 confirm that these models not only make more patient-level errors but also produce larger average severity displacement.

The contrast is especially pronounced for 2.5D-to-3D ConvNeXt. Although approximately 39.0% of patients remain completely exact, 25.9% experience at least one maximum-distance severity error. This configuration also shows the lowest patient-macro exact-match rate among all evaluated models (0.778) and the largest patient O-MAE (0.284). These findings are consistent with the weak ordinal and spinal-level behavior observed for the same architecture in the preceding analyses.

Overall, the patient-level results reinforce the conclusions obtained from the condition-specific and spinal-level evaluations. The strongest 2D pipelines combine high within-patient exactness with low ordinal error and a relatively small proportion of severe patient-level failures. By contrast, several 2.5D-to-3D configurations exhibit substantially higher severe-error patient rates despite retaining apparently high overall exact-match proportions.

This distinction illustrates the central motivation of the Beyond-Accuracy framework: two models may appear broadly comparable when assessed using a single pooled metric, yet differ substantially in how prediction errors are distributed across individual patients. Patient-level exact-match rate, patient O-MAE, and the $R_{0}/R_{1}/R_{2}$ error-profile distribution therefore provide complementary information about the reliability and clinical severity of complete structured patient assessments.

#### 4.5.1. Patient Error-Profile Distribution

**Figure 19:**
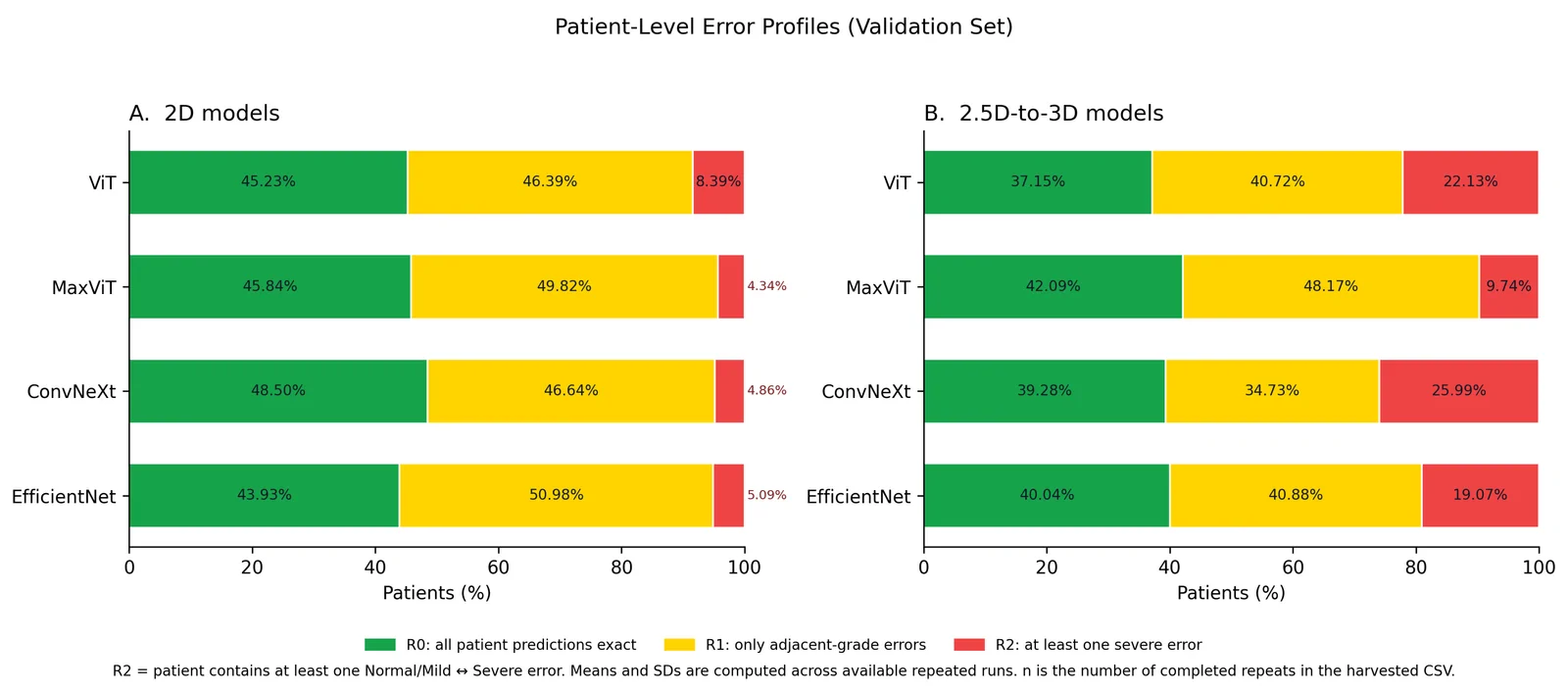
Validation-set patient-level error-profile distributions for the 2D and 2.5D-to-3D models. Each horizontal bar represents the proportion of patients assigned to one of three mutually exclusive categories according to their maximum ordinal error: $R_{0}$, all patient predictions exact; $R_{1}$, adjacent-grade errors only; and $R_{2}$, at least one severe Normal/Mild–Severe error. Percentages are averaged across repeated runs.

Beyond the patient-macro exact-match rate and patient-level O-MAE, the distribution of maximum ordinal errors provides a more clinically interpretable view of how prediction failures affect individual patients. Figure 19 summarizes the validation-set patient error profiles for all eight architecture–representation combinations using three mutually exclusive categories: $R_{0}$, corresponding to patients for whom all available predictions are exact; $R_{1}$, corresponding to patients affected only by adjacent-grade errors; and $R_{2}$, corresponding to patients with at least one direct Normal/Mild–Severe disagreement.

The 2D models exhibit a markedly more favorable patient-level error profile. ConvNeXt achieves the largest proportion of completely correct patient assessments, with $R_{0}=48.50\%$, followed by MaxViT at 45.84%, ViT at 45.23%, and EfficientNet at 43.93%. Thus, for the strongest 2D configuration, almost one half of the evaluated patients receive no erroneous condition–level predictions across their complete available assessment.

For patients who are not completely error free, the majority of errors in the 2D setting remain confined to adjacent severity grades. The $R_{1}$ proportion ranges from 46.39% for ViT to 50.98% for EfficientNet. More importantly, severe patient-level failures remain uncommon. MaxViT produces the lowest $R_{2}$ rate at only 4.34%, followed closely by ConvNeXt (4.86%) and EfficientNet (5.09%), while ViT reaches 8.39%. These results indicate that the dominant error mode of the 2D pipelines is a one-grade disagreement rather than a maximum-distance severity error.

The 2.5D-to-3D models display a substantially different pattern. MaxViT again provides the strongest profile within this representation, with $R_{0}=42.09\%$, $R_{1}=48.17\%$, and $R_{2}=9.74\%$. Although its proportion of completely exact patients is lower than that of the strongest 2D models, the severe error rate remains below 10%, distinguishing MaxViT from the other stacked-input architectures.

In contrast, severe patient-level errors become considerably more frequent for the remaining 2.5D-to-3D models. ViT produces at least one severe error for 22.13% of patients, EfficientNet for 18.93%, and ConvNeXt for 25.90%. The 2.5D-to-3D ConvNeXt configuration is particularly notable: although 39.00% of patients remain completely exact, more than one quarter experience at least one maximum-distance ordinal error. This behavior is consistent with the weak ordinal agreement and elevated error severity observed for the same configuration in the preceding condition- and spinal-level analyses.

The stacked distributions therefore reveal information that is not apparent from patient-macro exact-match rate alone. For example, two models can achieve similar proportions of completely correct patients while differing substantially in the type of errors experienced by the remaining subjects. A model whose imperfect assessments are dominated by $R_{1}$ patients produces primarily neighboring-grade disagreements, whereas a larger $R_{2}$ component indicates that a greater fraction of patients is exposed to at least one clinically more consequential two-grade misclassification.

Overall, Figure 19 shows that the 2D pipelines provide not only higher patient-level exactness but also substantially lower severe-error burden. Among the evaluated configurations, 2D MaxViT achieves the lowest severe-error patient rate, while 2D ConvNeXt produces the largest fraction of fully exact patient assessments. Within the 2.5D-to-3D group, MaxViT remains the most favorable configuration, whereas ConvNeXt exhibits the largest proportion of severe-error patients. These differences further support the Beyond-Accuracy premise that patient-level reliability should be characterized through both the frequency and the severity of prediction errors rather than through a single aggregate accuracy measure.

#### 4.5.2. Patient Exactness–Ordinal Error Trade-Off

**Figure 20:**
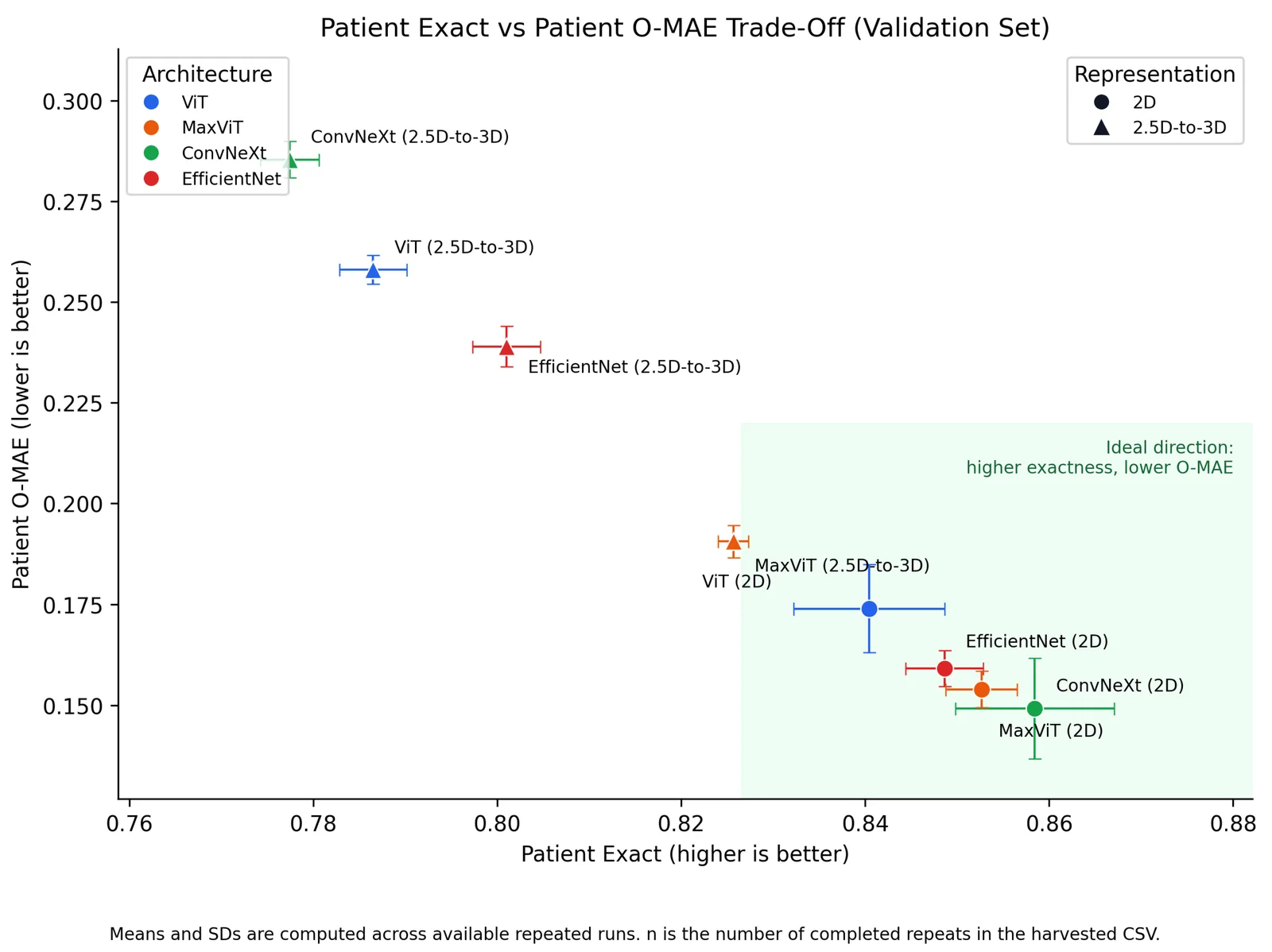
Trade-off between patient-macro exact-match rate and patient-level ordinal mean absolute error (O-MAE) on the validation set. Each point represents one architecture–representation configuration. Horizontal and vertical error bars denote variability across repeated runs. Higher patient-level exactness and lower O-MAE are desirable; therefore, the bottom-right region represents the most favorable combination of exact prediction frequency and ordinal error magnitude.

The stacked patient error-profile analysis characterizes how patients are distributed across exact, adjacent-only, and severe-error categories. A complementary perspective is obtained by jointly considering the average fraction of correct predictions within each patient and the average magnitude of ordinal disagreement. Figure 20 therefore positions each architecture–representation configuration according to its validation-set patient-macro exact-match rate and patient-level O-MAE.

The separation between the 2D and 2.5D-to-3D configurations is immediately visible. All four 2D models occupy the lower-right region of the plot, indicating simultaneously higher patient-level exactness and lower ordinal error. Among these models, ConvNeXt provides the most favorable numerical combination, achieving the highest patient-macro exact-match rate (0.858) together with the lowest patient O-MAE (0.149). MaxViT is positioned very close to this optimum, with an exact-match rate of 0.853 and O-MAE of 0.154. EfficientNet also remains within the favorable lower-right cluster, reaching 0.849 and 0.159, respectively.

The 2D ViT configuration is slightly separated from the other 2D models, with a lower patient exact-match rate of 0.840 and a higher O-MAE of 0.174. Nevertheless, it remains substantially closer to the desirable region than most of the 2.5D-to-3D configurations. This reinforces the general observation that the 2D pipelines preserve both greater exactness and smaller severity displacement at the patient level.

Within the 2.5D-to-3D group, MaxViT is clearly the strongest configuration. Its patient exact-match rate of 0.826 and patient O-MAE of 0.191 place it considerably closer to the 2D cluster than the remaining stacked-input models. This finding is consistent with the preceding condition-specific and spinal-level analyses, in which MaxViT also preserved the strongest performance after transition from 2D to 2.5D-to-3D processing.

The remaining 2.5D-to-3D models occupy progressively less favorable regions of the trade-off space. EfficientNet reaches a patient exact-match rate of 0.800 with an O-MAE of 0.239, while ViT decreases to 0.786 with an O-MAE of 0.258. The weakest configuration is 2.5D-to-3D ConvNeXt, which combines the lowest patient exactness (0.778) with the largest patient O-MAE (0.284). Its position in the upper-left portion of the plot indicates that its patient-level errors are both more frequent and, on average, farther from the reference severity grades.

The error bars further indicate that the observed separation is not solely a consequence of individual repeated runs. Although some variability is present, particularly for the 2D ConvNeXt and ViT configurations, the representation- level clustering remains clear: the 2D models occupy a distinctly more favorable region than the corresponding 2.5D-to-3D pipelines.

Figure 20 also illustrates why the two patient-level metrics should be interpreted jointly. Patient exact-match rate describes *how frequently* the component predictions of a patient’s assessment are correct, whereas O-MAE describes *how large* the remaining severity disagreements are. A model can therefore be considered more favorable only when it simultaneously moves toward higher exactness and lower ordinal displacement.

This two-dimensional view provides a concise summary of the patient-level Beyond-Accuracy analysis. The strongest configurations are not simply those with the greatest number of correct predictions, but those that also limit the severity of their remaining errors. In the present experiments, 2D ConvNeXt and 2D MaxViT provide the strongest overall patient-level trade-off, while 2.5D-to-3D MaxViT represents the most competitive stacked-input alternative.

#### 4.5.3. Severe-Error Patient Burden

**Figure 21:**
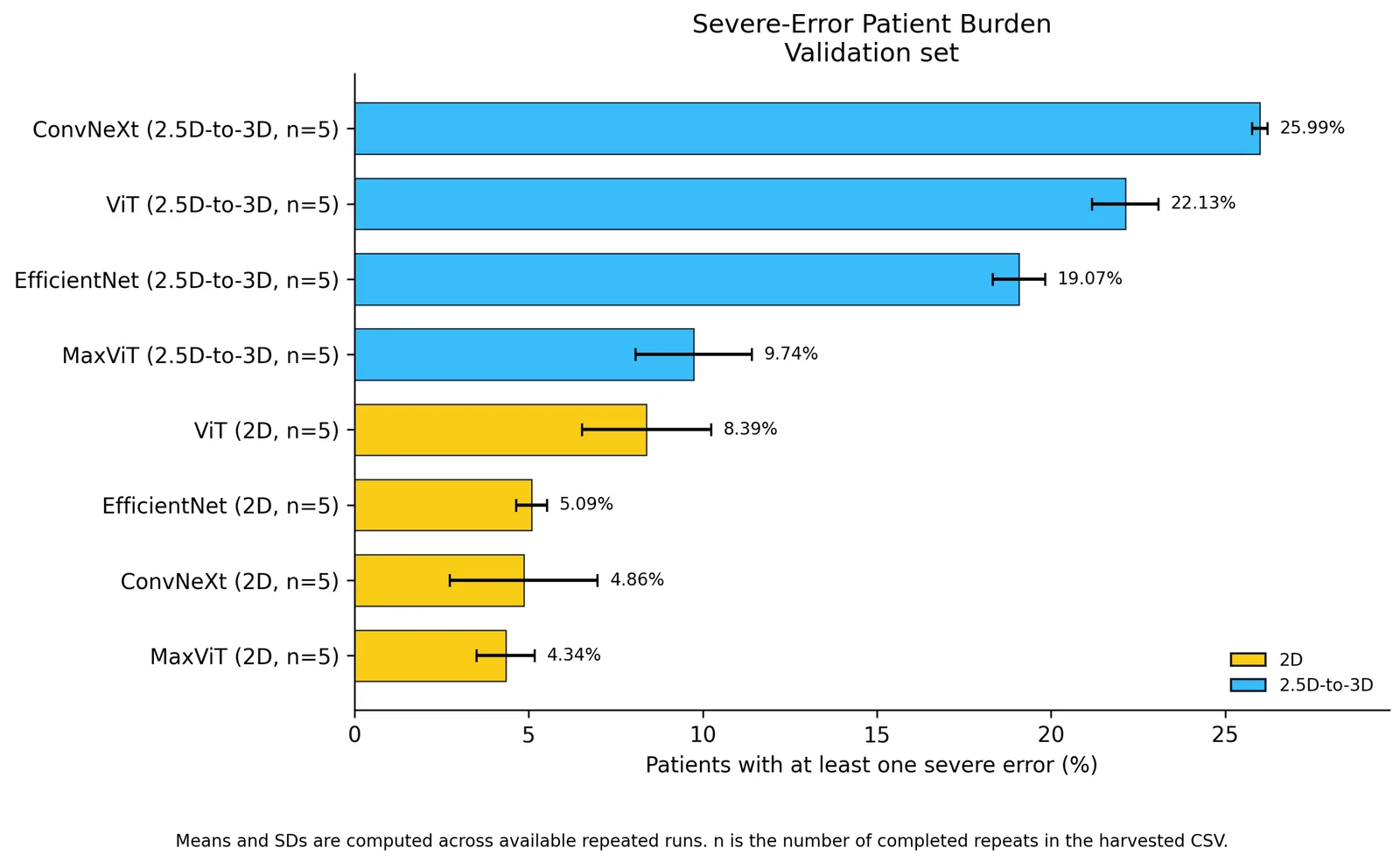
Validation-set severe-error patient burden across architectures and input representations. Each bar shows the proportion of patients ($R_{2}$) who received at least one maximum-distance ordinal error, i.e., at least one Normal/Mild ↔ Severe misclassification. Lower values are better. Error bars denote the standard deviation across repeated runs.

Figure 21 summarizes the proportion of patients who received at least one maximum-distance ordinal error, denoted by $R_{2}$. In the present three-class formulation, a severe patient-level error corresponds to at least one direct Normal/Mild ↔ Severe misclassification within the full set of predictions associated with that patient. This measure provides a clinically intuitive complement to mean patient-level metrics because it highlights how often a model produces at least one clearly unacceptable ordinal mistake for an individual subject.

As shown in Figure 21, the 2D models generally achieve a lower severe-error patient burden than their 2.5D-to-3D counterparts, indicating that they more rarely produce clinically serious far-apart mistakes at the patient level. Among the strongest configurations, the 2D models cluster toward the lower end of the chart, suggesting that their advantages are not limited to higher exact agreement but also extend to safer ordinal behavior across complete patient profiles.

In contrast, several 2.5D-to-3D configurations exhibit larger $R_{2}$ values, indicating that a greater fraction of patients experience at least one maximum-distance severity error. This finding is particularly relevant because moderate aggregate accuracy may conceal clinically significant patient-level errors, potentially limiting the reliability of radiologist-oriented decision-support applications. Therefore, $R_{2}$ complements patient-level exact-match metrics by quantifying the proportion of patients affected by severe ordinal misclassification.

Overall, the severe-error burden analysis reinforces the Beyond-Accuracy perspective of this study: models should not be evaluated only by how often they are correct on average, but also by how often they expose patients to large ordinal mistakes. In this sense, lower $R_{2}$ values indicate not only better ordinal consistency, but also a more favorable safety profile for structured lumbar degeneration assessment.

### 4.6. Illustrative Patient-Level Case Study

To complement the cohort-level patient analysis with an interpretable individual example, one complete lumbar MRI examination was evaluated across all five degenerative conditions and all five lumbar levels. The resulting patient profile therefore comprised

(33) 5conditions×5lumbarlevels=25

condition–level severity assessments. This case study illustrates how the condition-specific models can be combined to produce a structured patient-level representation rather than a collection of isolated predictions.

The examination included sagittal assessments for spinal canal stenosis (SCS), left neural foraminal narrowing (LFN), and right neural foraminal narrowing (RFN), together with axial assessments for left and right subarticular stenosis (LSS and RSS). For every condition–level combination, the complete MRI slice, the expert-coordinate-centered 64×64 ROI, the reference severity, predicted severity, class probabilities, and predictive entropy were retained.

#### 4.6.1. Sagittal Condition Profiles

**Figure 22:**
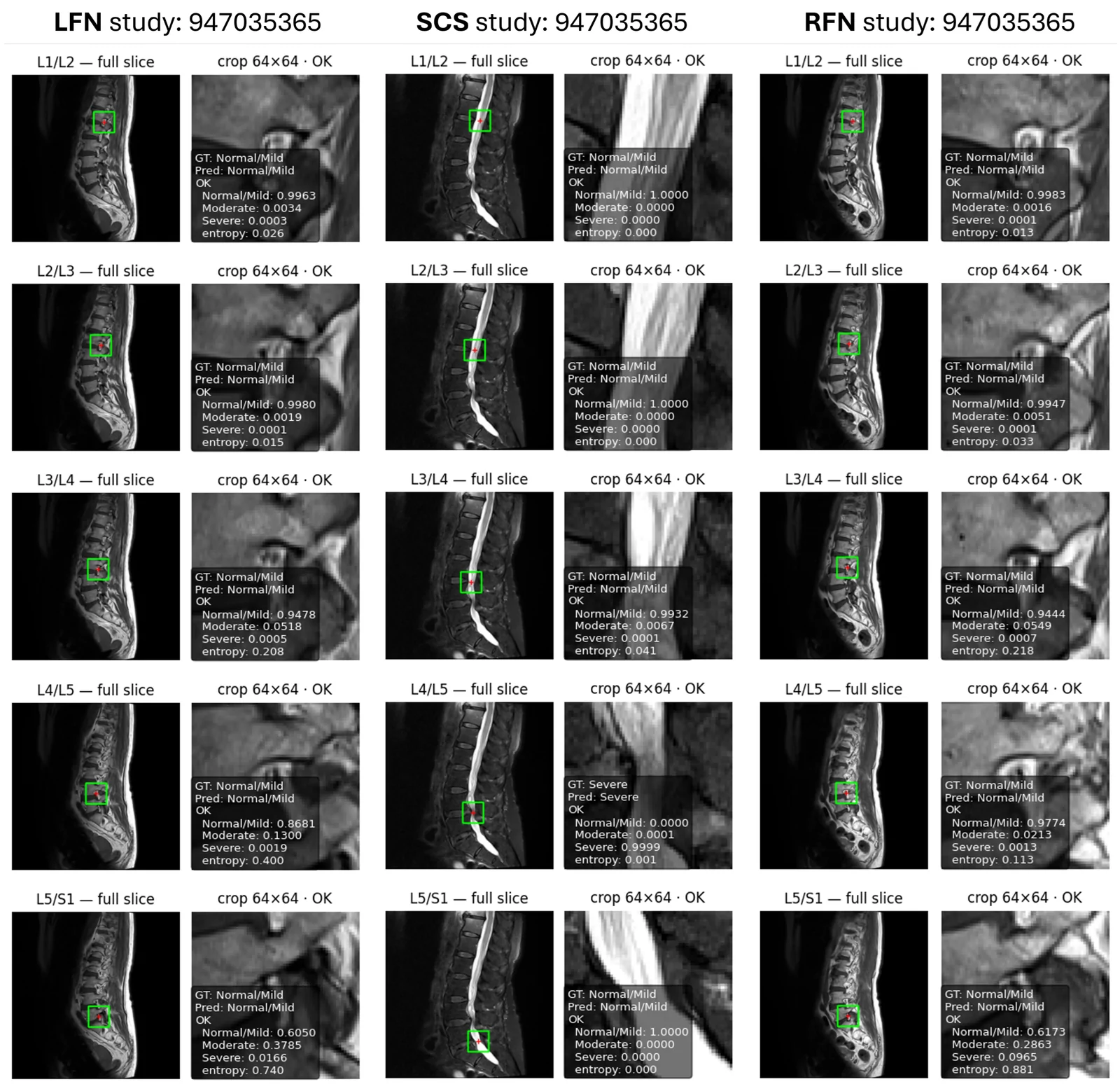
Patient-l evel sagittal evaluation for SCS, LFN, and RFN across the five lumbar levels. For each condition–level assessment, the complete MRI slice is displayed together with the expert-coordinate-centered 64×64 ROI. The overlays report the ground-truth severity, predicted severity, class-probability distribution, and predictive entropy. All 15 predictions shown in this figure were classified correctly.

Figure 22 presents the SCS, LFN, and RFN predictions across L1/L2–L5/S1. All 15 sagittal condition–level assessments were classified correctly, although the corresponding probability distributions reveal substantial variation in predictive confidence.

For SCS, all five lumbar levels were labeled and correctly predicted as Normal/Mild. Confidence was particularly high at L1/L2 and L2/L3, where the Normal/Mild probabilities reached 0.9963 and 0.9980, respectively. Nevertheless, confidence decreased toward the lower lumbar spine. At L4/L5, the probability assigned to Normal/Mild decreased to 0.8681, while the Moderate class received 0.1300. The largest SCS uncertainty occurred at L5/S1, where the Normal/Mild and Moderate probabilities were 0.6050 and 0.3785, respectively, with an entropy of 0.740.

The LFN profile contained a strongly localized severe abnormality. L1/L2, L2/L3, L3/L4, and L5/S1 were correctly classified as Normal/Mild, whereas L4/L5 was labeled and predicted as Severe. At this level, the probability assigned to the Severe class reached 0.9999, with an entropy of only 0.001. The patient-level representation therefore preserves the fact that severe degeneration may be highly localized even when the remaining levels for the same condition appear Normal/Mild.

RFN was also correctly classified as Normal/Mild at all five levels. Predictions remained highly confident through L4/L5; however, uncertainty increased substantially at L5/S1. At this level, the model assigned probabilities of 0.6173, 0.2863, and 0.0965 to Normal/Mild, Moderate, and Severe, respectively, producing an entropy of 0.881. Thus, the final class remained correct despite a considerably less concentrated probability distribution.

These examples demonstrate why prediction confidence can provide information that is not visible from the final hard class alone. However, elevated entropy should not be interpreted as evidence that a case is necessarily radiologically borderline. Such uncertainty may also arise from local anatomical variability, limited ROI context, image appearance, class imbalance, annotation uncertainty, or model-specific limitations.

#### 4.6.2. Axial Condition Profiles and the Patient-Level Error

**Figure 23:**
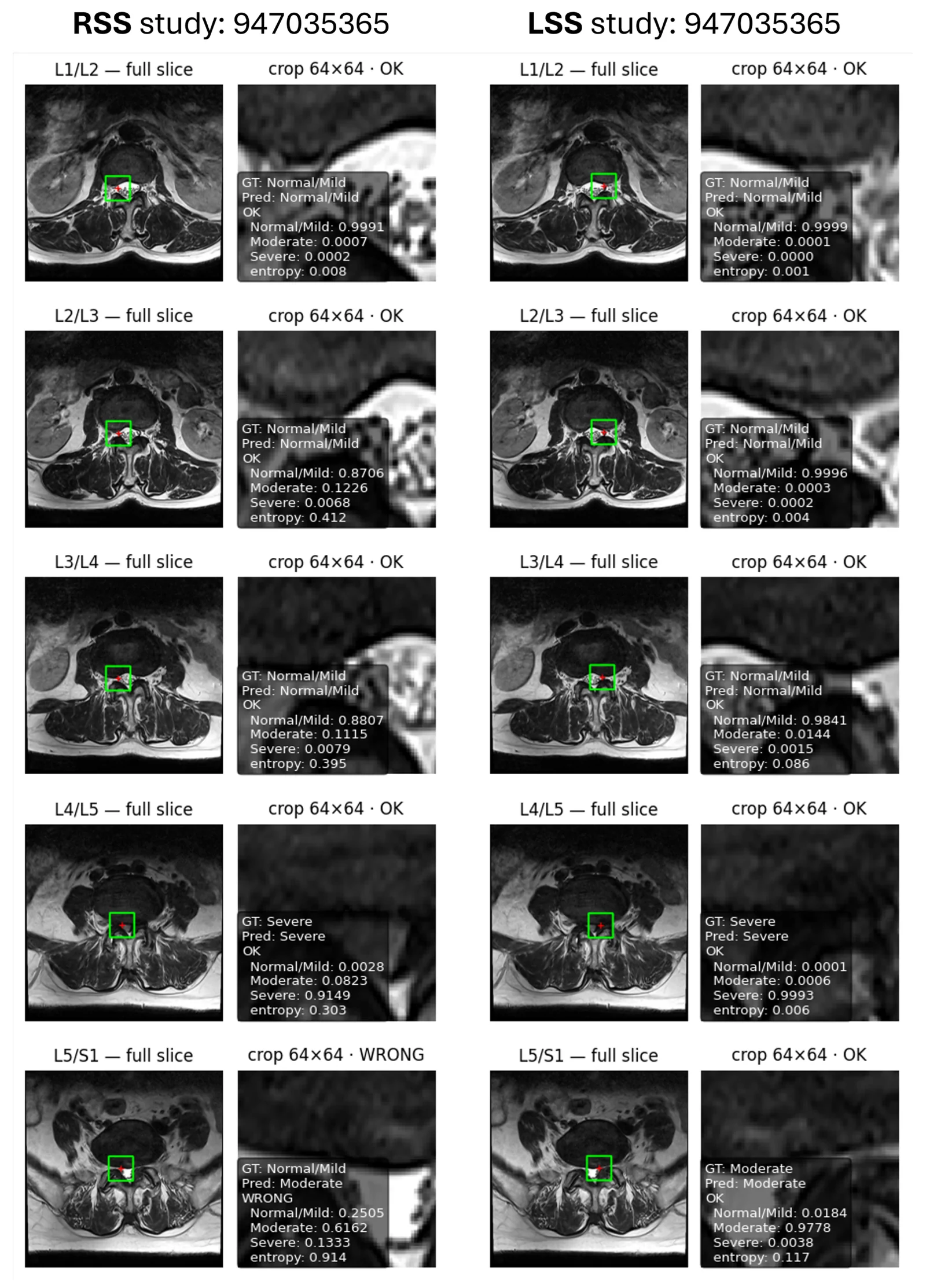
Patient-level axial evaluation for RSS and LSS across the five lumbar levels. Each assessment includes the complete axial MRI slice, the expert-coordinate-centered 64×64 ROI, ground-truth severity, predicted severity, class probabilities, and predictive entropy. Nine of the ten predictions were correct. The only disagreement occurred for RSS at L5/S1, where a Normal/Mild reference label was predicted as Moderate.

Figure 23 extends the same analysis to RSS and LSS using axial MRI slices. Nine of the ten axial condition–level assessments were predicted correctly. The only disagreement in the complete 25-assessment patient profile occurred for RSS at L5/S1.

For RSS, the first four lumbar levels were classified correctly. L1/L2 was predicted as Normal/Mild with a probability of 0.9991. Confidence decreased at L2/L3 and L3/L4, where the Normal/Mild probabilities were 0.8706 and 0.8807, respectively. At L4/L5, the model correctly identified Severe disease with a probability of 0.9149, with most of the remaining probability mass assigned to the adjacent Moderate category.

The only incorrect prediction occurred at RSS L5/S1. The reference severity was Normal/Mild, whereas the model predicted Moderate. The probability vector was

(34) $$\left[p_{\mathrm{Normal}/\mathrm{Mild}},p_{\mathrm{Moderate}},p_{\mathrm{Severe}}\right]=\left[0.2505,\;0.6162,\;0.1333\right],$$

and the corresponding entropy reached 0.914, the largest value observed in the RSS–LSS portion of the case study. Importantly, the disagreement was an adjacent-grade error,

(35) Normal/Mild→Moderate,

rather than a maximum-distance Normal/Mild–Severe error.

The LSS model correctly classified all five levels. Normal/Mild predictions at L1/L2 and L2/L3 were obtained with probabilities of 0.9999 and 0.9996, respectively. At L4/L5, the model correctly identified Severe disease with a probability of 0.9993, while L5/S1 was correctly classified as Moderate with a probability of 0.9778. The patient therefore exhibits a heterogeneous degeneration profile that includes Normal/Mild, Moderate, and Severe findings at different anatomical locations.

#### 4.6.3. Beyond-Accuracy Summary of the Complete Patient Profile

Across both Figure 22 and Figure 23, the complete patient profile contained 25 condition–level predictions. Of these, 24 were exact, one was an adjacent-grade error, and none represented a two-grade Normal/Mild–Severe disagreement.

The patient-level exact-match rate defined in Section 3.8.5 is therefore

(36) $$A_{p}=\frac{24}{25}=0.960.$$

Because the only incorrect prediction differs from the reference by one severity grade, the patient-level ordinal mean absolute error is

(37) $${\text{O-MAE}}_{p}=\frac{24(0)+1(1)}{25}=0.040.$$

The maximum ordinal error observed across the examination is

(38) $$E_{p}^{\max}=1,$$

and the patient is consequently assigned to the *adjacent-only* error category $R_{1}$.

Table 7 summarizes the complete patient-level evaluation, including the number of exact predictions, adjacent-grade errors, severe errors, and the corresponding Beyond-Accuracy indicators.

The case therefore illustrates an important distinction between strict patient-level correctness and patient-level error severity. An exact-match rate of 0.960 indicates that only one of the 25 predictions was incorrect, while an O-MAE of 0.040 and $E_{p}^{\max}=1$ show that the disagreement remained immediately adjacent to the reference grade. Consequently, this patient does not contribute to the severe-error patient burden $R_{2}$ reported in the cohort-level analysis.

At the same time, the two detailed figures demonstrate that binary correct/incorrect labels alone do not describe the complete inference behavior. For example, the correctly classified SCS and RFN predictions at L5/S1 exhibited substantially greater uncertainty than many other correctly classified assessments. Conversely, several abnormal findings, including Severe LFN and LSS at L4/L5, were predicted with very high confidence.

Taken together, the complete examination reveals a heterogeneous anatomical disease profile: severe LFN, RSS, and LSS findings are present at L4/L5, Moderate LSS is present at L5/S1, and most remaining condition–level assessments are Normal/Mild. Such information would be obscured by a single patient-level class or a single global accuracy value.

The case study therefore provides an intuitive example of the role of the patient-level dimension within the proposed Beyond-Accuracy framework. Condition-specific models generate localized predictions, the spinal-level analysis preserves their anatomical distribution, and the patient-level aggregation determines whether the resulting examination is predominantly correct, whether its errors are ordinally small, and whether any maximum-distance severity disagreement occurs. In this example, the complete profile is characterized by high exact agreement, very low average ordinal error, and no severe patient-level error.

Nevertheless, this case should be interpreted only as an illustrative application of the trained models. A single examination cannot establish clinical reliability or determine whether uncertain predictions correspond to true radiological boundary cases. Those conclusions require systematic cohort-level evaluation and independent radiological validation.

### 4.7. Computational Cost and Training Runtime

The complete severity neural architecture search comprised 39,840 designed trials across eight architecture–representation families and five lumbar degenerative conditions. Training was parallelized across eight GPUs, with independent trials distributed evenly between devices and the five conditions processed sequentially using the same GPU pool.

Mean trial duration varied substantially by architecture because of differences in model complexity and early-stopping behavior. Across the evaluated families, the measured mean duration ranged from approximately 1.1 to 3.2 min per trial. Because all search grids were divisible by eight, the parallelization strategy reduced calendar time by approximately a factor of eight without changing the total number of GPU-hours.

Table 8 summarizes the estimated wall-clock duration of the complete designed search for each architecture family. The estimates use the measured mean trial duration for each family together with the full designed NAS grid, including configurations for which compact timing records were incomplete.

The complete search therefore required approximately 217.3 h (≈9.1 days) of eight-GPU wall-clock time, corresponding to approximately 1738 GPU-hours. The equivalent sequential execution on one GPU would require approximately 72.4 days. At an assumed cloud rental price of USD 3.70 per hour for the complete eight-GPU instance, the estimated compute cost of the severity NAS was approximately USD 804.

These estimates describe the architecture-search stage only. They do not include the separate landmark-coordinate search, confirmatory retraining of the selected configurations, or subsequent post hoc condition-, spinal-, and patient-level analyses. Launch, data-loading, and worker-join overheads are also excluded because the recorded trial durations primarily represent model training time.

## 5. Discussion

This study investigated lumbar spine degeneration assessment using a condition-specific deep-learning framework applied to the RSNA 2024 LumbarDISC dataset. Rather than evaluating the task through a single pooled classification metric, the analysis was deliberately organized around several complementary levels of model behavior: exact classification, ordinal severity agreement, condition-specific performance, spinal-level variation, and patient-level error structure. This broader evaluation strategy was motivated by the observation that conventional accuracy alone can obscure important differences in the way models distribute their errors across severity grades, anatomical levels, and degenerative conditions.

A central methodological choice was to train separate models for the five degenerative findings, spinal canal stenosis (SCS), left and right neural foraminal narrowing (LFN and RFN), and left and right subarticular stenosis (LSS and RSS), rather than forcing all targets into a single shared classifier. This condition-specific formulation allowed each model to learn from imaging characteristics and anatomical patterns more closely associated with its corresponding pathology. At the same time, the resulting predictions could still be integrated into a common evaluation framework, making it possible to compare behavior across conditions and to construct structured patient-level profiles.

The comparison among ViT, MaxViT, ConvNeXt, and EfficientNet showed that architecture choice remained important even under the same condition-specific formulation. Across the 2D experiments, ConvNeXt achieved the strongest aggregate performance on several of the evaluated metrics, while MaxViT also showed consistently competitive behavior. The differences were not uniform across all conditions, however, and the condition-specific analysis revealed that apparently similar global scores could arise from substantially different profiles. In particular, some architectures maintained relatively balanced performance across the five conditions, whereas others exhibited clear condition-dependent bottlenecks. This reinforces the value of evaluating the worst-performing condition and the between-condition performance range in addition to reporting an overall mean.

The ordinal evaluation provided a second perspective on these differences. Because the severity labels are naturally ordered, a prediction of Moderate for a Normal/Mild case is qualitatively different from a direct prediction of Severe. OA, O-MAE, QWK, and SER therefore provide information that is not captured by conventional classification accuracy alone. The results showed that some configurations with apparently acceptable exact agreement could still exhibit weak ordinal consistency. A particularly illustrative example was the combination of non-trivial OA with very low or zero QWK in selected condition–architecture settings. Such behavior indicates that exact correctness does not necessarily imply that the predicted severity structure is well aligned with the reference ordering.

The trade-off between QWK and O-MAE further clarified the relative strengths of the different configurations. The strongest 2D models occupied the region associated with high ordinal agreement and low severity displacement, whereas several 2.5D-to-3D variants were clearly separated toward lower QWK and higher O-MAE. Among the stacked-input configurations, MaxViT preserved the most competitive ordinal behavior, while ConvNeXt showed the most pronounced degradation. This pattern was also reflected in SER, where the 2D models generally maintained a low frequency of direct Normal/Mild–Severe misclassifications, whereas several 2.5D-to-3D models produced substantially larger severe-error burdens.

The weaker performance of the cross-level 2.5D-to-3D configurations should be interpreted in relation to their exploratory purpose. These models were included to investigate whether jointly processing five anatomically ordered lumbar-level ROIs could improve severity classification and to assess their behavior under the proposed Beyond-Accuracy framework, rather than to establish an optimal volumetric MRI representation.

The results indicate that stacking ROIs from distinct lumbar levels along an artificial depth dimension does not necessarily improve classification or ordinal severity assessment. One possible explanation is that this representation does not preserve the physical spacing and geometric relationships of a reconstructed MRI volume. Additional cross-level information may also introduce anatomical heterogeneity that is not beneficial for every condition or architecture. These explanations remain hypotheses, as the experiments do not isolate their individual effects. Nevertheless, the comparison demonstrates the importance of evaluating additional anatomical context empirically rather than assuming that increased input dimensionality improves performance.

This distinction is particularly relevant because the 2D and 2.5D-to-3D pipelines may differ in several factors beyond input organization, including pretraining availability, parameterization, optimization behavior, and effective model capacity. The observed performance gap should therefore not be interpreted as evidence that 2D modeling is intrinsically superior to volumetric reasoning for lumbar MRI. Instead, the results indicate that, under the present training and representation strategy, the 2D configurations were more effective and more stable. A future controlled study using matched architectures, equivalent pretraining, and standardized volumetric inputs would be required to isolate the contribution of dimensionality itself.

The spinal-level analysis added an anatomical perspective to the evaluation. Performance varied systematically across L1/L2 through L5/S1 rather than remaining constant along the lumbar column. In the 2D setting, Macro-F1 and QWK generally improved from the upper lumbar levels toward L4/L5 before declining again at L5/S1. This pattern suggests that model difficulty is not uniform across anatomical locations. At the same time, O-MAE and SER did not necessarily follow the same trend as Macro-F1, demonstrating that a spinal level associated with strong balanced classification can still exhibit a larger ordinal error magnitude or a higher severe-error burden.

The best–worst spinal-level analysis emphasized this heterogeneity further. For several 2D architectures, the strongest Macro-F1 occurred at L4/L5, whereas L1/L2 represented a common lower-performing level. The pattern was different for some 2.5D-to-3D configurations, particularly ConvNeXt, whose performance degraded substantially at the lower lumbar levels. Such variation supports the inclusion of anatomically grounded evaluation rather than assuming that a single pooled score sufficiently characterizes model behavior throughout the spine.

The patient-level analysis extended this idea by regrouping condition–level predictions according to subject. Importantly, this evaluation did not introduce a new dataset partition; it provided an additional aggregation layer on top of the existing patient-disjoint validation and test sets. The patient-level exact-match rate quantified how much of the full examination was predicted correctly, while patient O-MAE summarized the average severity displacement. The maximum ordinal error further separated patients with only adjacent-grade disagreements from those containing at least one direct Normal/Mild–Severe misclassification.

This distinction proved useful because models with similar average patient exactness could differ markedly in their severe-error burden. The patient-level error-profile analysis showed that the proportion of patients falling into the severe-error category was generally much lower for the 2D models than for the corresponding 2.5D-to-3D configurations. Among the latter, MaxViT again showed the most favorable behavior. These results demonstrate that patient-level evaluation can reveal clinically relevant differences that are not apparent from pooled condition–level metrics alone.

The illustrative patient case study provided a concrete example of this multi-level interpretation. The complete profile contained predictions across all five conditions and five lumbar levels, producing 25 condition–level assessments. Of these, 24 were predicted exactly, while the only disagreement was an adjacent-grade error rather than a direct extreme-category error. The case therefore combined high patient-level exact agreement with low ordinal error and no severe patient-level error. At the same time, the associated probability distributions showed that not all correct predictions were equally confident. This supports the broader motivation for retaining more information than the final predicted class alone.

The case study should not, however, be interpreted as evidence of clinical utility. A single patient cannot establish diagnostic reliability or confirm that uncertainty patterns correspond to genuine radiological boundary cases. The example is better understood as an illustration of how the condition-, level-, and patient-specific outputs can be integrated into a structured radiologist-oriented representation.

Another important methodological aspect of the study is the use of expert-provided coordinates to define localized regions of interest. This choice reduced the localization problem and allowed the experiments to focus on severity classification within anatomically relevant regions. It also introduces an important limitation: the reported performance characterizes classification under expert-localized ROIs and should not be interpreted as the performance of a fully automated end-to-end lumbar MRI system. A deployed system would additionally require robust automated localization and should be evaluated for sensitivity to localization errors.

The repeated-run design provides an estimate of model stability across different data partitions and stochastic training conditions. Following NAS-based configuration selection, the selected models were retrained using independent study-level split seeds and training random seeds, with performance summarized as mean ± standard deviation across completed runs. Most configurations were evaluated over five runs, whereas two 2.5D-to-3D groups contained four completed runs. These experiments characterize internal variability associated with data partitioning and model training, but do not establish external-dataset robustness.

The present evaluation framework also highlights several aspects that remain open for further investigation. Model calibration, uncertainty quality, sensitivity to ROI displacement, controlled multiplanar fusion, and matched 2D versus volumetric comparisons would further extend the Beyond-Accuracy analysis. In particular, future sagittal–axial comparisons should distinguish between the use of different planes for different conditions and true multiplanar fusion, in which equivalent information from multiple views is combined for the same prediction target.

Overall, the discussion of the results indicates that the main value of the proposed framework lies not simply in identifying the numerically strongest classifier, but in exposing how architecture, input representation, pathology, anatomical level, and patient-level error structure interact. The findings show that these dimensions can produce substantially different interpretations of the same underlying predictions and therefore motivate a more multi-dimensional evaluation of lumbar spine degeneration models than can be provided by a single accuracy value.

## 6. Conclusions

This study presented a condition-specific and multi-level evaluation framework for lumbar spine degeneration assessment using the RSNA 2024 LumbarDISC dataset. Rather than characterizing model performance through a single pooled accuracy value, the proposed Beyond-Accuracy framework combined exact classification metrics with ordinal severity analysis, condition-specific evaluation, spinal-level assessment, and patient-level aggregation.

Four architecture families, ViT, MaxViT, ConvNeXt, and EfficientNet, were evaluated using both 2D and 2.5D-to-3D input representations across five degenerative conditions: spinal canal stenosis, left and right neural foraminal narrowing, and left and right subarticular stenosis. The results showed that model behavior depended strongly on both architecture and input representation. Among the evaluated 2D pipelines, ConvNeXt achieved the strongest aggregate performance, while MaxViT showed the most competitive and stable behavior among the 2.5D-to-3D configurations.

The ordinal analysis demonstrated the importance of evaluating not only whether a prediction is correct but also how far an incorrect prediction lies from the reference severity grade. QWK, O-MAE, and SER revealed differences that were not apparent from ordinal accuracy alone. In particular, several 2.5D-to-3D configurations exhibited substantially weaker ordinal agreement and higher severe-error burden than their 2D counterparts, whereas MaxViT retained the strongest ordinal behavior within the stacked-input setting.

Condition-specific and spinal-level analyses further showed that performance was not uniformly distributed across either pathology or anatomical location. Different conditions acted as bottlenecks for different architectures, and performance varied across the five lumbar levels. These findings support the use of anatomically grounded evaluation rather than relying exclusively on global averages.

At the patient level, regrouping the condition–level outputs by subject provided an additional view of model behavior. Patient-level exactness, ordinal error magnitude, and maximum-error profiles distinguished examinations containing only adjacent-grade disagreements from those containing direct Normal/Mild–Severe errors. The illustrative case study demonstrated how the five condition-specific models can be combined into a structured patient profile spanning multiple conditions, spinal levels, imaging planes, and severity grades.

The comparison between 2D and 2.5D-to-3D representations also showed that additional through-plane context did not systematically improve performance in the present pipeline. This result should not be interpreted as evidence against volumetric modeling in general, but rather as an indication that stacked-context representations require careful architectural design, pretraining, optimization, and input construction. The present findings are therefore specific to the evaluated architecture–representation configurations.

Several limitations remain. The classification models rely on expert-provided localization coordinates, and the reported performance therefore reflects severity assessment under predefined regions of interest rather than fully automated end-to-end diagnosis. The repeated-run protocol captures training variability but does not establish robustness to external datasets, acquisition protocols, scanners, institutions, or ROI perturbations. In addition, the present comparison does not isolate dimensionality as a single causal factor because the 2D and 2.5D-to-3D pipelines may differ in pretraining and effective model capacity.

Future work should therefore extend the framework toward automated anatomical localization, controlled multiplanar fusion, matched volumetric comparisons, model calibration, uncertainty analysis, ROI perturbation robustness, and external multicenter validation. Such extensions would allow the proposed Beyond-Accuracy methodology to be evaluated under increasingly realistic clinical deployment conditions.

In summary, the study shows that lumbar spine degeneration models should be evaluated as structured prediction systems rather than as isolated classifiers. Combining exact, ordinal, condition-specific, anatomical, and patient-level analyses provides a substantially richer characterization of model behavior and offers a more informative basis for the development of radiologist-oriented decision-support systems.

## 7. Next Steps

The proposed Beyond-Accuracy framework provides a foundation for extending lumbar MRI severity classification beyond conventional predictive performance toward more reliable, anatomically informed, and clinically interpretable AI systems. Future research will focus on three complementary directions: prediction reliability and robustness, advanced imaging representations, and clinical decision-support integration.

### 7.1. Calibration and Localization Robustness

An important extension concerns the reliability of model confidence and its sensitivity to anatomical localization errors. Future experiments will evaluate probability calibration using Expected Calibration Error (ECE), the multiclass Brier score, and reliability diagrams to determine whether predicted confidence reflects observed classification correctness.

Additionally, robustness will be investigated by systematically perturbing the expert-provided ROI coordinates and measuring changes in exact classification and ordinal severity metrics. This analysis will help determine how classification performance deteriorates when ROI localization deviates from the reference anatomical position, an important consideration for future fully automated pipelines.

### 7.2. Multiplanar and Spatial Representation Analysis

Future work will investigate systematic comparisons between sagittal-only, axial-only, and explicitly fused multiplanar MRI representations for equivalent anatomical targets. Unlike condition-specific use of different imaging planes, true multiplanar fusion requires combining complementary views of the same anatomical target before severity prediction.

Further investigations will extend the comparison of 2D and cross-level 2.5D-to-3D architectures toward geometry-aware volumetric representations reconstructed from the original DICOM acquisitions. These experiments will examine whether increased anatomical context improves severity classification, ordinal consistency, and robustness while accounting for differences in model capacity, pretraining, and computational cost.

### 7.3. Clinical Decision-Support Integration

A longer-term objective is to translate the Beyond-Accuracy framework into a structured clinical decision-support interface. Rather than presenting isolated severity predictions, a future system could summarize degenerative conditions across lumbar levels while incorporating calibrated confidence, uncertainty estimates, and model validation information.

**Figure 24:**
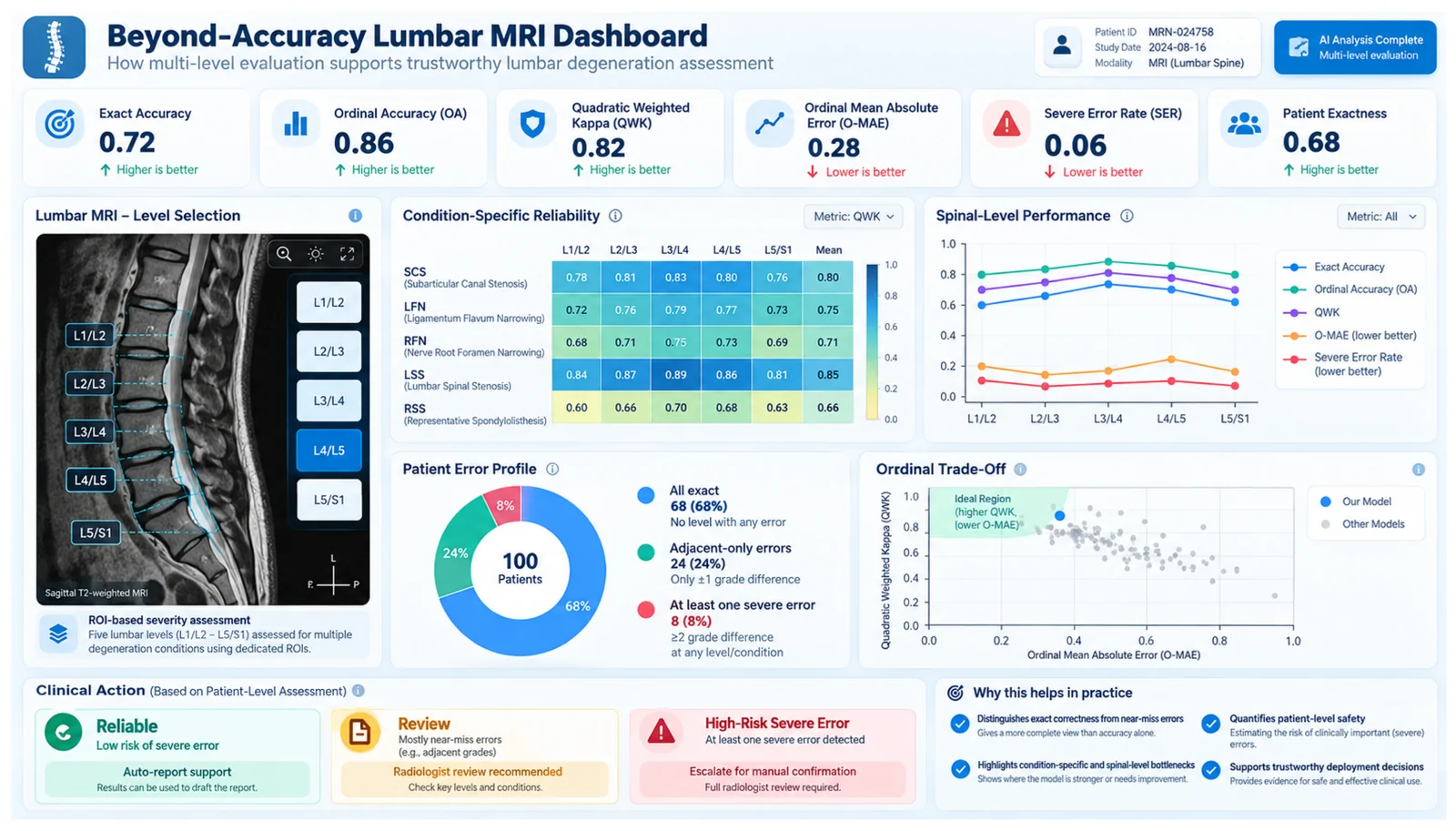
Conceptual clinical decision-support dashboard illustrating a potential application of the Beyond-Accuracy framework. The interface combines condition-specific severity predictions across lumbar levels with selected uncertainty and reliability indicators. Displayed values are illustrative and do not represent results from a prospective clinical deployment.

Figure 24 illustrates a conceptual dashboard integrating condition-specific severity predictions with patient-level reliability indicators. The interface is intended as a future application concept and does not represent a clinically validated deployment.

An important distinction is that population-level evaluation metrics, including Accuracy, Macro-F1, QWK, O-MAE, and SER, primarily support model development and validation. These metrics cannot directly establish the correctness of an individual prediction when reference labels are unavailable. Instead, a deployed interface could use calibrated probabilities, predictive uncertainty, and model disagreement to identify potentially unreliable predictions requiring additional radiological review.

The same framework could support periodic retrospective audits using clinically verified outcomes. Monitoring condition-specific, spinal-level, and ordinal performance would enable the identification of systematic weaknesses, dataset shifts, and opportunities for targeted model improvement.

Ultimately, prospective clinical validation will be necessary to determine whether such a system can improve diagnostic reliability, workflow efficiency, and human–AI interaction without introducing unnecessary automation bias.

### 7.4. Geometry-Aware 3D Dataset Expansion and Volumetric Modeling

The limitations observed with the exploratory cross-level 2.5D-to-3D representation motivate further investigation of geometry-aware volumetric MRI modeling. Although the current approach jointly processes five anatomically ordered lumbar-level ROIs, its artificial depth dimension does not preserve the physical spacing or spatial relationships of the original MRI acquisitions. Future work will therefore investigate anatomically aligned volumetric representations constructed using the original DICOM spatial metadata, with the aim of incorporating broader sagittal and axial MRI information and improving the utilization of the LumbarDISC dataset.

**Figure 25:**
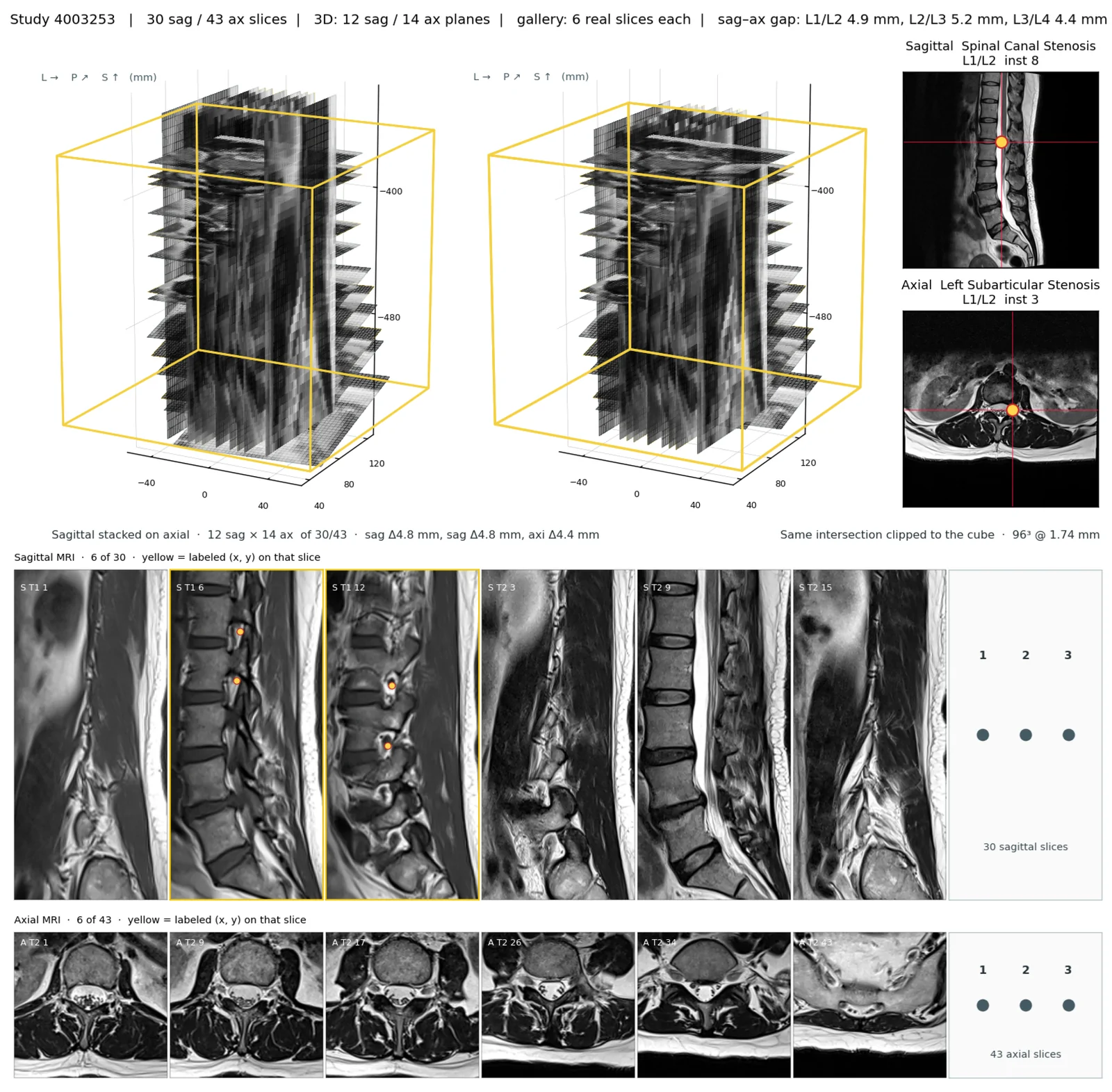
Ongoing investigation of geometry-aware 3D reconstruction and dataset expansion using sagittal and axial MRI acquisitions from the RSNA 2024 LumbarDISC dataset. The visualization illustrates the spatial intersection of sagittal and axial imaging planes, together with representative MRI slices and anatomical localization information. Arrows indicate the spatial relationships and correspondence between imaging views, while circular markers highlight anatomical locations or intersection points used for alignment. The yellow box identifies the region of interest considered for localized 3D representation. Additional symbols illustrate the geometric organization of the imaging planes. The proposed approach aims to support automatic ROI localization, anatomically aligned 3D representations, and expanded dataset utilization for volumetric deep learning. This figure presents an ongoing methodological direction rather than validated classification results.

Figure 25 illustrates this ongoing investigation. Sagittal and axial MRI slices are positioned according to their acquisition geometry, allowing their spatial intersections to be examined within a common coordinate system. The annotated anatomical locations serve as reference points for identifying corresponding lumbar regions across the two imaging planes. These relationships could enable localized volumetric representations that preserve more of the original spatial organization of the lumbar anatomy.

An important component of this extension is the development of automatic anatomical coordinate prediction. The current classification pipeline relies on expert-provided (x,y) coordinates to extract localized ROIs, which limits direct application to studies without corresponding localization annotations. We therefore aim to train localization models capable of estimating the relevant anatomical coordinates across sagittal and axial slices. These predictions would support automatic ROI extraction and facilitate the identification of corresponding anatomical regions across imaging planes, reducing dependence on manually provided localization information.

The predicted coordinates, together with available DICOM spatial metadata, could subsequently support the construction of localized volumetric inputs. By accounting for slice orientation, position, and spacing, sagittal and axial observations could be aligned within a shared patient-space reference system. The resulting representations would be evaluated using 3D neural architectures capable of extracting spatial features across multiple imaging planes. Particular attention will be given to geometric consistency, differences in slice spacing, incomplete anatomical coverage, and interpolation artifacts, since the original acquisitions do not necessarily form a uniformly sampled volumetric image.

Beyond improving anatomical representation, this strategy offers a potential pathway toward broader dataset utilization. Automatic coordinate prediction could extend localization to previously unannotated slices and studies, allowing a substantially larger portion of the original MRI acquisitions to be processed. However, coordinate localization alone does not provide missing severity labels. Consequently, the use of previously unlabeled data would require additional strategies, such as self-supervised representation learning, pseudo-labeling with confidence-based filtering, or subsequent expert validation. This distinction is important for separating dataset expansion from the generation of reliable classification targets.

Ultimately, the proposed extension pursues two complementary objectives: improving severity classification through anatomically aligned cross-plane information and increasing the amount of usable MRI data for model development. Future experiments will determine whether geometry-aware 3D representations outperform the current 2D and cross-level 2.5D-to-3D approaches under comparable evaluation protocols, particularly with respect to exact accuracy, ordinal severity agreement, and severe misclassification rates.

## Figures and Tables

**Table 1:** Validation-set exact classification performance across model architectures, input representations, and lumbar degenerative conditions. Values are reported as mean ± standard deviation across five repeated runs. ACC denotes Accuracy, P denotes Macro-Precision, R denotes Macro-Recall, and F1 denotes Macro-F1. The upward arrow (↑) indicates that higher values are preferable for the corresponding metric.

Architecture	Condition	ACC ↑	Precision ↑	Recall ↑	F1 ↑
**2D Models**					
ViT	SCS	0.9200±0.0059	0.7296±0.0398	0.6762±0.0253	0.6968±0.0142
	LFN	0.7726±0.0049	0.2575±0.0016	0.3333±0.0000	0.2906±0.0010
	RFN	0.8287±0.0354	0.6020±0.1953	0.5550±0.1310	0.5683±0.1612
	LSS	0.8294±0.0126	0.7235±0.0212	0.7069±0.0272	0.7128±0.0141
	RSS	0.8242±0.0060	0.7244±0.0225	0.6771±0.0195	0.6974±0.0185
	**Mean**	**0.8350**	**0.6074**	**0.5897**	**0.5932**
MaxViT	SCS	0.9178±0.0085	0.7351±0.0356	0.6478±0.0679	0.6737±0.0429
	LFN	0.8328±0.0102	0.6738±0.0554	0.6369±0.0507	0.6390±0.0341
	RFN	0.8345±0.0057	0.6712±0.0235	0.6169±0.0476	0.6357±0.0341
	LSS	0.8196±0.0096	0.6998±0.0314	0.6820±0.0194	0.6838±0.0072
	RSS	0.8185±0.0041	0.7109±0.0201	0.6618±0.0335	0.6805±0.0201
	**Mean**	**0.8446**	**0.6982**	**0.6491**	**0.6625**
ConvNeXt	SCS	0.9206±0.0041	0.7265±0.0183	0.6977±0.0129	0.7031±0.0147
	LFN	0.8468±0.0080	0.7319±0.0468	0.6170±0.0477	0.6508±0.0325
	RFN	0.8556±0.0079	0.7358±0.0344	0.6656±0.0486	0.6904±0.0253
	LSS	0.8441±0.0097	0.7624±0.0196	0.7076±0.0266	0.7297±0.0191
	RSS	0.8288±0.0053	0.7359±0.0267	0.6754±0.0375	0.6967±0.0213
	**Mean**	**0.8592**	**0.7385**	**0.6726**	**0.6941**
EfficientNet	SCS	0.9111±0.0147	0.6882±0.0522	0.6528±0.0278	0.6636±0.0330
	LFN	0.8342±0.0079	0.6669±0.0289	0.6749±0.0192	0.6652±0.0219
	RFN	0.8388±0.0117	0.6972±0.0148	0.6357±0.0572	0.6523±0.0483
	LSS	0.8227±0.0126	0.6910±0.0171	0.7084±0.0071	0.6904±0.0183
	RSS	0.8101±0.0079	0.6815±0.0229	0.6757±0.0142	0.6740±0.0167
	**Mean**	**0.8434**	**0.6850**	**0.6695**	**0.6691**
**2.5D-to-3D Models**					
ViT	SCS	0.8765±0.0081	0.5558±0.0467	0.4121±0.0304	0.4302±0.0380
	LFN	0.7729±0.0058	0.3969±0.0995	0.3351±0.0017	0.2949±0.0039
	RFN	0.7787±0.0094	0.3907±0.0777	0.3437±0.0086	0.3148±0.0174
	LSS	0.7508±0.0140	0.5786±0.0423	0.4743±0.0327	0.4830±0.0392
	RSS	0.7304±0.0215	0.5457±0.0325	0.4553±0.0292	0.4669±0.0215
	**Mean**	**0.7819**	**0.4935**	**0.4041**	**0.3980**
MaxViT	SCS	0.8974±0.0032	0.6788±0.0363	0.5977±0.0580	0.6258±0.0405
	LFN	0.8200±0.0075	0.6523±0.0497	0.5620±0.0545	0.5906±0.0598
	RFN	0.8203±0.0109	0.6626±0.0287	0.5963±0.0448	0.6117±0.0298
	LSS	0.7848±0.0108	0.6331±0.0124	0.6152±0.0281	0.6166±0.0237
	RSS	0.7858±0.0057	0.6638±0.0151	0.6173±0.0216	0.6336±0.0191
	**Mean**	**0.8217**	**0.6581**	**0.5977**	**0.6156**
ConvNeXt	SCS	0.8730±0.0045	0.2910±0.0015	0.3333±0.0000	0.3107±0.0008
	LFN	0.7726±0.0049	0.2575±0.0016	0.3333±0.0000	0.2906±0.0010
	RFN	0.7772±0.0104	0.2591±0.0035	0.3333±0.0000	0.2915±0.0022
	LSS	0.7225±0.0081	0.2408±0.0027	0.3333±0.0000	0.2796±0.0018
	RSS	0.7057±0.0249	0.2850±0.0607	0.3693±0.0532	0.3189±0.0535
	**Mean**	**0.7702**	**0.2667**	**0.3405**	**0.2983**
EfficientNet	SCS	0.8730±0.0045	0.2910±0.0015	0.3333±0.0000	0.3107±0.0008
	LFN	0.7836±0.0202	0.4048±0.1569	0.3635±0.0629	0.3366±0.0905
	RFN	0.7772±0.0104	0.2591±0.0035	0.3333±0.0000	0.2915±0.0022
	LSS	0.7489±0.0216	0.4855±0.1659	0.4373±0.0800	0.4341±0.1109
	RSS	0.7955±0.0083	0.6827±0.0452	0.6155±0.0453	0.6288±0.0234
	**Mean**	**0.7956**	**0.4246**	**0.4166**	**0.4004**

**Table 2:** Validation-set ordinal severity performance across model architectures, input representations, and lumbar degenerative conditions. Values are reported as mean ± standard deviation across five repeated runs. OA denotes Ordinal Accuracy, O-MAE denotes Ordinal Mean Absolute Error, QWK denotes Quadratic Weighted Kappa, and SER denotes Severe Ordinal Error Rate. The upward arrow (↑) indicates that higher values are preferable (OA and QWK), whereas the downward arrow (↓) indicates that lower values are preferable (O-MAE and SER).

Architecture	Condition	OA ↑	O-MAE ↓	QWK ↑	SER ↓
**2D Models**					
ViT	SCS	0.9200±0.0059	0.0859±0.0053	0.7883±0.0211	0.0059±0.0014
	LFN	0.7726±0.0049	0.2678±0.0050	0.0000±0.0000	0.0405±0.0019
	RFN	0.8287±0.0354	0.1819±0.0506	0.5259±0.2969	0.0106±0.0154
	LSS	0.8294±0.0126	0.1766±0.0136	0.7631±0.0163	0.0061±0.0012
	RSS	0.8242±0.0060	0.1838±0.0057	0.7431±0.0162	0.0080±0.0019
	**Mean**	**0.8350**	**0.1792**	**0.5641**	**0.0142**
MaxViT	SCS	0.9178±0.0085	0.0907±0.0104	0.7549±0.0685	0.0086±0.0043
	LFN	0.8328±0.0102	0.1714±0.0116	0.6664±0.0359	0.0042±0.0021
	RFN	0.8345±0.0057	0.1704±0.0060	0.6435±0.0274	0.0049±0.0017
	LSS	0.8196±0.0096	0.1900±0.0097	0.7437±0.0261	0.0096±0.0022
	RSS	0.8185±0.0041	0.1902±0.0044	0.7345±0.0221	0.0088±0.0028
	**Mean**	**0.8446**	**0.1626**	**0.7086**	**0.0072**
ConvNeXt	SCS	0.9206±0.0041	0.0855±0.0055	0.7984±0.0130	0.0061±0.0054
	LFN	0.8468±0.0080	0.1572±0.0092	0.6634±0.0177	0.0041±0.0016
	RFN	0.8556±0.0079	0.1483±0.0075	0.6905±0.0254	0.0039±0.0013
	LSS	0.8441±0.0097	0.1609±0.0111	0.7752±0.0168	0.0050±0.0026
	RSS	0.8288±0.0053	0.1779±0.0026	0.7526±0.0150	0.0067±0.0035
	**Mean**	**0.8592**	**0.1460**	**0.7360**	**0.0052**
EfficientNet	SCS	0.9111±0.0147	0.0971±0.0145	0.7660±0.0251	0.0081±0.0013
	LFN	0.8342±0.0079	0.1709±0.0085	0.6792±0.0094	0.0051±0.0016
	RFN	0.8388±0.0117	0.1652±0.0120	0.6568±0.0219	0.0041±0.0010
	LSS	0.8227±0.0126	0.1874±0.0165	0.7611±0.0200	0.0100±0.0046
	RSS	0.8101±0.0079	0.2028±0.0088	0.7276±0.0104	0.0129±0.0017
	**Mean**	**0.8434**	**0.1647**	**0.7182**	**0.0081**
**2.5D-to-3D Models**					
ViT	SCS	0.8765±0.0081	0.1672±0.0124	0.2916±0.0784	0.0438±0.0052
	LFN	0.7729±0.0058	0.2673±0.0059	0.0063±0.0044	0.0402±0.0022
	RFN	0.7787±0.0094	0.2593±0.0118	0.0378±0.0322	0.0381±0.0035
	LSS	0.7508±0.0140	0.2938±0.0262	0.4404±0.0611	0.0446±0.0129
	RSS	0.7304±0.0215	0.3257±0.0305	0.3776±0.0454	0.0561±0.0092
	**Mean**	**0.7819**	**0.2627**	**0.2308**	**0.0446**
MaxViT	SCS	0.8974±0.0032	0.1187±0.0064	0.6490±0.0577	0.0162±0.0045
	LFN	0.8200±0.0075	0.1904±0.0087	0.5605±0.0535	0.0104±0.0026
	RFN	0.8203±0.0109	0.1869±0.0099	0.6010±0.0397	0.0072±0.0032
	LSS	0.7848±0.0108	0.2408±0.0164	0.6417±0.0356	0.0257±0.0090
	RSS	0.7858±0.0057	0.2327±0.0055	0.6484±0.0155	0.0185±0.0051
	**Mean**	**0.8217**	**0.1939**	**0.6201**	**0.0156**
ConvNeXt	SCS	0.8730±0.0045	0.1770±0.0032	0.0000±0.0000	0.0500±0.0026
	LFN	0.7726±0.0049	0.2678±0.0050	0.0000±0.0000	0.0405±0.0019
	RFN	0.7772±0.0104	0.2622±0.0118	0.0000±0.0000	0.0394±0.0028
	LSS	0.7225±0.0081	0.3648±0.0068	0.0000±0.0000	0.0873±0.0047
	RSS	0.7057±0.0249	0.3847±0.0416	0.1106±0.1521	0.0905±0.0340
	**Mean**	**0.7702**	**0.2913**	**0.0221**	**0.0615**
EfficientNet	SCS	0.8730±0.0045	0.1770±0.0032	0.0000±0.0000	0.0500±0.0026
	LFN	0.7836±0.0202	0.2501±0.0349	0.1114±0.2370	0.0337±0.0148
	RFN	0.7772±0.0104	0.2622±0.0118	0.0000±0.0000	0.0394±0.0028
	LSS	0.7489±0.0216	0.3061±0.0400	0.3253±0.2246	0.0550±0.0201
	RSS	0.7955±0.0083	0.2233±0.0096	0.6615±0.0368	0.0188±0.0032
	**Mean**	**0.7956**	**0.2437**	**0.2196**	**0.0394**

**Table 3:** Condition-specific validation performance across the evaluated architectures and input representations. Condition-macro values are computed from the mean performance obtained for SCS, LFN, RFN, LSS, and RSS across repeated runs. For Macro-F1 and QWK, higher values are preferred; for O-MAE and SER, lower values are preferred. $\Delta_{\mathrm{cond}}$ denotes the range between the maximum and minimum condition-specific values, and $\sigma_{\mathrm{cond}}$ denotes the standard deviation across the five conditions.

Representation	Architecture	Metric	Cond. Mean	Best Condition	Worst Condition	$\Delta_{\mathrm{cond}}$	$\sigma_{\mathrm{cond}}$
2D Models							
2D	ViT	Macro-F1	0.6068	LSS (0.7214)	LFN (0.2916)	0.4298	0.1822
		QWK	0.5753	SCS (0.7891)	LFN (0.0000)	0.7891	0.3353
		O-MAE	0.1739	SCS (0.0850)	LFN (0.2626)	0.1776	0.0631
		SER	0.0144	LSS (0.0053)	LFN (0.0400)	0.0347	0.0144
2D	MaxViT	Macro-F1	0.6797	SCS (0.7026)	LFN (0.6516)	0.0509	0.0183
		QWK	0.7242	SCS (0.7871)	LFN (0.6633)	0.1238	0.0506
		O-MAE	0.1577	SCS (0.0882)	LSS (0.1957)	0.1075	0.0426
		SER	0.0064	LFN (0.0034)	RSS (0.0096)	0.0062	0.0030
2D	ConvNeXt	Macro-F1	0.6739	LSS (0.7293)	LFN (0.5286)	0.2008	0.0827
		QWK	0.6864	SCS (0.7948)	LFN (0.4179)	0.3769	0.1560
		O-MAE	0.1503	SCS (0.0795)	LFN (0.1920)	0.1125	0.0432
		SER	0.0080	RFN (0.0031)	LFN (0.0178)	0.0147	0.0058
2D	EfficientNet	Macro-F1	0.6812	LSS (0.6932)	LFN (0.6591)	0.0340	0.0131
		QWK	0.7192	SCS (0.7582)	LFN (0.6733)	0.0848	0.0375
		O-MAE	0.1610	SCS (0.0932)	RSS (0.2010)	0.1078	0.0421
		SER	0.0084	LFN (0.0030)	RSS (0.0139)	0.0109	0.0045
2.5D-to-3D Models							
2.5D-to-3D	ViT	Macro-F1	0.4018	RSS (0.4793)	LFN (0.3201)	0.1592	0.0729
		QWK	0.2258	RSS (0.3908)	LFN (0.0460)	0.3447	0.1599
		O-MAE	0.2597	SCS (0.1639)	RSS (0.3105)	0.1466	0.0599
		SER	0.0450	RFN (0.0354)	LSS (0.0566)	0.0213	0.0093
2.5D-to-3D	MaxViT	Macro-F1	0.5956	LSS (0.6296)	LFN (0.5496)	0.0800	0.0307
		QWK	0.6021	LSS (0.6485)	RFN (0.5507)	0.0979	0.0461
		O-MAE	0.1923	SCS (0.1211)	RSS (0.2362)	0.1151	0.0468
		SER	0.0178	LFN (0.0089)	LSS (0.0254)	0.0165	0.0076
2.5D-to-3D	ConvNeXt	Macro-F1	0.2909	SCS (0.3112)	RSS (0.2797)	0.0315	0.0129
		QWK	0.0000	– (0.0000)	– (0.0000)	0.0000	0.0000
		O-MAE	0.2867	SCS (0.1734)	LSS (0.3702)	0.1968	0.0837
		SER	0.0627	RFN (0.0389)	LSS (0.0935)	0.0546	0.0278
2.5D-to-3D	EfficientNet	Macro-F1	0.4184	RSS (0.6375)	RFN (0.2922)	0.3453	0.1391
		QWK	0.2584	RSS (0.6658)	RFN (0.0000)	0.6658	0.2791
		O-MAE	0.2387	SCS (0.1734)	LSS (0.3108)	0.1374	0.0508
		SER	0.0389	RSS (0.0177)	LSS (0.0618)	0.0442	0.0174

**Table 4:** Validation-set spinal-level performance of the 2D models. Values are reported as mean ± standard deviation across repeated runs after macro-averaging the five degenerative conditions at each spinal level. Macro-F1 and QWK are higher-is-better metrics, whereas O-MAE and SER are lower-is-better. Mean denotes the equal-weight average across the five lumbar levels, and $\Delta_{\mathrm{level}}$ denotes the range between the maximum and minimum level-specific values.

Architecture	Metric	L1/L2	L2/L3	L3/L4	L4/L5	L5/S1	Mean	$\Delta_{\mathrm{level}}$
ViT	Macro-F1	0.473±0.032	0.556±0.020	0.596±0.040	0.608±0.041	0.523±0.056	0.551	0.136
	QWK	0.454±0.077	0.493±0.041	0.566±0.062	0.586±0.062	0.440±0.099	0.508	0.146
	O-MAE	0.045±0.004	0.102±0.003	0.188±0.013	0.279±0.023	0.255±0.018	0.174	0.235
	SER	0.003±0.002	0.004±0.001	0.007±0.003	0.021±0.006	0.037±0.009	0.014	0.034
MaxViT	Macro-F1	0.387±0.031	0.498±0.021	0.665±0.032	0.696±0.009	0.574±0.041	0.564	0.309
	QWK	0.285±0.080	0.478±0.040	0.675±0.027	0.742±0.011	0.600±0.053	0.556	0.457
	O-MAE	0.052±0.003	0.107±0.006	0.167±0.014	0.244±0.013	0.206±0.009	0.155	0.192
	SER	0.005±0.002	0.005±0.001	0.002±0.002	0.007±0.001	0.013±0.003	0.007	0.011
ConvNeXt	Macro-F1	0.478±0.057	0.578±0.046	0.650±0.052	0.682±0.038	0.577±0.051	0.593	0.203
	QWK	0.499±0.117	0.597±0.100	0.649±0.061	0.693±0.068	0.539±0.073	0.595	0.194
	O-MAE	0.046±0.008	0.095±0.011	0.171±0.011	0.238±0.024	0.210±0.035	0.152	0.192
	SER	0.002±0.001	0.003±0.002	0.004±0.003	0.012±0.007	0.022±0.012	0.009	0.019
EfficientNet	Macro-F1	0.394±0.032	0.531±0.019	0.630±0.027	0.676±0.021	0.591±0.028	0.565	0.282
	QWK	0.222±0.066	0.513±0.030	0.679±0.024	0.721±0.019	0.600±0.059	0.547	0.499
	O-MAE	0.057±0.004	0.106±0.006	0.173±0.013	0.265±0.011	0.211±0.012	0.162	0.208
	SER	0.007±0.001	0.006±0.001	0.004±0.003	0.011±0.004	0.014±0.004	0.008	0.010

**Table 5:** Validation-set spinal-level performance of the 2.5D-to-3D models. Values are reported as mean ± standard deviation across repeated runs after macro-averaging the five degenerative conditions at each spinal level. Macro-F1 and QWK are higher-is-better metrics, whereas O-MAE and SER are lower-is-better. Mean denotes the equal-weight average across the five lumbar levels, and $\Delta_{\mathrm{level}}$ denotes the range between the maximum and minimum level-specific values.

Architecture	Metric	L1/L2	L2/L3	L3/L4	L4/L5	L5/S1	Mean	$\Delta_{\mathrm{level}}$
ViT	Macro-F1	0.330±0.009	0.332±0.022	0.364±0.033	0.402±0.026	0.302±0.015	0.346	0.100
	QWK	0.006±0.012	0.058±0.050	0.176±0.072	0.255±0.048	0.047±0.033	0.108	0.249
	O-MAE	0.063±0.005	0.148±0.012	0.287±0.018	0.462±0.019	0.339±0.011	0.260	0.399
	SER	0.011±0.002	0.021±0.003	0.038±0.012	0.084±0.009	0.071±0.004	0.045	0.074
MaxViT	Macro-F1	0.412±0.035	0.459±0.032	0.514±0.022	0.608±0.036	0.512±0.038	0.501	0.195
	QWK	0.251±0.073	0.377±0.063	0.487±0.038	0.603±0.058	0.441±0.047	0.432	0.352
	O-MAE	0.055±0.004	0.124±0.006	0.234±0.014	0.312±0.021	0.237±0.019	0.192	0.257
	SER	0.007±0.002	0.011±0.002	0.017±0.005	0.029±0.005	0.020±0.005	0.017	0.022
ConvNeXt	Macro-F1	0.325±0.001	0.311±0.001	0.283±0.001	0.242±0.003	0.281±0.002	0.288	0.082
	QWK	0.000±0.000	0.000±0.000	0.000±0.000	0.000±0.000	0.000±0.000	0.000	0.000
	O-MAE	0.062±0.005	0.152±0.012	0.319±0.009	0.569±0.019	0.339±0.014	0.288	0.507
	SER	0.011±0.001	0.025±0.005	0.060±0.005	0.148±0.010	0.076±0.005	0.064	0.137
EfficientNet	Macro-F1	0.333±0.011	0.343±0.019	0.367±0.045	0.380±0.069	0.328±0.035	0.350	0.051
	QWK	0.032±0.034	0.096±0.054	0.184±0.104	0.235±0.129	0.096±0.062	0.129	0.203
	O-MAE	0.060±0.007	0.137±0.011	0.267±0.010	0.433±0.039	0.321±0.020	0.243	0.373
	SER	0.010±0.002	0.018±0.002	0.034±0.010	0.083±0.021	0.065±0.009	0.042	0.073

**Table 6:** Validation-set patient-level performance across the evaluated architectures and input representations. Patient Exact denotes the patient-macro exact-match rate; O-MAE denotes the patient-macro ordinal mean absolute error. $R_{0}$, $R_{1}$, and $R_{2}$ denote the proportions of patients whose maximum ordinal error is 0, 1, or 2, respectively. Thus, $R_{0}$ corresponds to completely correct patient assessments, $R_{1}$ to patients affected only by adjacent-grade errors, and $R_{2}$ to patients containing at least one direct Normal/Mild–Severe error. Values are mean ± standard deviation across repeated runs.

Representation	Architecture	Runs	Patient Exact	Patient O-MAE	$R_{0}$	$R_{1}$	$R_{2}$
2D Models							
2D	ViT	5	0.840±0.008	0.174±0.011	0.452±0.016	0.464±0.024	0.084±0.019
2D	MaxViT	5	0.853±0.004	0.154±0.005	0.458±0.010	0.498±0.017	0.043±0.008
2D	ConvNeXt	5	0.858±0.009	0.149±0.012	0.485±0.011	0.466±0.012	0.049±0.021
2D	EfficientNet	5	0.849±0.004	0.159±0.004	0.439±0.007	0.510±0.006	0.051±0.004
2.5D-to-3D Models							
2.5D-to-3D	ViT	5	0.786±0.004	0.258±0.004	0.371±0.015	0.407±0.023	0.221±0.010
2.5D-to-3D	MaxViT	5	0.826±0.002	0.191±0.004	0.421±0.009	0.482±0.013	0.097±0.017
2.5D-to-3D	ConvNeXt	4	0.778±0.004	0.284±0.004	0.390±0.003	0.351±0.003	0.259±0.001
2.5D-to-3D	EfficientNet	4	0.800±0.003	0.239±0.006	0.393±0.006	0.418±0.013	0.189±0.008

**Table 7:** Beyond-Accuracy patient-level summary for the illustrative 25-assessment case study.

Patient-Level Quantity	Result
Degenerative conditions	5
Lumbar levels per condition	5
Valid condition–level assessments	25
Exact predictions	24
Adjacent-grade errors	1
Severe two-grade errors	0
Patient Exact $A_{p}$	0.960
Patient O-MAE	0.040
Maximum ordinal error $E_{p}^{\max}$	1
Patient error profile	$R_{1}$: Adjacent-only

**Table 8:** Estimated computational cost of the complete NAS on an eight-GPU system. Cloud cost assumes an eight-GPU Vast.ai instance price of USD 3.70 h^−1^.

Family	Trials/Cond.	Mean Trial Time	8-GPU Wall Time	Est. Cost (USD)
ViT 2D	2304	3.18 min	76.4 h	282.7
ViT-3D	2304	2.35 min	56.4 h	208.7
MaxViT 2D	1152	3.05 min	36.6 h	135.4
MaxViT-3D	1152	2.37 min	28.5 h	105.5
ConvNeXt 2D	144	1.55 min	2.3 h	8.5
ConvNeXt-3D	384	1.62 min	6.5 h	24.1
EfficientNet 2D	144	1.13 min	1.7 h	6.3
EfficientNet-3D	384	2.23 min	8.9 h	32.9
2D families	3744	—	117.0 h	432.9
2.5D-to-3D families	4224	—	100.3 h	371.1
All eight families	7968	—	217.3 h	804.0

**Figure A1:**
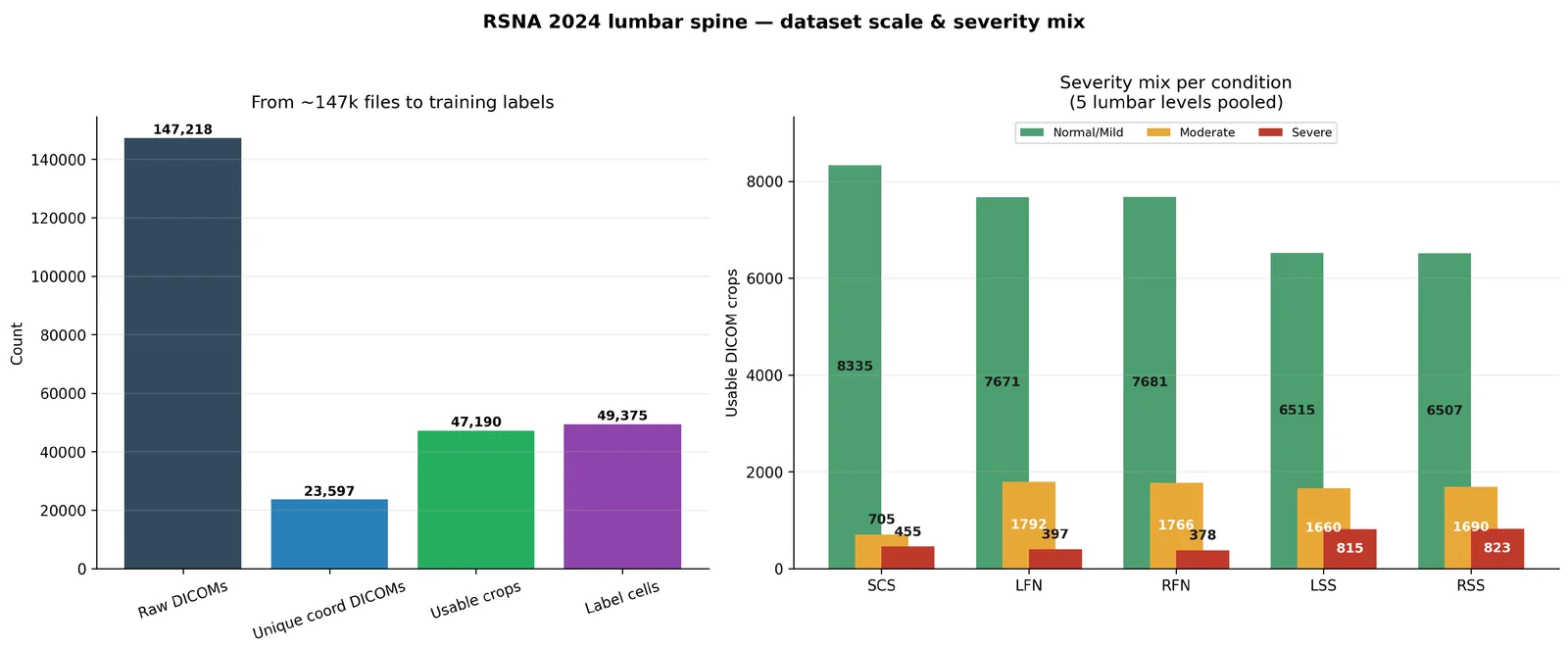
High-level organization and severity distribution of the RSNA 2024 LumbarDISC data used in this study. The left panel illustrates the transition from the complete DICOM archive to coordinate-linked images, derived anatomical crops, and condition–level severity labels. The right panel shows the pooled severity distribution across the five lumbar levels for spinal canal stenosis (SCS), left and right neural foraminal narrowing (LFN and RFN), and left and right subarticular stenosis (LSS and RSS). Normal/Mild findings dominate all five conditions, whereas Severe cases are substantially less represented.

**Figure A2:**
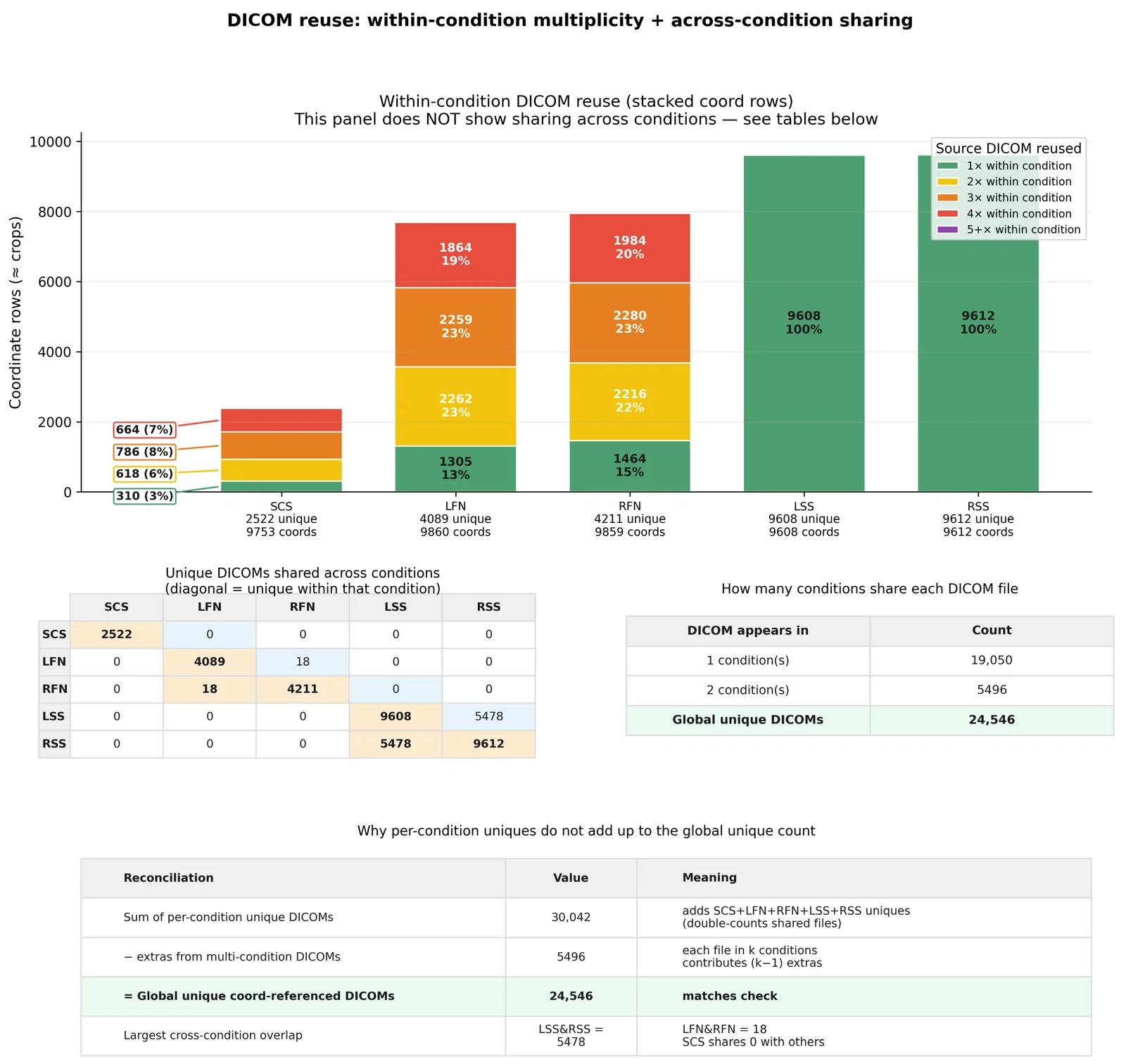
Coordinate-annotation multiplicity and cross-condition DICOM reuse in the RSNA 2024 LumbarDISC dataset. The upper panel illustrates within-condition reuse of source DICOM instances, while the lower panels summarize pairwise cross-condition overlap and the number of conditions referencing each unique DICOM instance. In the lower-left panel, the color gradient represents the magnitude of DICOM instance overlap between pairs of degenerative conditions, with lighter colors indicating lower overlap and darker colors indicating higher overlap.

**Figure A3:**
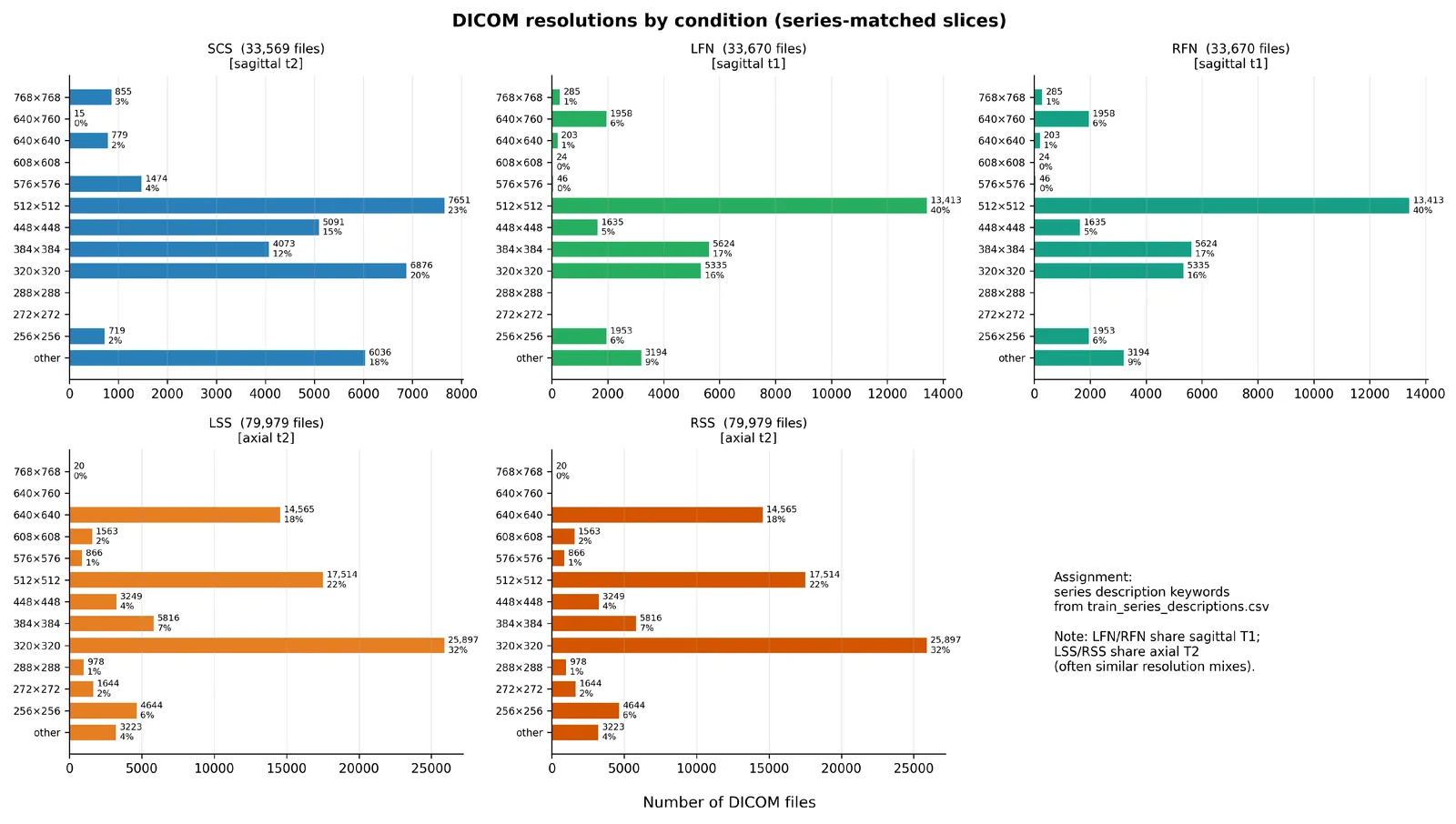
Distribution of native DICOM matrix dimensions for the MRI series associated with each degenerative condition. Sagittal pipelines are predominantly represented by 512×512 images, whereas 320×320 is the most frequent matrix size for the axial LSS and RSS pipelines. The distributions illustrate the heterogeneous acquisition matrices encountered before coordinate-centered ROI extraction.

**Figure A4:**
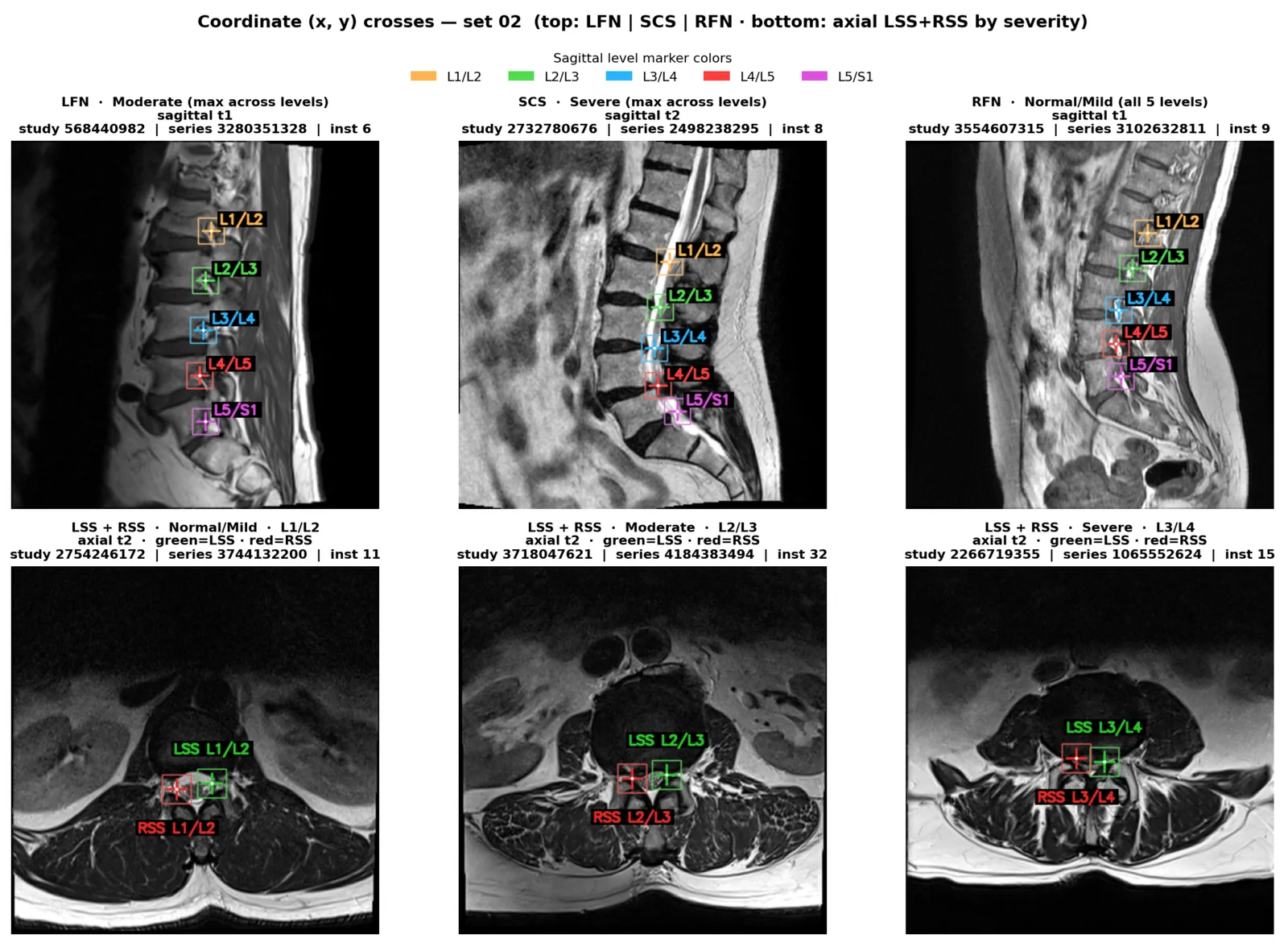
Representative coordinate annotations from the RSNA 2024 LumbarDISC dataset. The upper row presents sagittal T1- and T2-weighted MRI examples for left neural foraminal narrowing (LFN), spinal canal stenosis (SCS), and right neural foraminal narrowing (RFN), with markers identifying the five lumbar intervertebral levels. The lower row illustrates axial T2-weighted examples in which left and right subarticular stenosis coordinates may occur on the same DICOM instance. Cross (+) symbols indicate annotated anatomical coordinates, while colored boxes highlight the corresponding localized regions of interest used for image extraction and classification.

**Figure A5:**
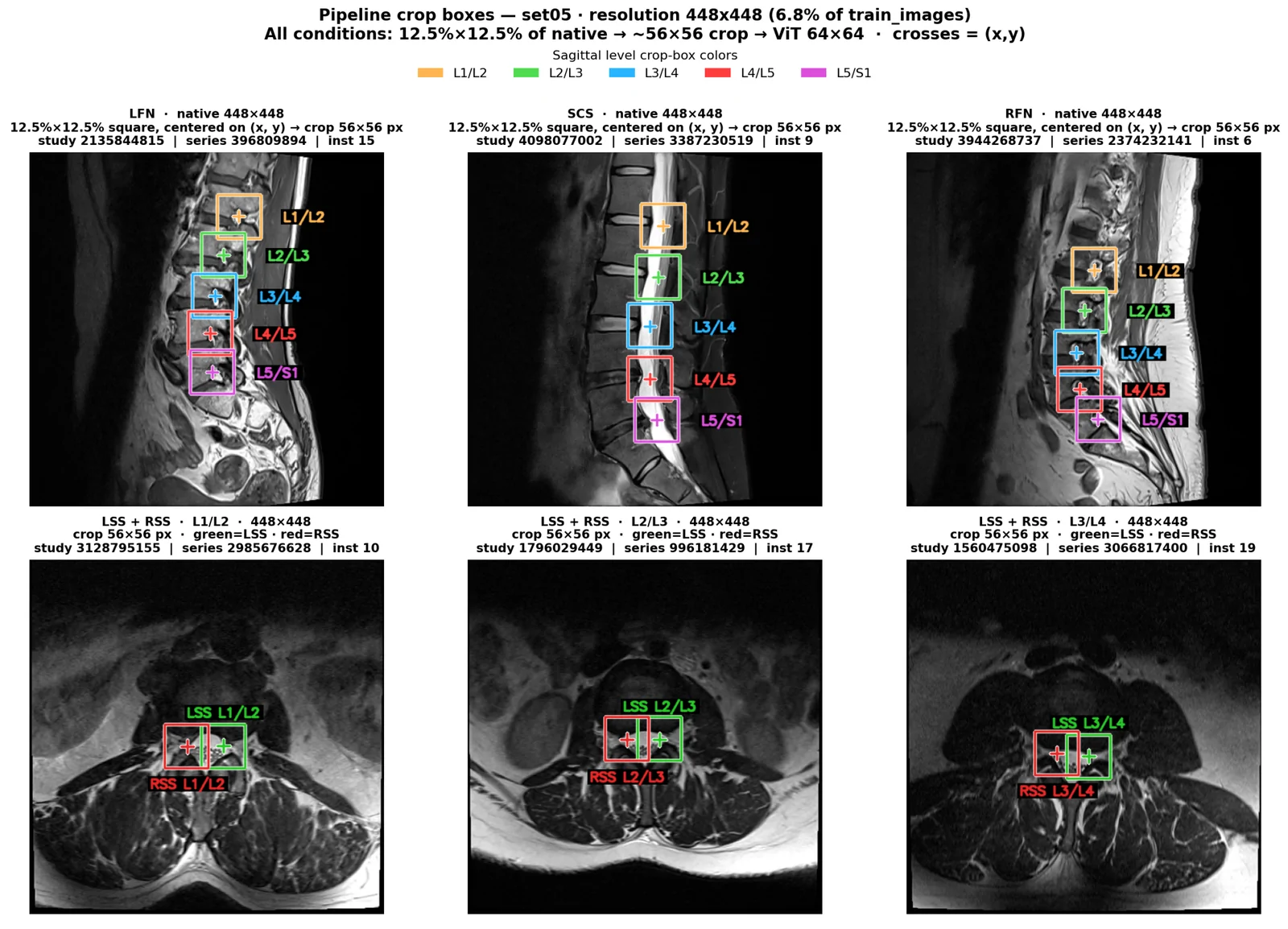
Representative coordinate-centered ROI construction for native 448×448 DICOM images. The cross (+) symbol indicates the expert-provided anatomical (x,y) coordinate, while the colored bounding box delineates the region of interest (ROI) extracted around that coordinate. Each crop is centered on the annotation and spans 12.5% of the native image width and height, corresponding to an approximately 56×56 pixel region at this resolution. The extracted ROI is subsequently resized to 64×64 pixels for model input.

**Figure A6:**
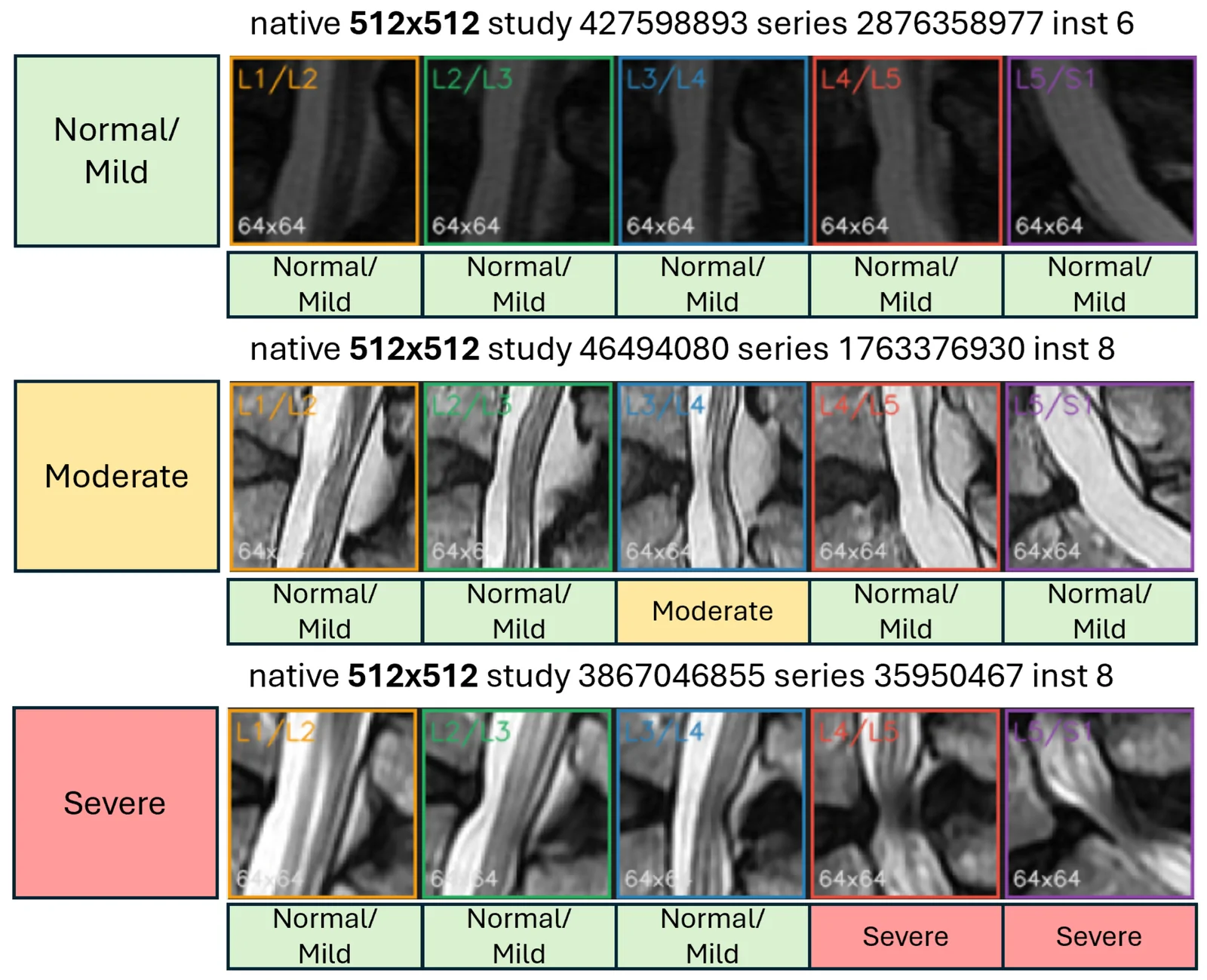
Representative cross-level and cross-severity assessment of standardized 64×64 ROIs extracted from a native 512×512 resolution group. Columns correspond to the five lumbar levels from L1/L2 to L5/S1, while rows illustrate cases grouped by the maximum severity observed across the five levels. Labels beneath individual crops retain the original level-specific ground-truth severity.

**Figure A7:**
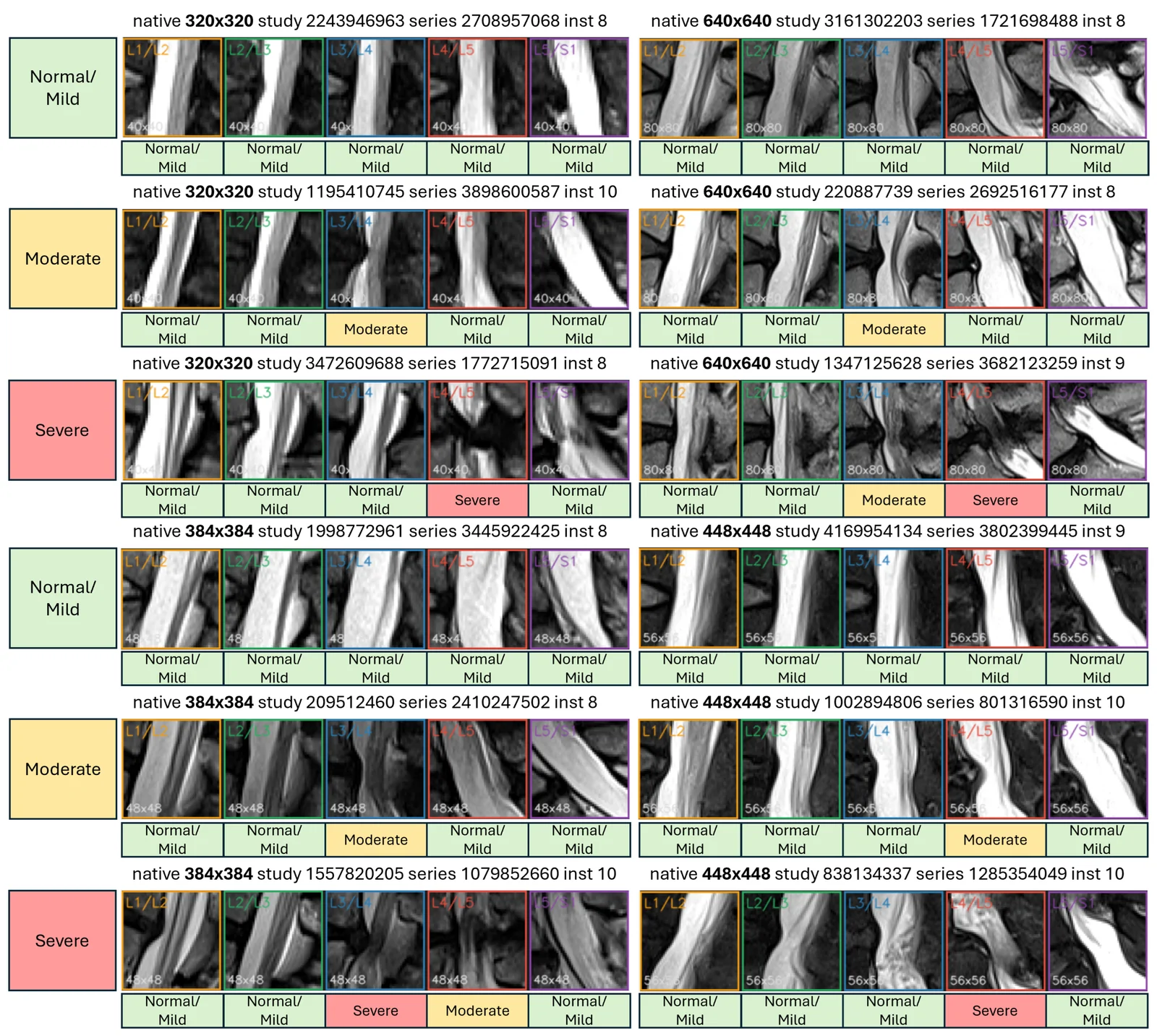
Cross -resolution and cross-level qualitative assessment of standardized spinal canal stenosis ROIs. Representative native 320×320, 384×384, 448×448, and 640×640 images produce native crops of 40×40, 48×48, 56×56, and 80×80 pixels, respectively, before standardization to 64×64. The recurring level-dependent anatomical patterns provide a qualitative check that the proportional cropping strategy preserves local lumbar geometry across heterogeneous native image matrices.

## Data Availability

The medical imaging data analyzed in this study are publicly available through the RSNA 2024 Lumbar Spine Degenerative Classification dataset. The source code used for preprocessing, neural architecture search, model training, repeated evaluation, and multi-level analysis is publicly available at https://github.com/trincema-research/medical-imaging-pipelines/tree/main/rsna2024_lumbar, accessed on 20 October 2026. Processed intermediate data and derived experimental results can be made available by the corresponding author upon reasonable request, subject to the terms and conditions governing redistribution of the original dataset.

## Abbreviations

The following abbreviations are used in this manuscript:

2.5D	Two-and-a-Half-Dimensional Imaging Representation
AdamW	Adam Optimizer with Decoupled Weight Decay
AI	Artificial Intelligence
AMP	Automatic Mixed Precision
AUC	Area Under the Curve
AUROC	Area Under the Receiver Operating Characteristic Curve
CNN	Convolutional Neural Network
CSV	Comma-Separated Values
DICOM	Digital Imaging and Communications in Medicine
FN	False Negative
FP	False Positive
GELU	Gaussian Error Linear Unit
GPU	Graphics Processing Unit
Grad-CAM	Gradient-weighted Class Activation Mapping
LFN	Left Neural Foraminal Narrowing
LLM	Large Language Model
LR	Learning Rate
LSS	Left Subarticular Stenosis
MAE	Mean Absolute Error
MRI	Magnetic Resonance Imaging
NAS	Neural Architecture Search
OA	Ordinal Accuracy
O-MAE	Ordinal Mean Absolute Error
QWK	Quadratic Weighted Kappa
RFN	Right Neural Foraminal Narrowing
ROI	Region of Interest
ROC	Receiver Operating Characteristic
RSNA	Radiological Society of North America
RSS	Right Subarticular Stenosis
SCS	Spinal Canal Stenosis
SER	Severe Error Rate
TN	True Negative
TP	True Positive
ViT	Vision Transformer
WD	Weight Decay
XAI	Explainable Artificial Intelligence

## Appendix A. Dataset Characterization and ROI Preprocessing Details

This appendix provides supplementary characterization of the RSNA 2024 LumbarDISC dataset and additional details of the preprocessing pipeline used to construct the model-ready inputs. The main Methodology retains only the information required to understand the experimental design, ROI construction, and study-level data partitioning. The analyses reported here provide additional reproducibility information regarding the hierarchical dataset structure, severity distributions, coordinate-annotation multiplicity, cross-condition DICOM reuse, native image-resolution distributions, anatomical coordinate organization, proportional ROI construction, and qualitative validation of the standardized 64×64 model inputs. These analyses support the preprocessing procedure but do not constitute separate predictive experiments or model-performance results.

### Appendix A.1. Dataset Structure, Annotation Availability, and Severity Distribution

The RSNA 2024 Lumbar Spine Degenerative Classification dataset, subsequently formalized as the Lumbar Degenerative Imaging Spine Classification (LumbarDISC) dataset, contains lumbar MRI examinations acquired using multiple sequences and imaging planes. The dataset has a hierarchical organization in which each MRI study contains one or more imaging series, and each series contains multiple DICOM instances.

The complete training archive contains 147,218 raw DICOM files. Of these, 23,597 unique DICOM instances remain directly associated with usable spatial coordinate annotations after preprocessing. Processing the available annotations produces approximately 47,190 usable condition–level image crops and 49,375 condition–level severity label cells.

The five lumbar levels considered are

L={L1/L2,L2/L3,L3/L4,L4/L5,L5/S1},

and the condition set is

C={SCS,LFN,RFN,LSS,RSS}.

Each condition–level pair is assigned an ordinal severity label

$$y_{c,l}\in \left\{0,1,2\right\},\;0=\mathrm{Normal}/\mathrm{Mild},\;1=\mathrm{Moderate},\;2=\mathrm{Severe}.$$

A complete examination may therefore contain up to 25 condition–level severity assessments, corresponding to five conditions evaluated across five lumbar levels.

When the five levels are pooled, spinal canal stenosis contains 8335 Normal/Mild, 705 Moderate, and 455 Severe samples. The corresponding distributions are 7671/1792/397 for LFN, 7681/1766/378 for RFN, 6515/1660/815 for LSS, and 6507/1690/823 for RSS. These distributions illustrate the marked imbalance between the Normal/Mild and Severe categories and motivate condition-specific training and evaluation.

### Appendix A.2. Coordinate Annotation Multiplicity and Cross-Condition DICOM Reuse

The coordinate annotation structure introduces an important distinction between annotation rows, unique DICOM instances, and final usable condition–level samples. The raw coordinate table contains 48,692 annotation rows distributed across the five conditions as 9753 SCS, 9860 LFN, 9859 RFN, 9608 LSS, and 9612 RSS records.

After the complete DICOM-resolution and study-usability preprocessing pipeline, 47,190 condition–level samples remain. The reduction from the raw coordinate records reflects annotations that do not survive the full preprocessing and usability criteria.

A single source DICOM can contribute to multiple coordinate annotations. Within a condition, the mean annotation multiplicity per unique DICOM is approximately 3.87 for SCS, 2.41 for LFN, 2.34 for RFN, and 1.00 for both LSS and RSS.

Cross-condition DICOM reuse also occurs. The most notable overlap is between left and right subarticular stenosis, where the same axial T2-weighted image can contain coordinate annotations for both sides of the spinal canal.

The raw coordinate table references approximately 24,546 globally unique DICOM instances, whereas 23,597 unique coordinate-linked DICOMs remain after the complete usability-filtering stage. These quantities therefore describe different stages of the preprocessing pipeline and should not be interpreted as independent datasets.

### Appendix A.3. Native DICOM Resolution Distribution

The dataset contains MRI examinations acquired using heterogeneous scanner configurations and reconstruction protocols, resulting in substantial variability in native DICOM matrix dimensions.

Spinal canal stenosis is primarily associated with sagittal T2-weighted imaging, left and right neural foraminal narrowing with sagittal T1-weighted imaging, and left and right subarticular stenosis with axial T2-weighted imaging.

The sagittal series exhibit a strong concentration around 512×512, whereas axial subarticular-stenosis series are frequently represented by 320×320 images. These dimensions describe dominant native acquisition patterns rather than fixed requirements imposed on the original DICOM files.

The proportional ROI extraction described below avoids assuming a single native image resolution because the crop dimensions are computed directly from the size of each source image.

### Appendix A.4. Spatial Coordinate Annotations and Lumbar-Level Localization

The dataset provides spatial annotations linking selected DICOM instances to specific lumbar levels and degenerative conditions. Each annotation can be represented as

$$a_{i}=\left(s_{i},\;r_{i},\;k_{i},\;c_{i},\;l_{i},\;x_{i},\;y_{i}\right),$$

where $s_{i}$ denotes the study identifier, $r_{i}$ the series identifier, $k_{i}$ the DICOM instance number, $c_{i}$ the degenerative condition, $l_{i}$ the lumbar level, and $\left(x_{i},y_{i}\right)$ the annotated spatial coordinate.

The DICOM instance number identifies the particular image within the selected MRI series, while the in-plane coordinate identifies the corresponding local anatomical region.

For sagittal conditions, several lumbar levels may be annotated on the same or closely related slices. For axial subarticular stenosis, left and right coordinates may occur on the same DICOM instance, reflecting the paired anatomical organization of the two lateral recesses.

These expert-provided annotations form the basis of the ROI construction used throughout the present experiments.

### Appendix A.5. Coordinate-Centered ROI Extraction and Cross-Resolution Validation

For an original DICOM image

$$I\in R^{H\times W},$$

and an annotated spatial coordinate

p=(x,y),

a rectangular ROI is centered on the annotated location. The crop width and height are defined as fixed fractions of the native image dimensions,

$$w_{\mathrm{ROI}}=0.125W,\;h_{\mathrm{ROI}}=0.125H.$$

The crop boundaries are therefore

$$x_{\min}=x-\frac{w_{\mathrm{ROI}}}{2},\;x_{\max}=x+\frac{w_{\mathrm{ROI}}}{2},$$

and

$$y_{\min}=y-\frac{h_{\mathrm{ROI}}}{2},\;y_{\max}=y+\frac{h_{\mathrm{ROI}}}{2}.$$

where necessary, the limits are clipped to the valid image boundaries. The resulting native crop is subsequently resized to

$$R_{\mathrm{model}}\in R^{64\times 64}.$$

The complete transformation can therefore be summarized as

$$I\overset{12.5\%\;\times \;12.5\%\;\mathrm{native}\;\mathrm{crop}}{\underset{\mathrm{centered}\;\mathrm{at}\;(x,y)}{\to }}R\overset{\mathrm{resize}}{\to }R_{\mathrm{model}}^{64\times 64}.$$

Because the crop size is proportional to the native matrix dimensions, different acquisition resolutions naturally produce different native crop sizes before standardization. Representative examples are summarized in Table A1.

The crop overlays were visually inspected across the dominant native resolution groups to verify that the annotated location remained approximately centered and that comparable anatomical context was retained after standardization.

**Table A1:** Representative native image resolutions used to validate the coordinate-centered cropping strategy. The native ROI is computed as 12.5% of the source image dimensions and subsequently standardized to the common 64×64 model input size.

Set	Native Resolution	Share	Native Crop → Model Input
set01	512×512	26.2%	64×64→64×64
set02	320×320	25.9%	40×40→64×64
set03	640×640	10.6%	80×80→64×64
set04	384×384	10.5%	48×48→64×64
set05	448×448	6.8%	56×56→64×64

### Appendix A.6. Cross-Level and Cross-Severity ROI Quality Control

Following validation of the crop geometry, an additional qualitative analysis was performed to determine whether the standardized ROIs preserved coherent anatomical structure across lumbar levels, native resolutions, and severity categories.

For visualization purposes, cases were grouped according to the maximum severity observed across their five lumbar levels,

$$s_{\max}=\max_{l\in L}s_{l},$$

where $s_{l}\in \left\{0,1,2\right\}$ denotes the level-specific Normal/Mild, Moderate, or Severe grade. Importantly, this case-level grouping was used only for visualization and did not replace the original level-specific labels used during model training.

These visual assessments are intended exclusively as qualitative preprocessing quality-control analyses. They verify the preservation of anatomical localization and recurrent level-dependent structure after ROI standardization and should not be interpreted as evidence of class separability or predictive model performance.

## Appendix B. Detailed Mathematical Formulation of the Vision Transformer Pipeline

This appendix provides the mathematical formulation and intermediate tensor representations of the condition-specific Vision Transformer (ViT) pipeline introduced in Section 3.4 and illustrated in Figure 3. The description follows the six processing stages, from localized MRI crop preprocessing to three-class severity prediction.

### Appendix B.1. MRI Crop Input and Preprocessing

Each condition-specific model receives a localized grayscale MRI crop centered on the annotated coordinate of one of the five intervertebral levels: L1/L2, L2/L3, L3/L4, L4/L5, or L5/S1.

The crop is standardized to a spatial resolution of H×W=64×64 pixels and initially contains a single intensity channel:

$$X_{\mathrm{MRI}}\in R^{1\times 64\times 64}.$$

To ensure compatibility with ImageNet-pretrained ViT backbones configured for three-channel inputs, the grayscale channel is replicated three times:

$$X\in R^{3\times 64\times 64}.$$

For a mini-batch of *B* samples, the corresponding input tensor has dimensions

$$X_{B}\in R^{B\times 3\times 64\times 64}.$$

Channel replication does not introduce additional imaging information. Instead, the original MRI intensity pattern is preserved identically across all three channels, allowing the pretrained patch-embedding layer to process the grayscale image without modifying its expected input-channel dimensionality.

### Appendix B.2. Patch Partitioning

The standardized MRI crop is partitioned into non-overlapping square patches of size P×P. The total number of image patches is calculated as

(A1) $$N_{p}=\frac{H}{P}\times \frac{W}{P},$$

where *H* and *W* denote the image height and width, respectively, and *P* is the patch size.

For the ViT-B/16 and ViT-L/16 configurations, P=16, producing

$$N_{p}=\frac{64}{16}\times \frac{64}{16}=4\times 4=16.$$

Each patch therefore has dimensions 3×16×16, corresponding to

3×16×16=768

scalar intensity values.

For the /32 variants, the patch size increases to 32×32, resulting in

$$N_{p}=\frac{64}{32}\times \frac{64}{32}=2\times 2=4.$$

Thus, the /32 configurations process fewer image tokens while representing the same localized MRI crop at a coarser spatial resolution.

### Appendix B.3. Patch Embedding

Each image patch is flattened into a one-dimensional vector before being transformed into a learned embedding representation.

For the *i*-th patch, the flattened vector is

$$x_{p}^{i}\in R^{CP^{2}},$$

where C=3 represents the number of replicated input channels.

For a 16×16 patch,

$$x_{p}^{i}\in R^{768}.$$

The flattened patch is projected into the Transformer embedding space using a learned linear transformation:

(A2) $$e_{i}=x_{p}^{i}W+b,$$

where

$$W\in R^{CP^{2}\times D},\;b\in R^{D},$$

and *D* denotes the embedding dimensionality.

The resulting patch embedding satisfies

$$e_{i}\in R^{D}.$$

For all patches extracted from one MRI crop, the complete embedding sequence is represented as

$$E=\left[e_{1},e_{2},\ldots ,e_{N_{p}}\right]\in R^{N_{p}\times D}.$$

For ViT-B/16, where D=768, the embedding sequence becomes

$$E\in R^{16\times 768}.$$

Although the flattened patch vector and its projected embedding both contain 768 elements in this particular configuration, they represent different quantities. The flattened vector contains raw MRI intensity values, whereas the projected embedding represents learned visual features.

### Appendix B.4. Classification Token and Positional Encoding

A learnable classification token, denoted by [CLS], is prepended to the sequence of patch embeddings:

$$X_{\mathrm{cls}}=\left[x_{\mathrm{cls}};e_{1};e_{2};\ldots ;e_{N_{p}}\right].$$

This operation increases the sequence length from $N_{p}$ to $N_{p}+1$.

For a mini-batch of *B* samples, the resulting representation has dimensions

$$X_{\mathrm{cls}}\in R^{B\times (N_{p}+1)\times D}.$$

Accordingly, the /16 configurations contain 17 tokens, while the /32 configurations contain five tokens.

Because standard Transformer self-attention does not inherently preserve the original spatial position of each image patch, learnable positional embeddings are added to the token sequence.

The positional embedding tensor is defined as

$$E_{\mathrm{pos}}\in R^{1\times (N_{p}+1)\times D}.$$

The initial Transformer input is then obtained through

(A3) $$Z_{0}=X_{\mathrm{cls}}+E_{\mathrm{pos}},$$

where

$$Z_{0}\in R^{B\times (N_{p}+1)\times D}.$$

For ViT-B/16, the resulting tensor dimensions are

$$Z_{0}\in R^{B\times 17\times 768}.$$

This representation combines learned patch-level features with information about their spatial arrangement within the localized lumbar MRI crop.

### Appendix B.5. Transformer Encoder

The embedded token sequence is processed by *L* stacked Transformer encoder blocks, each containing multi-head self-attention, a feed-forward network, layer normalization, and residual connections.

At a high level, the sequence evolves as

$$Z_{0}\to Z_{1}\to \cdots \to Z_{L}.$$

Within each encoder block, self-attention enables every token to incorporate information from other tokens in the sequence, allowing the model to capture relationships between different anatomical regions of the MRI crop.

For a standard multi-head self-attention operation, query, key, and value representations are obtained through learned projections of the input sequence:

$$Q=ZW_{Q},\;K=ZW_{K},\;V=ZW_{V}.$$

For an individual attention head, scaled dot-product attention is defined as

(A4) $$\mathrm{Attention}\left(Q,K,V\right)=\mathrm{Softmax}\left(\frac{QK^{T}}{\sqrt{d_{k}}}\right)V,$$

where $d_{k}$ denotes the dimensionality of the key vectors for that attention head.

The outputs of multiple attention heads are concatenated and projected before being passed through the remaining encoder operations.

The final encoder output retains the general tensor structure

$$Z_{L}\in R^{B\times (N_{p}+1)\times D}.$$

For a single sample, the final classification-token representation is extracted as

$$h_{\mathrm{cls}}=Z_{L}^{\left(\mathrm{cls}\right)}\in R^{D}.$$

This representation serves as a global feature descriptor of the localized MRI crop, aggregating information across the complete patch sequence.

### Appendix B.6. Condition-Specific Severity Classification

The final classification-token representation is passed to a trainable classification head that maps the *D*-dimensional feature vector into three output logits:

(A5) $$z=W_{h}h_{\mathrm{cls}}+b_{h},$$

where

$$W_{h}\in R^{3\times D},\;b_{h}\in R^{3},\;z\in R^{3}.$$

The three logits correspond to the ordered severity categories Normal/Mild, Moderate, and Severe.

The predicted class is determined by

$$\hat{y}=\arg\max_{k\in \{0,1,2\}}z_{k}.$$

When class probabilities are required for inference or probability-based evaluation, the logits are normalized using the Softmax function:

$$p_{k}=\frac{\exp(z_{k})}{\sum_{j=0}^{2}\exp\left(z_{j}\right)}.$$

During training, cross-entropy loss is applied directly to the raw logits. Therefore, explicit Softmax normalization is not required within the trainable classification head.

Each lumbar condition is modeled independently, using the same processing pipeline but a separate condition-specific classifier.

### Appendix B.7. Architectural Variants

The general ViT pipeline remains unchanged across the investigated configurations. However, the architectures differ in patch size, embedding dimensionality, encoder depth, number of attention heads, and total parameter count.

The /16 configurations generate 16 image patches, while the /32 configurations generate four. Including the classification token, the corresponding sequence lengths are seventeen and five.

ViT-B uses an embedding dimensionality of D=768, whereas ViT-L uses a larger embedding space and deeper Transformer encoder.

These architectural differences influence model capacity and computational requirements without changing the fundamental processing stages: MRI crop preprocessing, patch partitioning, patch embedding, positional encoding, Transformer feature extraction, and condition-specific severity prediction.

## Appendix C. Neural Architecture Search Hyperparameter Spaces

This appendix documents the discrete hyperparameter spaces used during the neural architecture search (NAS) for the four architecture families evaluated in the study: ViT, MaxViT, ConvNeXt, and EfficientNet. The search was conducted separately for conventional 2D processing and cross-level 2.5D-to-3D representations, allowing architecture-specific configurations to be investigated under the same general experimental framework.

The search spaces include architectural variants, optimizer choices, learning rates, weight-decay coefficients, and classification-head configurations. For selected families, classification-head depth, hidden dimensions, dropout, and activation functions were also varied. These parameters were included because they influence both optimization behavior and the capacity of the final severity classifier.

The search spaces were not identical across all architecture families. Transformer-based models were evaluated over broader optimizer and learning-rate ranges, whereas the convolutional families used more restricted configurations. Consequently, the number of candidate trials differs between architectures. The following tables document the parameter choices used in each experimental setting.

### Appendix C.1. 2D Architecture Families and Search Spaces

The 2D search space covers four architecture families applied to localized lumbar MRI regions of interest. Each candidate configuration processes an individual ROI and predicts one of the three ordered severity categories: Normal/Mild, Moderate, or Severe. The architecture search was performed independently for each of the five degenerative conditions.

For ViT, four variants were considered, combining Base and Large model capacities with patch sizes of 16 and 32 pixels. MaxViT was evaluated using Tiny and Small variants. These Transformer-based families were investigated using AdamW and SGD, with multiple learning-rate and weight-decay settings. The classification-head search additionally considered alternative depths and hidden dimensions, providing flexibility in adapting pretrained representations to the lumbar severity classification task.

The ConvNeXt and EfficientNet search spaces were more compact. They used AdamW optimization and examined alternative classification-head depths, hidden dimensions, dropout probabilities, and activation functions. These configurations enabled a comparison of convolutional architectures with Transformer-based models while avoiding identical search-space assumptions across substantially different network families.

Table A2 reports the complete 2D search spaces. The specified trial counts were 2304 for ViT, 1152 for MaxViT, and 144 each for ConvNeXt and EfficientNet, corresponding to 3744 designed candidate trials per degenerative condition.

**Table A2:** Hyperparameter search spaces used for the 2D architecture families.

Family	Variants	Optimizer/Learning Rate	Weight Decay	Classification-Head Search	Trials/Condition
ViT	ViT-B/16, ViT-L/16, ViT-B/32, ViT-L/32	**AdamW:** $5\times 10^{-5}$, $1\times 10^{-4}$, $2\times 10^{-4}$, $4\times 10^{-4}$, $8\times 10^{-4}$, $1.2\times 10^{-3}$, $1.6\times 10^{-3}$, $2.4\times 10^{-3}$. **SGD:** $1.6\times 10^{-3}$, $3.2\times 10^{-3}$, $4.8\times 10^{-3}$, $8\times 10^{-3}$, $1.2\times 10^{-2}$, $1.6\times 10^{-2}$, $2.4\times 10^{-2}$, $3.2\times 10^{-2}$.	**AdamW/SGD:** 0 0.001 0.005 0.01 0.02 0.05 0.08 0.1	**AdamW:** Depth {2,3}; Hidden {none,128,256,512}. **SGD:** Depth 2; Hidden 256.	2304
MaxViT	MaxViT-Tiny, MaxViT-Small	Same AdamW and SGD learning-rate grids as ViT.	**AdamW/SGD:** 0 0.001 0.005 0.01 0.02 0.05 0.08 0.1	Same Transformer head search as ViT.	1152
ConvNeXt	ConvNeXt-Small	**AdamW:** $8\times 10^{-4}$ $1.6\times 10^{-3}$	**AdamW:** 0 0.01 0.05	Depth {2,3,4}; Hidden {none,256}; Dropout {0.1,0.2}; Activation {GELU,ReLU}.	144
EfficientNet	EfficientNet-V2-S	**AdamW:** $8\times 10^{-4}$ $1.6\times 10^{-3}$	**AdamW:** 0 0.01 0.05	Depth {2,3,4}; Hidden {none,256}; Dropout {0.1,0.2}; Activation {GELU,ReLU}.	144

#### Cross-Level 2.5D-to-3D Architecture Families and Search Spaces

The second search space was designed for the cross-level 2.5D-to-3D representation. Unlike the conventional 2D setting, these models jointly process five localized ROIs corresponding to the anatomically ordered lumbar levels L1/L2 through L5/S1. The stacked tensor is processed using a 3D encoder, followed by level-specific severity classification heads.

The purpose of this additional search was to investigate the behavior of the proposed evaluation framework under a structurally different input representation. The cross-level configuration was treated as an exploratory architectural comparison, rather than as a geometry-preserving volumetric MRI reconstruction or explicit multiplanar fusion.

The ViT-3D search considered Tiny, Small, Base, and Large variants, while MaxViT-3D included Tiny and Small configurations. Both families were evaluated using AdamW and SGD, with learning-rate ranges adapted to their respective 3D implementations. Classification-head depth and hidden dimensions were also included in the search, allowing different model capacities and optimization settings to be examined.

For ConvNeXt-3D and EfficientNet-3D, the search included AdamW and SGD configurations with optimizer-specific weight decay and dropout choices. Classification-head depth, hidden dimensions, and activation functions were also varied. Compared with the corresponding 2D convolutional search spaces, these configurations introduced additional optimizer combinations for the cross-level models.

Table A3 presents the complete cross-level search spaces. The specified trial counts were 2304 for ViT-3D, 1152 for MaxViT-3D, and 384 each for ConvNeXt-3D and EfficientNet-3D, corresponding to 4224 designed candidate trials per degenerative condition.

**Table A3:** Hyperparameter search spaces used for the cross-level 2.5D-to-3D architecture families.

Family	Variants	Optimizer/Learning Rate	Weight Decay	Classification-Head Search	Trials/ Condition
ViT-3D	ViT3D-T, ViT3D-S, ViT3D-B, ViT3D-L	AdamW: $1\times 10^{-5}$, $3\times 10^{-5}$, $5\times 10^{-5}$, $1\times 10^{-4}$, $2\times 10^{-4}$, $3\times 10^{-4}$, $5\times 10^{-4}$, $8\times 10^{-4}$. SGD: $3\times 10^{-4}$, $8\times 10^{-4}$, $1.6\times 10^{-3}$, $3.2\times 10^{-3}$, $4.8\times 10^{-3}$, $8\times 10^{-3}$, $1.2\times 10^{-2}$, $1.6\times 10^{-2}$.	AdamW/SGD: 0 0.001 0.005 0.01 0.02 0.05 0.08 0.1	AdamW: Depth {2,3}; Hidden {none,128,256,512}. SGD: Depth 2; Hidden 256.	2304
MaxViT-3D	MaxViT3D-Tiny, MaxViT3D-Small	Same AdamW and SGD learning-rate grids as ViT-3D.	AdamW/SGD: 0 0.001 0.005 0.01 0.02 0.05 0.08 0.1	Same Transformer head search as ViT-3D.	1152
ConvNeXt-3D	CNN3D-S, CNN3D-B	AdamW: $8\times 10^{-4}$ $1.6\times 10^{-3}$ SGD: $4.8\times 10^{-3}$ $1.6\times 10^{-2}$	AdamW: 0 0.01 0.05 SGD: 0 0.01	Depth {2,3,4}; Hidden {none,256}; Activation {GELU,ReLU}; AdamW dropout {0.1,0.2}; SGD dropout 0.1.	384
EfficientNet-3D	CNN3D-S, CNN3D-B	AdamW: $8\times 10^{-4}$ $1.6\times 10^{-3}$ SGD: $4.8\times 10^{-3}$ $1.6\times 10^{-2}$	AdamW: 0 0.01 0.05 SGD: 0 0.01	Depth {2,3,4}; Hidden {none,256}; Activation {GELU,ReLU}; AdamW dropout {0.1,0.2}; SGD dropout 0.1.	384

### Appendix C.2. Search-Space Coverage and Interpretation

Across both input representations, the search comprised 7968 designed candidate configurations per condition. Applying these search spaces to the five degenerative conditions produced a total of 39,840 designed NAS trials. These counts describe the experimental search design and should be distinguished from the number of successfully completed training executions.

The architecture-dependent search spaces reflect differences in model structure, optimizer behavior, and computational requirements. In particular, the broader Transformer search spaces and more restricted convolutional configurations should not be interpreted as equivalent computational budgets or as an isolated comparison of architectural capacity.

Candidate configurations were selected using validation accuracy, as described in the main Methodology. The subsequent Beyond-Accuracy evaluation considered conventional classification metrics together with ordinal severity errors and anatomically structured prediction behavior. The complete parameter definitions in this appendix support reproduction of the search procedure while keeping implementation-oriented details separate from the principal experimental findings.
